# Organocatalytic Access to Enantioenriched Spirooxindole-Based 4-Methyleneazetidines

**DOI:** 10.3390/molecules22112016

**Published:** 2017-11-21

**Authors:** Giulia Rainoldi, Matteo Faltracco, Claudia Spatti, Alessandra Silvani, Giordano Lesma

**Affiliations:** 1 Department of Chemistry, University of Milan, via Golgi 19, 20133 Milan, Italy; giulia.rainoldi@unimi.it (G.R.); claudia.spatti@studenti.unimi.it (C.S.); giordano.lesma@unimi.it (G.L.); 2 Department of Chemistry & Pharmaceutical Sciences, Amsterdam Institute of Molecules Medicines & Systems (AIMMS), Vrije Universiteit Amsterdam, De Boelelaan 1108, 1081 HZ Amsterdam, The Netherlands; matteofaltracco92@gmail.com

**Keywords:** spirooxindoles, organocatalysis, azetidines, cinchona-based catalysts

## Abstract

This work describes the synthesis of enantioenriched spiro compounds, incorporating the azetidine and the oxindole motifs. The preparation relies on a formal [2 + 2] annulation reaction of isatin-derived *N*-*tert*-butylsulfonyl ketimines with allenoates. The asymmetric induction is secured by an organocatalytic strategy, exploiting a bifunctional cinchona-type β-isocupridine-based catalyst. Some post-transformation products, including unexpected spiropyrroline and 3,3-disubstituted oxindole derivatives, are also presented.

## 1. Introduction

Spirocyclic scaffolds are privileged in drug discovery, thanks to the conformational restriction imparted by the spiro carbon, which provides an excellent strategy to improve ligand–protein binding in many different therapeutic areas [1,2].

Among spirocycles, spirooxindoles are compounds characterized by the presence of a spiro ring fused at the C3 position of the oxindole core. A high number of them have been reported to exert various biological activities (i.e., antitumoral [3], anti-inflammatory [4] and antiviral [5]), quite often associated with a relevant target-specificity. The varied bioactivities of spirooxindoles are mainly distinguished by the kind of spiro ring fused at the C3 position, as well as by substituent moieties both on the spiro ring and on the oxindole scaffold. These structural elements lead to the diverse but relatively specific pharmacological profiles of this class of privileged molecules.

Owing to the therapeutic potential and, at the same time, to the synthetic challenge associated with spirooxindoles, there is a huge interest to develop new synthetic methodologies [6], in particular those achieving asymmetric induction by catalytic protocols [7,8].

Since the most widely exploited scaffolds so far involve the spiro junction of oxindoles with five-membered and six-membered rings, we recently aimed to contribute to greater diversity in this field turning our attention to four membered rings. With the notable exception of β-lactams, four-membered rings have received limited attention from medicinal chemists until recently, with only a few spirocyclic azetidine scaffolds proposed as new potential lead compounds [9,10].

**Scheme 1:**
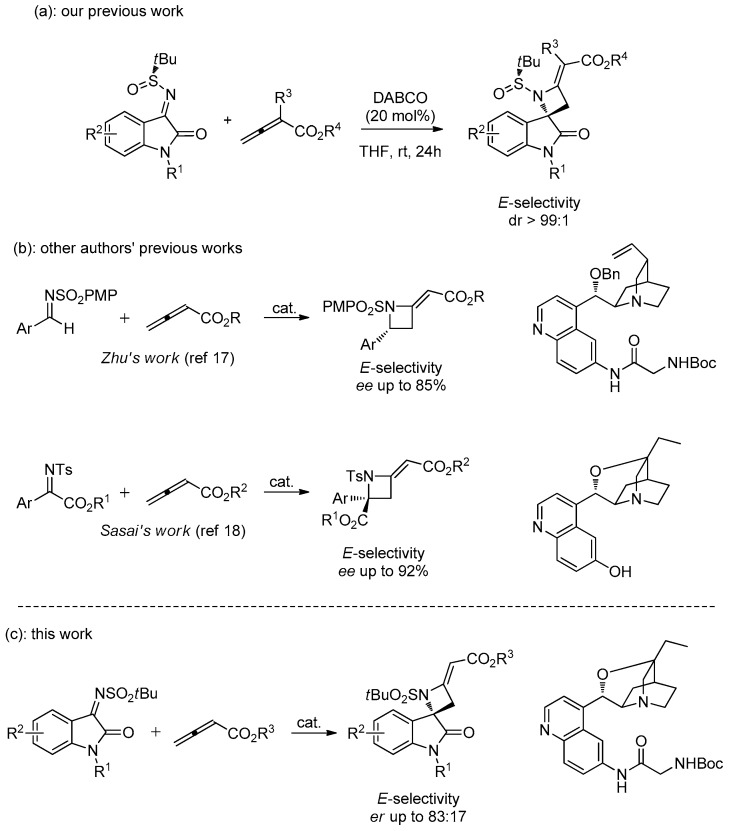
(**a**) Our previous diastereoselective strategy to chiral spirooxindole-based 4-methyleneazetidines; (**b**) Reported organocatalyzed formal [2 + 2] cycloadditions of aldimmines and ketimines with allenoates; (**c**) Organocatalysed enantioselective strategy to chiral spirooxindole-based 4-methyleneazetidines.

In our ongoing work devoted to the asymmetric synthesis of spirooxindoles [11,12], we recently reported the first highly diastereoselective entry into chiral spirooxindole-based 4-methyleneazetidines [13]. Such compounds can be considered new lead structures in drug discovery and also versatile intermediates, thanks to the presence of the reactive carbon-carbon double bond and of the carboxylic ester moiety (Scheme 1a).

As a continuation of this previous achievement, we have regarded the advantages of catalytic methods and faced the challenge of the catalytic asymmetric construction of such spirooxindole-based azetidine ring system.

Among catalytic methods, organocatalysis has brought unprecedented progress to the asymmetric construction of spirooxindoles [14] and 3-amino-2-oxindoles bearing tetra-substituted stereocenters [15,16]. It is especially attractive due to the general availability and stability of organocatalysts and mild and simple reaction conditions used.

The first asymmetric organocatalytic synthesis of 2,4-disubstituted azetidines was reported by Zhu et al. [17] in 2011 (Scheme 1b). It relies on the formal [2 + 2] cycloaddition of *N*-sulfonyl-aldimines and allenoates using a novel bifunctional quinidine derivative as catalyst. A variety of aromatic *N*-sulfonyl-aldimines underwent cycloaddition with allenoates to afford *R*-configured azetidines in good yields and good regio- and enantioselectivities. Two years later, Sasai et al. [18] presented the first example of an enantioselective organocatalytic cycloaddition of allenoates involving ketimines, and thus affording azetidines with a chiral tetrasubstituted carbon stereogenic center (Scheme 1b).

Starting from isatin-derived *N*-*tert*-butylsulfonyl ketimines, we considered their organocatalyzed, formal [2 + 2] annulation reaction with allenoates as a high atom-economy strategy to obtain functionalized chiral spirooxindole-based 4-methyleneazetidines. Here we demonstrate the suitability of bifunctional cinchona alkaloids derivatives to promote such annulation, leading for the first time to enantioenriched azetidine-based spiro compounds by means of an organocatalytic method (Scheme 1c).

## 2. Results and Discussion

We started our investigation evaluating the reaction of ethyl buta-2,3-dienoate (**4a**) with different *N*-benzylisatin-derived ketimines [19], namely *N*-*tert*-butylsulfonyl imine **1a**, *N*-*p*-toluenesulfonyl imine **2** and *N*-Boc imine **3** (Table 1).

All substrates **1a**, **2** and **3** afforded the corresponding azetidines **6a**, **7** and **8** in presence of DABCO (entries 1–3). In the case of *N*-Boc ketimine **3**, also the aza-Morita-Baylis-Hillman (AMBH) product **9** was isolated in low yield. Then the same ketimines **1a**, **2** and **3** have been tested in the organocatalytic reaction, employing the quinidine-derived alkaloid β-isocupridine (β-ICD) catalyst **5a**. Both *N*-*tert*-butylsulfonyl imine **1a** and tosyl imine **2** afforded the expected azetidines **6a** and **7** in good yield and enantiomeric ratio (er) (entries 4 and 5), whereas in this condition the *N*-Boc ketimine **3** provided only the AMBH product **9** (entry 6).

*N*-*tert*-Butylsulfonyl imine **1a** was selected for further optimization of the reaction conditions, as reported in Table 2. Starting our screening testing different catalysts, we observed that typical quinidine-derived catalysts **5b**–**f** were unable to promote the reaction, even after prolonged reaction times (entries 1–5). In all cases only degradation of the starting ketimine was observed. Instead, using β-isocupridine-based catalysts **5g**–**l**, the desired azetidine **6a** was obtained in all cases, with the exception of reaction with catalyst **5h** (entries 6–12).

The high conformational bias of β-ICD and its derivatives, as well as the presence of at least a hydrogen bond donor group at the C6 position of the quinoline ring, seem to be necessary requirements to coordinate the substrate and therefore to catalyse the reaction. Best results in terms of both er and chemical yield have been obtained employing catalyst **5g**, which was then used for a further screening of solvents and temperature.

Performing the reaction in apolar solvents such as dichloromethane and toluene, the desired product was observed just in very low yields (entries 13 and 14). Lowering the temperature to 0 °C no appreciable improvement of er was observed and prolonged reaction time was needed to obtain the desired azetidine in good yield (entry 15). At −20 °C the reaction was completely stopped (entry 16). Finally, on reducing the catalyst amount (entry 17) no improved er was observed, but just extension of the reaction time with a reduction of the chemical yield.

**Scheme 2:**
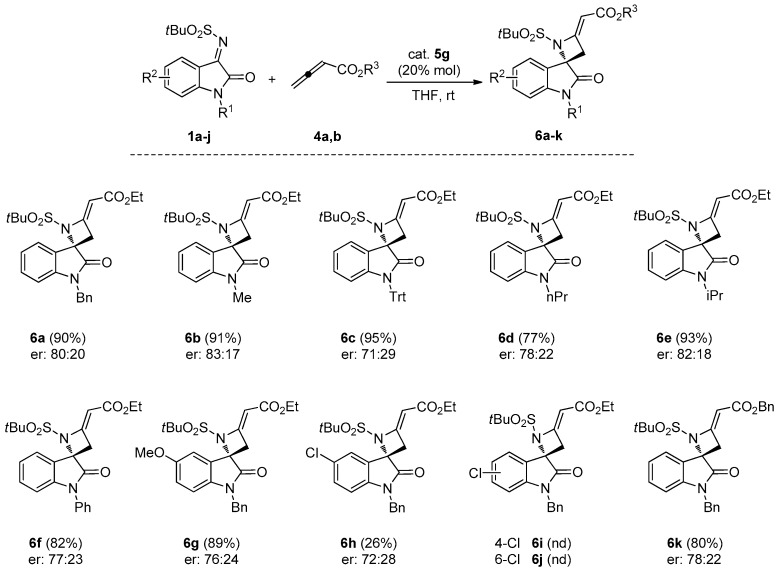
Substrate scope for the reaction of various ketimines **1a**–**j** with allenoates **4a**,**b**, catalyzed by catalyst **5g**. Reactions conditions: ketimine **1a**–**j** (0.15 mmol), **4a**,**b** (0.30 mmol), **5g** (0.03 mmol) in THF (1.5 mL). Isolated yields, after purification by column chromatography on silica gel, are reported. Er was determined by HPLC, on a chiral stationary phase. Trt = Trityl; nd = not detected.

With the best conditions in hand, we planned to screen *N*-*tert*-butylsulfonyl ketimines bearing different *N*-substituents at the oxindole ring (**1b**–**f**, Scheme 2). Such starting compounds have been prepared by *m*-chloroperbenzoic acid-mediated oxidation of the corresponding *N*-*tert*-butylsulfinyl ketimines and immediately used without purification (see Materials and Methods for the general synthetic procedure).

Generally, good yields and er were observed in the reaction with allenoate **4a**, under the catalysis of **5g**. The presence of the bulky trityl substituent at the oxindole nitrogen led to the corresponding spiroazetidine derivative **6c** with the best yield, but with the lowest enantioselectivity. The best result in terms of er was obtained performing the reaction with *N*-methyl substituted ketimine **1b**, leading to product **6b**.

Depending on whether electron-withdrawing or electron-donating group was introduced at 4-, 5- or 6-position of the benzene ring of starting ketimines (**1g**–**j**), the reactions proceeded in a different way. Good yield and enatioselectivity were observed for the synthesis of 5-OMe substituted spiroazetidine **6g**. In the presence of the electron withdrawing Cl-substituent at C5 position, a very low yield was achieved (**6h**). Finally, compounds **6i** and **6j** were not detected in the crude, but just degradation of the ketimine starting material was observed.

At the end, we investigated the formal [2 + 2] annulation with a different allenoate. Considering the possibility of a favourable π–π interaction with the aromatic portion of the catalyst, we selected benzyl buta-2,3-dienoate **4b**. Indeed, the corresponding azetidine **6k** could be easily obtained in good yield, even if in quite similar er.

**Scheme 3:**
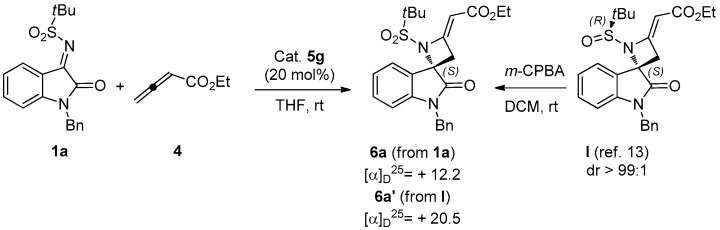
Chemical correlation for the assignment of absolute configuration of compound **6a**. For comparison of the chiral HPLC chromatograms of **6a** and **6a**′, see the Supporting Information.

The absolute configuration of the major enantiomer of compound **6a** was determined through chemical correlation, exploiting a proper conversion of reference compound **I** [13] (Scheme 3). More precisely, performing a selective sulfur oxidation, enantiopure compound **6a′** could be easily obtained from compound **I**. Relying on comparison of both optical rotation signs and chiral HPLC chromatograms, the (*S*)-configuration at the quaternary stereocenter of product **6a** could be assigned with certainty.

To rationalize the stereochemical outcome, we referred to the proposed mechanism for similar cinchona-base catalysed [2 + 2] annulation reactions [17]. Accordingly, the basic quinuclidine moiety acts as a nucleophile activator, producing a zwitterionic allylic carbanion from the allenoate reagent. The activated g-position of this intermediate undergoes addition to the ketimine, followed by an intramolecular attack back from ketimine’s nitrogen, leading to the azetidine ring and catalyst’s regeneration. From our experimental data, it results that the peculiar skeleton of β-ICD-type catalysts (e.g., **5g**), more rigid with respect to that of corresponding quinidine-catalysts (e.g., **5b**), seems the only able to bring reactants to the correct distance for a productive reaction.

**Scheme 4:**
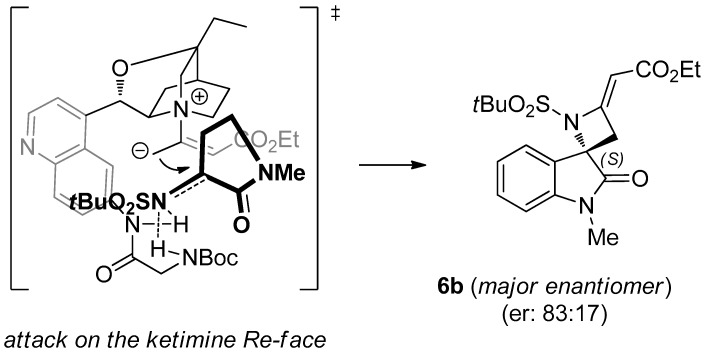
Possible transition state for reaction of ketimine **1b** with allenoate **4a**, under catalysis of **5g**. Ketimine **1b** is reported without the condensed phenyl ring for clarity.

Moreover, the presence of the *N*-Boc glycinamide unit at C6’ position in catalyst **5g** ensures a proper activation of the ketimine, through up to two hydrogen bonds. Relying on this disposition, to minimize the steric hindrance with the catalyst’s residue, ketimine offers its *Re*-face to the incoming γ-carbanion, leading to the corresponding spiroazetidine derivative in the prevailing (*S*)-configuration at the C3 of the oxindole ring. On this basis, a plausible transition state for the reported reaction is illustrated in Scheme 4, where ketimine **1b** is shown coordinated to the catalyst **5g** by double H-bonding, so explaining the found superiority of the amide N-H over the O-H group in this kind of process.

**Scheme 5:**
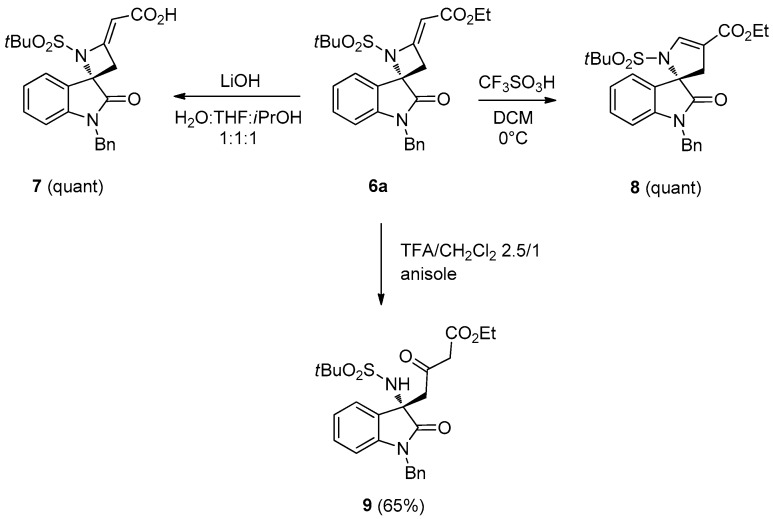
Reactions performed from compound **6a**.

To demonstrate the synthetic utility of obtained compounds, some reactions of **6a** have been performed (Scheme 5). Thus, acid **7** could be quantitatively obtained by reaction with LiOH. Aiming to selectively remove the *tert*-butylsulfonyl group, we refer to a milestone literature procedure [20], reporting the cleavage of such protecting groups to the parent amines by mild acidic solvolysis. Surprisingly, exposure of **6a** to 0.075 N TfOH/CH_2_Cl_2_ at 0 °C for two hours afforded quantitatively the rearranged product **8**, without trace of the expected spiroazetidine *N*-deprotected derivative. The spiropyrroline ring system of compound **8** was disclosed by careful analysis of mono- and bidimensional NMR spectra. In particular, the Heteronuclear Multiple Bond Correlation (HMBC) experiment allowed a complete and safe assignment of all ^1^H- and ^13^C-NMR signals. Changing the acid to trifluoroacetic acid (TFA) and adding anisole as scavenger, the reaction followed a different course, affording the unprecedented 3-*tert*-butylsulfonylamino, 3-substituted derivative **9**, as the result of the acid catalyzed azetidine ring opening. Evidently, the presence of the ethoxycarbonylmethylene substituent in compound **6a** diverts from the expected cleavage of the *tert*-butylsulfonyl group, addressing the reaction toward less predictable pathways. Anyway, preserving the integrity at the oxindole C3 stereogenic centre, compounds **8** and **9** can be considered relevant examples of almost unexplored spiro and 3,3-disubstituted oxindole derivatives.

## 3. Materials and Methods

### 3.1. General Experimental Procedures

All commercial materials (Aldrich, Fluka, St. Louis, MO, USA) were used without further purification. All solvents were of reagent grade or HPLC grade. All reactions were carried out under a nitrogen atmosphere unless otherwise noted. All reactions were monitored by thin layer chromatography (TLC) on precoated silica gel 60 F254; spots were visualized with UV light or by treatment with ninhydrin solution in ethanol. Products were purified by flash chromatography (FC) on silica gel 60 (230–400 mesh). ^1^H-NMR spectra and ^13^C-NMR spectra were recorded on 300 and 400 MHz spectrometers (AVANCE, Bruker, Billerica, MA, USA). Chemical shifts are reported in parts per million relative to the residual solvent. Multiplicities in ^1^H-NMR are reported as follows: s = singlet, d = doublet, t = triplet, m = multiplet, br s = broad singlet. ^13^C-NMR spectra have been recorded using the APT pulse sequence. The number of carbons reported in the ^13^C data are derived from Heteronuclear Multiple Bond Correlation (HMBC) experiments. High-resolution MS spectra were recorded with a Micromass Q-ToF micro TM mass spectrometer (Waters, Milford, MA, USA), equipped with an ESI source. Chiral HPLC analysis was performed on a PU-2080 system (**JASCO Europe**, Cremella (LC), Italy) (UV Detector and binary HPLC pump) at 254 nm. Chiralcel AD columns were purchased from Daicel Chemical Industries^®^ (Osaka, Japan). Optical rotator power ${\left[\alpha \right]}_{T}^{D}$ was measured with a Jasco P-1030 polarimeter, equipped with a cell of 1 dm path length and 1 mL capacity. The light used has a wavelength of 589 nm (sodium D line).

#### 3.1.1. General Procedure for the Synthesis of *N*-*tert*-Butylsulfonyl Ketimines **1a**–**j**

To a solution of *N*-substituted isatin (1.17 mmol, 1.0 eq) in anhydrous CH_2_Cl_2_ (2.9 mL, 0.4 M), Ti(O*i*Pr)_4_ (2.34 mmol, 2.0 eq) and 2-methyl-2-propanesulfinamide (1.4 mmol, 1.2 eq) were added. The solution was refluxed until complete disappearance of the starting materials (monitored by TLC). The reaction was quenched by adding saturated aq. NaHCO_3_ (15 mL) and diluted with CH_2_Cl_2_ (15 mL). The biphasic solution was filtered through a pad of Celite and the organic phase washed with water (2 × 15 mL), dried over Na_2_SO_4_ and the solvent was evaporated under reduced pressure. The crude was purified by flash chromatography (FC), using hexane/EtOAc/CH_2_Cl_2_ (gradient from 9:1:10 to 5/5/10) as eluent.

New compounds:

*(E)-N-(1-Propyl-2-oxoindolin-3-ylidene)-2-methylpropane-2-sulfinamide*. Prepared starting from *N*-1-propylisatin. Orange foam, yield: 82%. ^1^H-NMR (300 MHz, CDCl_3_) δ 8.37 (m, br, 1H), 7.41 (t, *J* = 7.8 Hz, 1H), 7.02 (t, *J* = 7.8 Hz, 1H), 6.79 (d, *J* = 7.8 Hz, 1H), 3.66 (t, *J* = 7.4 Hz, 2H), 1.70 (sext, *J* = 7.5 Hz, 2H), 1.38 (s, 9H), 0.96 (t, *J* = 7.4 Hz, 3H); ^13^C-NMR (101 MHz, CDCl_3_) δ 161.0, 148.9, 144.2, 136.1, 124.9, 123.9 (2C), 109.9, 54.1, 42.5, 23.8 (3C), 21.3, 12.0; HRMS (ESI): [M + Na]^+^, Calcd. for C_15_H_20_N_2_NaO_2_S^+^ 315.1138, found 315.1142.

*(E)-N-(1-Isopropyl-2-oxoindolin-3-ylidene)-2-methylpropane-2-sulfinamide*. Prepared starting from *N*-isopropylisatin. Orange foam, yield: 88%. ^1^H-NMR (300 MHz, CDCl_3_) δ 8.40 (m, br, 1H), 7.41 (t, *J* = 7.8 Hz, 1H), 7.03 (t, *J* = 7.8 Hz, 1H), 6.93 (d, *J* = 7.7 Hz, 1H), 4.50 (hept, *J* = 6.8 Hz, 1H), 1.50 (d, *J* = 6.8 Hz, 6H), 1.39 (s, 9H); ^13^C-NMR (101 MHz, CDCl_3_) δ 162.3, 148.3, 143.9, 136.0, 123.8, 123.6 (2C), 110.9, 55.9, 45.4, 23.7 (3C), 22.8 (2C); HRMS (ESI): [M + Na]^+^, Calcd. for C_15_H_20_N_2_NaO_2_S^+^ 315.1138, found 315.1147.

*(E)-2-Methyl-N-(2-oxo-1-phenylindolin-3-ylidene)propane-2-sulfinamide*. Prepared starting from *N*-phenylisatin. Orange foam, yield: 76%. ^1^H-NMR (300 MHz, CDCl_3_) δ 8.46 (m, br, 1H), 7.54 (t, *J* = 7.6 Hz, 2H), 7.49–7.33 (m, 4H), 7.10 (t, *J* = 7.8 Hz, 1H), 6.80 (d, *J* = 7.8 Hz, 1H), 1.43 (s, 9H); ^13^C-NMR (101 MHz, CDCl3) δ 158.2, 152.3, 149.4, 136.1, 133.9, 130.5 (3C), 129.3 (2C), 127.0, 124.9, 124.7, 111.1, 55.9, 23.9 (3C); HRMS (ESI): [M + Na]^+^, Calcd. for C_18_H_18_N_2_NaO_2_S^+^ 349.0981, found 349.0989.

To a solution of the desired sulfinamide in CH_2_Cl_2_ (6 mL), *m*-CPBA (1.5 eq) was slowly added at room temperature and the mixture was stirred until completion of the reaction (monitored by TLC). The reaction was quenched by adding saturated aq. NaHCO_3_ (15 mL) and diluted with CH_2_Cl_2_ (15 mL). The organic phase was washed with sat. NaHCO_3_ (2 × 40 mL), dried (Na_2_SO_4_), and concentrated. The corresponding sulfonamide was used without further purification.

New compounds:

*(E)-2-Methyl-N-(2-oxo-1-tritylindolin-3-ylidene)propane-2-sulfonamide* (**1c**). Red foamy solid, yield: 97%. ^1^H-NMR (300 MHz, CDCl_3_) δ 8.42 (d, *J* = 7.7 Hz, 1H), 7.49–7.37 (m, 6H), 7.37–7.19 (m, 9H), 7.11 (t, *J* = 7.8 Hz, 1H), 6.97 (d, *J* = 7.8 Hz, 1H), 6.35 (d, *J* = 7.8 Hz, 1H), 1.44 (s, 9H); ^13^C-NMR (101 MHz, CDCl_3_) δ 162.1, 151.5, 144.5, 141.7 (3C), 136.9, 130.1 (6C), 128.6 (6C), 128.0 (3C), 123.9, 123.8, 117.9, 114.9, 75.9, 60.7, 24.5 (3C); HRMS (ESI): [M + Na]^+^, Calcd. for C_31_H_28_N_2_NaO_3_S^+^ 531.1716, found 531.1709.

*(E)-2-Methyl-N-(2-oxo-1-propylindolin-3-ylidene)propane-2-sulfonamide* (**1d**). Red foamy solid, yield: 95%. ^1^H-NMR (300 MHz, CDCl_3_, 7:3 mixture of two rotamers) δ 8.40 (m, br, 0.3H), 8.06 (d, *J* = 7.8 Hz, 0.7H), 7.50 (t, *J* = 7.8 Hz, 0.3H), 7.45 (t, *J* = 7.8 Hz, 0.7H), 7.11 (t, *J* = 7.8 Hz, 0.7H), 7.07 (t, *J* = 7.8 Hz, 0.3H), 6.93 (d, *J* = 7.8 Hz, 0.7H), 6.82 (t, *J* = 7.8 Hz, 0.3H), 3.74–3.62 (m, 2H), 1.74 (sext, br, *J* = 6.8 Hz, 2H), 1.62 (s, 6.3H), 1.60 (s, 2.7H), 0.99 (t, br, *J* = 6.8 Hz, 3H); ^13^C-NMR (75 MHz, CDCl_3_) δ 162.6, 150.4, 147.4, 139.0 and 138.5 (1C), 124.0 (2C), 116.2, 110.4 and 110.3 (1C), 60.6, 43.1 and 42.7 (1C), 24.5 (3C), 21.2 and 20.8 (1C), 12.0; HRMS (ESI): [M + Na]^+^, Calcd. for C_15_H_20_N_2_NaO_3_S^+^ 331.1087, found 331.1096.

*(E)-2-(1-Isopropyl-2-oxoindolin-3-ylidene)-2-methylpropane-2-sulfonamide* (**1e**). Red foamy solid, yield: 99%. ^1^H-NMR (400 MHz, CDCl_3_, 17:3 mixture of two rotamers) δ 8.19 (dd, *J* = 7.8 and 1.7 Hz, 0.15H), 8.12 (dd, *J* = 7.8 and 1.7 Hz, 0.85H), 7.76 (ddd, *J* = 8.2, 7.5 and 1.7 Hz, 0.15H), 7.73 (ddd, *J* = 8.2, 7.5 and 1.7 Hz, 0.85H), 7.34–7.21 (m, 2H), 4.77 (hept, *J* = 6.8 Hz, 1H), 1.62 (d, *J* = 6.9 Hz, 6H), 1.62 (s, 1.35H) 1.57 (s, 7.65H); ^13^C-NMR (101 MHz, CDCl_3_) δ 162.6, 158.0, 149.9, 138.9 and 138.5 (1C), 124.9 and 124.7 (1C), 124.0 and 123.7 (1C), 113.3, 112.0 and 111.3 (1C), 60.0, 45.7 and 45.5 (1C), 24.7 and 24.5 (3C), 19.9 (2C); HRMS (ESI): [M + Na]^+^, Calcd. for C_15_H_20_N_2_NaO_3_S^+^ 331.1087, found 331.1081.

*(E)-2-Methyl-N-(2-oxo-1-phenylindolin-3-ylidene)propane-2-sulfonamide* (**1f**). Red foamy solid, yield: 98%. ^1^H-NMR (400 MHz, CDCl_3_, 7:3 mixture of two rotamers) δ 8.21 (dd, *J* = 7.9 and 1.4 Hz, 0.7H), 8.00 (d, br, *J* = 7.9 Hz, 0.3H), 7.69–7.53 (m, 4H), 7.44 (t, *J* = 7.8 Hz, 0.3H), 7.41–7.26 (m, 2.7H), 6.58 (d, *J* = 7.8 Hz, 1H), 1.47 (s, 9H); ^13^C-NMR (101 MHz, CDCl_3_) δ 162.2, 150.9, 143.1, 138.5 and 137.5 (1C), 133.7, 130.6 (3C), 129.6 and 129.5 (2C), 127.1, 125.0 and 124.7 (1C), 115.9, 111.4, 60.8, 24.9–24.5 (3C); HRMS (ESI): [M + Na]^+^, Calcd. for C_18_H_18_N_2_NaO_3_S^+^ 365.0930, found 365.0939.

*(E)-N-(1-Benzyl-5-methoxy-2-oxoindolin-3-ylidene)-2-methylpropane-2-sulfonamide* (**1g**). Red foamy solid, yield: 98%. ^1^H-NMR (400 MHz, CDCl_3_) δ 7.42–7.26 (m, 6H), 6.99 (dd, *J* = 8.2 and 2.6 Hz, 1H), 6.62 (d, *J* = 8.3 Hz, 1H), 4.89 (s, 2H), 3.79 (s, 3H), 1.64 (s, 9H); ^13^C-NMR (101 MHz, CDCl_3_) δ 162.7, 156.7, 145.2, 144.1, 135.3, 129.7 (2C), 128.8, 128.0 (2C), 126.7, 125.6, 116.7, 111.9, 60.7, 56.7, 44.9, 24.6 (3C); HRMS (ESI): [M + Na]^+^, Calcd. for C_20_H_22_N_2_NaO_4_S^+^ 409.1192, found 409.1186.

*(E)-N-(1-Benzyl-5-chloro-2-oxoindolin-3-ylidene)-2-methylpropane-2-sulfonamide* (**1h**). Red foamy solid, yield: 92%. ^1^H-NMR (400 MHz, CDCl_3_, 4:1 mixture of two rotamers) δ 7.42–7.26 (m, 7H), 6.76 (d, *J* = 8.2 Hz, 0.2H), 6.68 (d, *J* = 8.2 Hz, 0.8H), 4.93 (s, 2H), 1.67 (s, 1.8H), 1.65 (s, 7.2H); ^13^C-NMR (101 MHz, CDCl_3_) δ 161.2, 148.2, 145.6, 138.3 and 137.9 (1C), 134.7, 130.7 and 129.8 (2C), 129.9, 129.0 (2C), 128.0 (2C), 116.9, 112.2, 63.9, 45.5 and 45.0 (1C), 24.6 and 24.5 (3C); HRMS (ESI): [M + Na]^+^, Calcd. for C_19_H_19_ClN_2_NaO_3_S^+^ 413.0697, found 413.0674.

#### 3.1.2. General Procedure for the Synthesis of Spiroazetidines **6a**–**h**, **6k**

To a solution of the appropriate *N*-*tert*-butylsulfonyl ketimine **1a**–**j** (0.10 mmol) and catalyst **5g** (0.02 mmol) in THF (1.5 mL), allenoate **4a** (or **4b**, for spiroazetidine **6k**) was added (0.20 mmol). The mixture was stirred at room temperature and the conversion was monitored by TLC. The solvent was evaporated under reduced pressure and the crude was purified by FC, using CH_2_Cl_2_/EtOAc 9/1 as eluent.

*(S,E)-Ethyl-2-(1′-benzyl-1-(tert-butylsulfonyl)-2′-oxospiro[azetidine-2,3′-indolin]-4-ylidene)acetate* (**6a**). Pale orange foam; yield: 90%. ^1^H-NMR (300 MHz, CDCl_3_) δ 7.50 (d, br, *J* = 7.8 Hz, 1H), 7.19–7.38 (m, 6H), 7.08 (t, br, *J* = 7.7 Hz, 1H), 6.69 (d, br, *J* = 7.8 Hz, 1H), 5.68 (s, br, 1H), 5.04 and 4.83 (AB system, *J* = 15.6 Hz, 2H), 4.16 (q, *J* = 7.1 Hz, 2H), 3.71 (dd, *J* = 15.6 and 1.9 Hz, 1H), 3.44 (dd, *J* = 15.6 and 1.9 Hz, 1H), 1.33 (s, 9H), 1.27 (t, *J* = 7.1 Hz, 3H); ^13^C-NMR (101 MHz, CDCl_3_) δ 173.3, 167.3, 159.2, 143.6, 134.6, 129.0 (3C), 128.2, 127.5 (2C), 126.4, 126.2, 124.6, 113.2, 94.3, 70.6, 59.7, 58.9, 44.4, 41.8, 22.7 (3C), 14.5; ${\left[\alpha \right]}_{\mathrm{25}}^{D}$ = +12.2 (*c* 0.90, CHCl_3_); HRMS (ESI): [M + Na]^+^, Calcd. for C_25_H_28_N_2_NaO_5_S^+^ 491.1611, found 491.1604; enantiomeric ratio: 80:20, determined by HPLC (C-AD, Hexane/iPrOH 7:3, flow: 0.7 mL/min, λ = 254 nm): *t*_R_ = 16.80 min (major) *t*_R_ = 23.38 min (minor).

*(S,E)-Ethyl-2-(1-(tert-butylsulfonyl)-1′-methyl-2′-oxospiro[azetidine-2,3′-indolin]-4-ylidene)acetate* (**6b**). Pale orange foam; yield: 91%. ^1^H-NMR (400 MHz, CDCl_3_) δ 7.50 (d, *J* = 7.5 Hz, 1H), 7.38 (d, *J* = 7.5 Hz, 1H), 7.12 (t, *J* = 7.5 Hz, 1H), 6.86 (d, *J* = 7.5 Hz, 1H), 5.66 (t, *J* = 1.9 Hz, 1H), 4.16 (q, *J* = 7.1 Hz, 2H), 3.66 (dd, *J* = 16.0, 1.9 Hz, 1H), 3.41 (dd, *J* = 16.0, 1.9 Hz, 1H), 3.24 (s, 3H), 1.32 (s, 9H), 1.23–1.30 (m, 3H); ^13^C-NMR (101 MHz, CDCl_3_) δ 173.3, 167.7, 159.4, 144.7, 131.8, 125.9, 125.0, 123.7, 109.5, 95.9, 71.7, 62.2, 60.5, 39.8, 27.3, 24.5 (3C), 15.0; ${\left[\alpha \right]}_{\mathrm{25}}^{D}$ = +19.3 (*c* 0.80, CHCl_3_); HRMS (ESI): [M + Na]^+^, Calcd. for C_19_H_24_N_2_NaO_5_S^+^: 415.1298, found 415.1303; enantiomeric ratio: 83:17, determined by HPLC (C-AD, Hexane/iPrOH 7:3, flow: 0.7 mL/min, λ = 254 nm): *t*_R_ = 24.96 min (major) *t*_R_ = 29.58 min (minor).

*(S,E)-Ethyl-2-(1-(tert-butylsulfonyl)-2′-oxo-1′-tritylspiro[azetidine-2,3′-indolin]-4-ylidene)acetate* (**6c**). Pale orange foam; yield: 95%. ^1^H-NMR (300 MHz, CDCl_3_) δ 7.50–7.39 (m, 7H), 7.30–7.16 (m, 9H), 7.03–6.89 (m, 2H), 6.28 (d, *J* = 7.4 Hz, 1H), 5.62 (t, *J* = 1.9 Hz, 1H), 4.19–4.05 (m, 2H), 3.58 (dd, *J* = 15.7, 1.9 Hz, 1H), 3.31 (dd, *J* = 15.7, 1.9 Hz, 1H), 1.34 (s, 9H), 1.24 (t, *J* = 6.8 Hz, 3H); ^13^C-NMR (75 MHz, CDCl_3_) δ 172.9, 167.2, 9.0, 144.0, 141.7 (3C), 129.5 (7C), 127.7 (6C), 127.1 (3C), 125.6, 123.9, 122.6, 116.6, 94.9, 74.9, 71.6, 61.7, 59.8, 40.1, 24.1 (3C), 14.4; ${\left[\alpha \right]}_{\mathrm{25}}^{D}$ = −0.67 (*c* 1.5, CHCl_3_); HRMS (ESI): [M + Na]^+^, Calcd. for C_37_H_36_N_2_NaO_5_S^+^: 643.2237, found 643.2240; enantiomeric ratio: 71:29, determined by HPLC (C-AD, Hexane/iPrOH 7:3, flow: 0.7 mL/min, λ = 254 nm): *t*_R_ = 8.00 min (major) *t*_R_ = 11.84 min (minor).

*(S,E)-Ethyl-2-(1-(tert-butylsulfonyl)-2′-oxo-1′-propylspiro[azetidine-2,3′-indolin]-4-ylidene)acetate* (**6d**). Pale orange foam; yield: 77%. ^1^H-NMR (300 MHz, CDCl_3_) δ 7.49 (d, *J* = 7.4 Hz, 1H), 7.35 (td, *J* = 7.4 and 1.1 Hz, 1H), 7.10 (t, *J* = 7.4 Hz, 1H), 6.86 (d, *J* = 7.4 Hz, 1H), 5.65 (t, *J* = 1.9 Hz, 1H), 4.15 (q, *J* = 7.1 Hz, 2H), 3.75 (dt, *J* = 14.6 and 6.8 Hz, 1H), 3.63 (dd, *J* = 15.6 and 1.9 Hz, 1H), 3.58 (dt, *J* = 14.6 and 6.8 Hz, 1H), 3.39 (dd, *J* = 15.6 and 1.9 Hz, 1H), 1.72 (sext, *J* = 6.8 Hz, 2H), 1.32 (s, 9H), 1.27–1.22 (m, 3H) 0.97 (t, *J* = 6.8 Hz, 3H); ^13^C-NMR (101 MHz, CDCl_3_) δ 173.1, 167.8, 159.6, 142.9, 131.6, 126.2, 125.1, 123.5, 109.8, 95.7, 71.8, 60.5, 42.7, 40.0, 30.4, 24.6 (3C), 21.2, 15.1, 12.0; ${\left[\alpha \right]}_{\mathrm{25}}^{D}$ = +5.5 (*c* 1.02, CHCl_3_); HRMS (ESI) calculated for C_21_H_28_N_2_NaO_5_S^+^ [M + Na]^+^ 443.1611, found 443.1617; enantiomeric ratio: 78:22, determined by HPLC (C-AD, Hexane/iPrOH 7:3, flow: 0.7 mL/min, λ = 254 nm): *t*_R_ = 7.73 min (major) *t*_R_ = 9.85 min (minor).

*(S,E)-Ethyl-2-(1-(tert-butylsulfonyl)-1′-isopropyl-2′-oxospiro[azetidine-2,3′-indolin]-4-ylidene)acetate* (**6e**). Pale orange foam; yield: 93%. ^1^H-NMR (300 MHz, CDCl_3_) δ 7.49 (d, *J* = 7.7 Hz, 1H), 7.34 (t, *J* = 7.7 Hz, 1H), 7.08 (t, *J* = 7.7 Hz, 1H), 6.99 (d, *J* = 7.7 Hz, 1H), 5.64 (t, *J* = 2.0 Hz, 1H), 4.56 (hept, *J* = 6.8 Hz, 1H), 4.15 (q, *J* = 7.6 Hz, 2H), 3.64 (dd, *J* = 15.9 and 2.0 Hz, 1H), 3.40 (dd, *J* = 15.9, 2.0 Hz, 1H), 1.50 (d, *J* = 6.8 Hz, 3H), 1.48 (d, *J* = 6.8 Hz, 3H), 1.30 (s, 9H), 1.26 (t, *J* = 7.6 Hz, 3H); ^13^C-NMR (101 MHz, CDCl_3_) δ 172.4, 167.2, 159.1, 143.2, 130.9, 125.6, 124.8, 122.5, 110.4, 95.0, 71.1, 61.5, 59.9, 44.7, 39.1, 23.9 (3C), 19.2 (2C), 14.5; ${\left[\alpha \right]}_{\mathrm{25}}^{D}$ = +14.7 (*c* 0.99, CHCl_3_); HRMS (ESI) calculated for C_21_H_28_N_2_NaO_5_S^+^ [M + Na]^+^ 443.1611, found 443.1616; enantiomeric ratio 82:18, determined by HPLC (C-AD, Hexane/iPrOH 7:3, flow: 0.7 mL/min, λ = 254 nm): *t*_R_ = 7.32 min (major) *t*_R_ = 9.33 min (minor).

*(S,E)-Ethyl-2-(1-(tert-butylsulfonyl)-2′-oxo-1′-phenylspiro[azetidine-2,3′-indolin]-4-ylidene)acetate* (**6f**). Pale orange foam; yield: 82%. ^1^H-NMR (300 MHz, CDCl_3_) δ 7.64–7.39 (m, 6H), 7.30 (t, *J* = 7.8 Hz, 1H), 7.15 (t, *J* = 7.8 Hz, 1H), 6.83 (d, *J* = 7.8 Hz, 1H), 5.68 (t, *J* = 2.0 Hz, 1H), 4.17 (q, *J* = 7.2 Hz, 2H), 3.77 (dd, *J* = 16.0, 2.0 Hz, 1H), 3.52 (dd, *J* = 16.0, 2.0 Hz, 1H), 1.33 (s, 9H), 1.27 (t, *J* = 7.2 Hz, 3H); ^13^C-NMR (101 MHz, CDCl_3_) δ 172.3, 167.2, 158.9, 144.6, 134.0, 131.2, 129.8 (2C), 128.5, 126.6 (2C), 125.8, 124.9, 123.5, 110.2, 95.4, 71.3, 61.6, 60.0, 39.2, 24.0 (3C), 14.5; ${\left[\alpha \right]}_{\mathrm{25}}^{D}$ = +10.3 (c 1.04, CHCl_3_); HRMS (ESI) calculated for C_24_H_26_N_2_NaO_5_S^+^ [M + Na]^+^ 477.1455, found 447.1460; enantiomeric ratio: 77:23, determined by HPLC (C-AD, Hexane/iPrOH 4:1, flow: 0.7 mL/min, λ = 254 nm): *t*_R_ = 9.63 min (major) *t*_R_ = 14.79 min (minor).

*(S,E)-Ethyl-2-(1′-benzyl-1-(tert-butylsulfonyl)-5′-methoxy-2′-oxospiro[azetidine-2,3′-indolin]-4-ylidene)-acetate* (**6g**). Pale orange foam; yield: 89%. ^1^H-NMR (400 MHz, CDCl_3_) δ 7.38–7.27 (m, 5H), 7.12 (d, *J* = 2.7 Hz, 1H), 6.78 (dd, *J* = 8.4 and 2.7 Hz, 1H), 6.60 (d, *J* = 8.4 Hz, 1H), 5.71 (t, *J* = 1.9 Hz, 1H), 5.05 and 4.81 (AB system, *J* = 15.8 Hz, 2H), 4.19 (q, *J* = 7.2 Hz, 2H), 3.78 (s, 3H), 3.75 (dd, *J* = 15.9 and 1.9 Hz, 1H), 3.45 (dd, *J* = 15.9 and 1.9 Hz, 1H), 1.38 (s, 9H), 1.30 (t, *J* = 7.2 Hz, 3H); ^13^C-NMR (101 MHz, CDCl_3_) δ 172.5, 167.1, 158.8, 156.4, 136.4, 135.2, 128.8 (2C), 127.7, 127.3 (2C), 127.0, 115.4, 111.4, 110.5, 95.2, 71.5, 61.8, 59.9, 55.9, 44.3, 39.7, 24.0 (3C), 14.4; ${\left[\alpha \right]}_{\mathrm{25}}^{D}$ = +8.9 (*c* 0.95, CHCl_3_); HRMS (ESI) calculated for C_26_H_30_N_2_NaO_5_S^+^ [M + Na]^+^ 521.1717, found 521.1720; enantiomeric ratio: 76:24, determined by HPLC (C-AD, Hexane/iPrOH 4:1, flow: 0.7 mL/min, λ = 254 nm): *t*_R_ = 15.00 min (major) *t*_R_ = 20.63 min (minor).

*(S,E)-Ethyl-2-(1′-benzyl-1-(tert-butylsulfonyl)-5′-chloro-2′-oxospiro[azetidine-2,3′-indolin]-4-ylidene)-acetate* (**6h**). Pale orange foam; yield: 26%. ^1^H-NMR (400 MHz, CDCl_3_) δ 7.52 (d, *J* = 2.1 Hz, 1H), 7.38–7.26 (m, 5H), 7.23 (dd, *J* = 8.2 and 2.1 Hz, 1H), 6.63 (d, *J* = 8.2 Hz, 1H), 5.71 (t, *J* = 2.1 Hz, 1H), 5.07 and 4.82 (AB system, *J* = 16.0 Hz, 2H), 4.20 (q, *J* = 7.1 Hz, 2H), 3.74 (dd, *J* = 15.9 and 2.1 Hz, 1H), 3.47 (dd, *J* = 15.9 and 2.1 Hz, 1H), 1.39 (s, 9H), 1.31 (t, *J* = 7.1 Hz, 3H); ^13^C-NMR (101 MHz, CDCl_3_) δ 172.4, 166.9, 158.2, 141.6, 136.7, 134.6, 130.8, 129.0 (2C), 128.7, 127.9, 127.2 (2C), 124.7, 111.0, 95.6, 70.7, 61.8, 60.0, 44.4, 39.5, 23.9 (3C), 14.4; ${\left[\alpha \right]}_{\mathrm{25}}^{D}$ = +9.8 (*c* 1.05, CHCl_3_); HRMS (ESI) calculated for C_25_H_27_ClN_2_NaO_5_S^+^ [M + Na]^+^ 525.1221, found 525.1229; enantiomeric ratio: 72:28, determined by HPLC (C-AD, Hexane/iPrOH 4:1, flow: 0.7 mL/min, λ = 254 nm): *t*_R_ = 10.31 min (major) *t*_R_ = 16.59 (minor).

*(S,E)-Benzyl-2-(1′-benzyl-1-(tert-butylsulfonyl)-2′-oxospiro[azetidine-2,3′-indolin]-4-ylidene)acetate* (**6k**). Pale yellow foam; yield: 80%. ^1^H-NMR (400 MHz, CDCl_3_) δ 7.53 (d, br, *J* = 7.8 Hz, 1H), 7.44–7.23 (m, 11H), 7.11 (t, br, *J* = 7.7 Hz, 1H), 6.72 (d, br, *J* = 7.8 Hz, 1H), 5.78 (s, br, 1H), 5.19 (s, br, 2H), 5.08 and 4.84 (AB system, *J* = 15.8 Hz, 2H), 3.74 (d, br, *J* = 16.0 Hz, 1H), 3.47 (d, br, *J* = 16.0 Hz, 1H), 1.36 (s, 9H); ^13^C-NMR (101 MHz, CDCl_3_) δ 173.4, 167.5, 160.0, 143.9, 136.9, 135.7, 131.7, 129.5 (3C), 129.3, 129.0, 128.8, 128.4, 128.0 (3C), 126.0, 125.1, 123.8, 110.7, 95.5, 71.8, 66.6, 62.3, 44.9, 40.3, 24.6 (3C); ${\left[\alpha \right]}_{\mathrm{25}}^{D}$ = +15.0 (*c* 0.8, CHCl_3_); HRMS (ESI): [M + Na]^+^, Calcd. for C_30_H_30_N_2_NaO_5_S^+^ 553.1768, found 553.1772; enantiomeric ratio: 78:22, determined by HPLC (C-AD, Hexane/iPrOH 7:3, flow: 0.7 mL/min, λ = 254 nm): *t*_R_ = 16.80 min (major) *t*_R_ = 23.38 min (minor).

#### 3.1.3. Reactions of **6a**

*(S,E)-2-(1′-Benzyl-1-(tert-butylsulfonyl)-2′-oxospiro[azetidine-2,3′-indolin]-4-ylidene)acetic acid* (**7**). To a solution of **6a** (0.21 mmol) in water/THF/iPrOH (1:1:1, 1.5 mL), LiOH (0.63 mmol) was added and the mixture was stirred at room temperature. After the completion of the reaction (monitored by TLC), the reaction mixture was quenched with a 1 M aqueous solution of HCl and extracted with CH_2_Cl_2_. The organic layer was collected, dried over anhydrous sodium sulfate, and concentrated under reduced pressure affording the product as a white foam (quantitative yield). ^1^H-NMR (400 MHz, CDCl_3_) δ 7.54 (d, *J* = 7.4 Hz, 1H), 7.41–7.23 (m, 6H), 7.12 (t, *J* = 7.4 Hz, 1H), 6.73 (d, *J* = 7.4 Hz, 1H), 5.71 (t, *J* = 1.9 Hz, 1H), 5.07 and 4.87 (AB system, *J* = 15.8 Hz, 2H), 3.76 (dd, *J* = 16.3 and 2.0 Hz, 1H), 3.49 (dd, *J* = 16.3 and 2.0 Hz, 1H), 2.05 (m, br, 1H), 1.37 (s, 9H); ^13^C-NMR (101 MHz, CDCl_3_) δ 172.6, 172.5, 161.3, 143.2, 135.0, 131.1, 128.9 (2C), 127.8, 127.3 (2C), 125.1, 124.4, 123.2, 110.1, 94.4, 71.2, 61.8, 44.3, 39.7, 23.9 (3C); ${\left[\alpha \right]}_{\mathrm{25}}^{D}$ = +5.7 (*c* 0.79, CHCl_3_); HRMS (ESI) calculated for C_23_H_24_N_2_NaO_5_S^+^ [M + Na]^+^ 463.1298, found 463.1289.

*(S)-Ethyl-1-benzyl-1′-(tert-butylsulfonyl)-2-oxo-1′,3′-dihydrospiro[indoline-3,2′-pyrrole]-4′-carboxylate* (**8**). To a stirred solution of **6a** (0.21 mmol) in CH_2_Cl_2_ (9 mL) at 0 °C, CF_3_SO_3_H (0.68 mmol) was added. After stirring at 0 °C for 2 h, the mixture was neutralized with 0.1 M NaOH and extracted with CH_2_Cl_2_ (4 × 15 mL). The combined organic phases were dried (MgSO_4_) and concentrated in vacuo, affording the product as a white foam (quantitative yield). ^1^H-NMR (400 MHz, CDCl_3_) δ 7.56 (d, br, *J* = 7.8 Hz, 1H), 7.40–7.25 (m, 6H), 7.05 (t, br, *J* = 7.7 Hz, 1H), 6.79 (d, br, *J* = 7.8 Hz, 1H), 6.15 (s, br, 1H), 4.97 and 4.91 (AB system, *J* = 15.5 Hz, 2H), 4.23 (q, *J* = 7.1 Hz, 2H), 3.49 (d, br, *J* = 16.4 Hz, 1H), 2.63 (d, br, *J* = 16.4 Hz, 1H), 1.46 (s, 9H), 1.38 (t, *J* = 7.1 Hz, 3H); ^13^C-NMR (101 MHz, CDCl_3_) δ 171.1, 170.5, 169.7, 142.3, 134.7, 131.6, 129.1 (2C), 128.1, 127.3 (2C), 126.4, 125.1, 124.0, 81.2, 79.7, 66.6, 58.5, 44.2, 38.4, 24.0 (3C), 14.2; ${\left[\alpha \right]}_{\mathrm{25}}^{D}$ = +6.3 (*c* 0.53, CHCl_3_); HRMS (ESI): [M + Na]^+^, Calcd. for C_25_H_28_N_2_NaO_5_S^+^ 491.1611, found 491.1617.

*(S)-Ethyl-4-(1-benzyl-3-(tert-butylsulfonamido)-2-oxoindolin-3-yl)-3-oxobutanoate* (**9**). To a solution of **6a** (0.20 mmol) in trifluoroacetic acid (5 mL) and CH_2_Cl_2_ (2 mL), anisole (4 mmol) was added, and the mixture was stirred for 1 h at 60 °C. To this saturated Na_2_CO_3_ was added and organic materials were extracted with EtOAc. Dried and concentrated extract was subjected to FC (Hexane/EtOAc 1/1) to give the product as a pale yellow foam (yield: 65%). ^1^H-NMR (400 MHz, CDCl_3_) δ 7.57 (d, br, *J* = 7.8 Hz, 1H), 7.43–7.24 (m, 5H), 7.21 (t, br, *J* = 7.7 Hz, 1H), 7.04 (t, *J* = 7.7 Hz, 1H), 6.72 (d, br, *J* = 7.8 Hz, 1H), 5.75 (s, br, 1H), 5.00 and 4.95 (AB system, *J* = 16.0 Hz, 2H), 4.15 (q, *J* = 7.1 Hz, 2H), 3.60 (d, *J* = 17.4 Hz, 1H), 3.45 and 3.40 (AB system, *J* = 16.0 Hz, 2H), 3.07 (d, *J* = 17.4 Hz, 1H), 1.34 (s, 9H), 1.22 (t, *J* = 7.1 Hz, 3H); ^13^C-NMR (101 MHz, CDCl_3_) δ 201.0, 174.7, 166.1, 142.4, 135.6, 129.7, 128.8 (2C), 128.7, 127.7, 127.3 (2C), 125.3, 122.9, 109.7, 61.7, 60.6, 60.3, 50.1, 49.2, 44.4, 24.1 (3C), 14.0; ${\left[\alpha \right]}_{\mathrm{25}}^{D}$ = −57.4 (*c* 0.50, CHCl_3_); HRMS (ESI): [M + Na]^+^, Calcd. for C_25_H_30_N_2_NaO_6_S^+^ 509.1717, found 509.1711.

## Figures and Tables

**Table 1:**
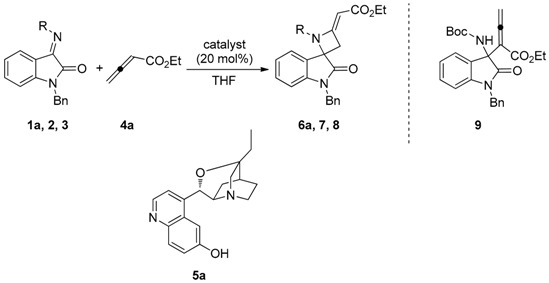
Variation of the isatin-derived ketimines. *^a^*

Entry	Ketimine (R)	Catalyst	Product (Yield %) *^b^*	er *^c^*
1	**1a** (SO_2_*t*Bu)	**DABCO**	**6a** (90)	-
2	**2** (Ts)	**DABCO**	**7** (77)	-
3	**3** (Boc)	**DABCO**	**8** (48), **9** (28)	-
4	**1a** (SO_2_*t*Bu)	**5a**	**6a** (91)	70:30
5	**2** (Ts)	**5a**	**7** (80)	61:39
6	**3** (Boc)	**5a**	**9** (52)	-

*^a^* Reaction conditions: ketimine (0.15 mmol), allenoate **4a** (0.30 mmol) and catalyst (0.030 mmol) in THF (1.5 mL) at room temperature; *^b^* Isolated yields, after purification by column chromatography on silica gel; *^c^* Determined by HPLC, on a chiral stationary phase.

**Table 2:**
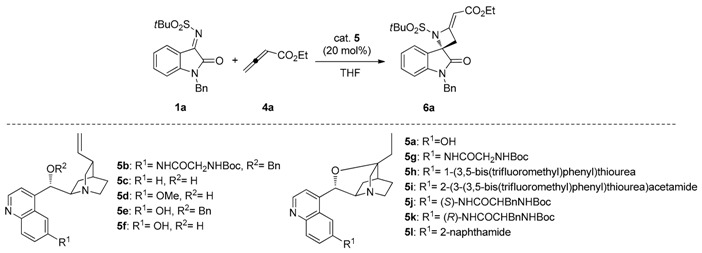
Catalyst and solvent screen for the reaction of *N*-*tert*-butylsulfonyl imine **1a** with ethyl buta-2,3-dienoate (**4a**). *^a^*

Entry	Solvent	Catalyst	Time (h)	Yield (%) *^b^*	er *^c^*
1	THF	**5b**	48	nd	-
2	THF	**5c**	48	nd	-
3	THF	**5d**	48	nd	-
4	THF	**5e**	48	nd	-
5	THF	**5f**	48	nd	-
6	THF	**5a**	1	91	70:30
7	THF	**5g**	3	90	80:20
8	THF	**5h**	48	nd	-
9	THF	**5i**	3	64	74:26
10	THF	**5j**	3	87	70:30
11	THF	**5k**	3	86	77:23
12	THF	**5l**	3	83	70:30
13	CH_2_Cl_2_	**5g**	24	12	-
14	Toluene	**5g**	24	16	-
15 *^d^*	THF	**5g**	24	78	80:20
16 *^e^*	THF	**5g**	48	nd	-
17 *^f^*	THF	**5g**	24	68	80:20

*^a^* Reaction conditions: ketimine **1a** (0.15 mmol), **4a** (0.30 mmol) and catalyst (0.03 mmol) in solvent (1.5 mL) at room temperature, unless otherwise noted; *^b^* Isolated yields, after purification by column chromatography on silica gel; *^c^* Determined by HPLC, on a chiral stationary phase; *^d^* Reaction performed at 0 °C; *^e^* Reaction performed at −20 °C; *^f^* Catalyst loading 10% mol. nd = not detected.

## Supplementary Materials

The following are available online, NMR spectra of all new compounds and HPLC chromatograms of spiroazetidines **6a**–**k**.
