# Supplementary material for: Organocatalytic Access to Enantioenriched Spirooxindole-Based 4-Methyleneazetidines
Source: Molecules. 2017 Nov 21;22(11):2016. doi: 10.3390/molecules22112016 (PMC6150293; doi:10.3390/molecules22112016)
Supplement: Supplementary file 1 [file molecules-22-02016-s001.pdf]

Supporting Information

for

Organocatalytic Access to Enantioenriched Spirooxindole-Based 4-Methyleneazetidines

Giulia Rainoldi,[1] Matteo Faltracco,[2] Claudia Spatti,[1] Alessandra Silvani[1,*] and Giordano Lesma[1]

[1]Dipartimento di Chimica, Università degli Studi di Milano, via Golgi 19, Milano, 20133, Italy;
[2]Department of Chemistry & Pharmaceutical Sciences and Amsterdam Institute of Molecules Medicines & Systems (AIMMS), Vrije Universiteit Amsterdam, De Boelelaan 1108, 1081 HZ Amsterdam, The Netherlands;

* Correspondence: alessandra.silvani@unimi.it; Tel.: +39-0250314080

Table of contents

Copies of ^{1}H and ^{13}C NMR spectra (all new compounds) **S2-S22**

HPLC chromatograms (compounds **6a-h, 6k**) **S23-S32**

(*E*)-N-(1-propyl-2-oxoindolin-3-ylidene)-2-methylpropane-2-sulfinamide:

^{1}H NMR (300 MHz, $CDCl_3$)

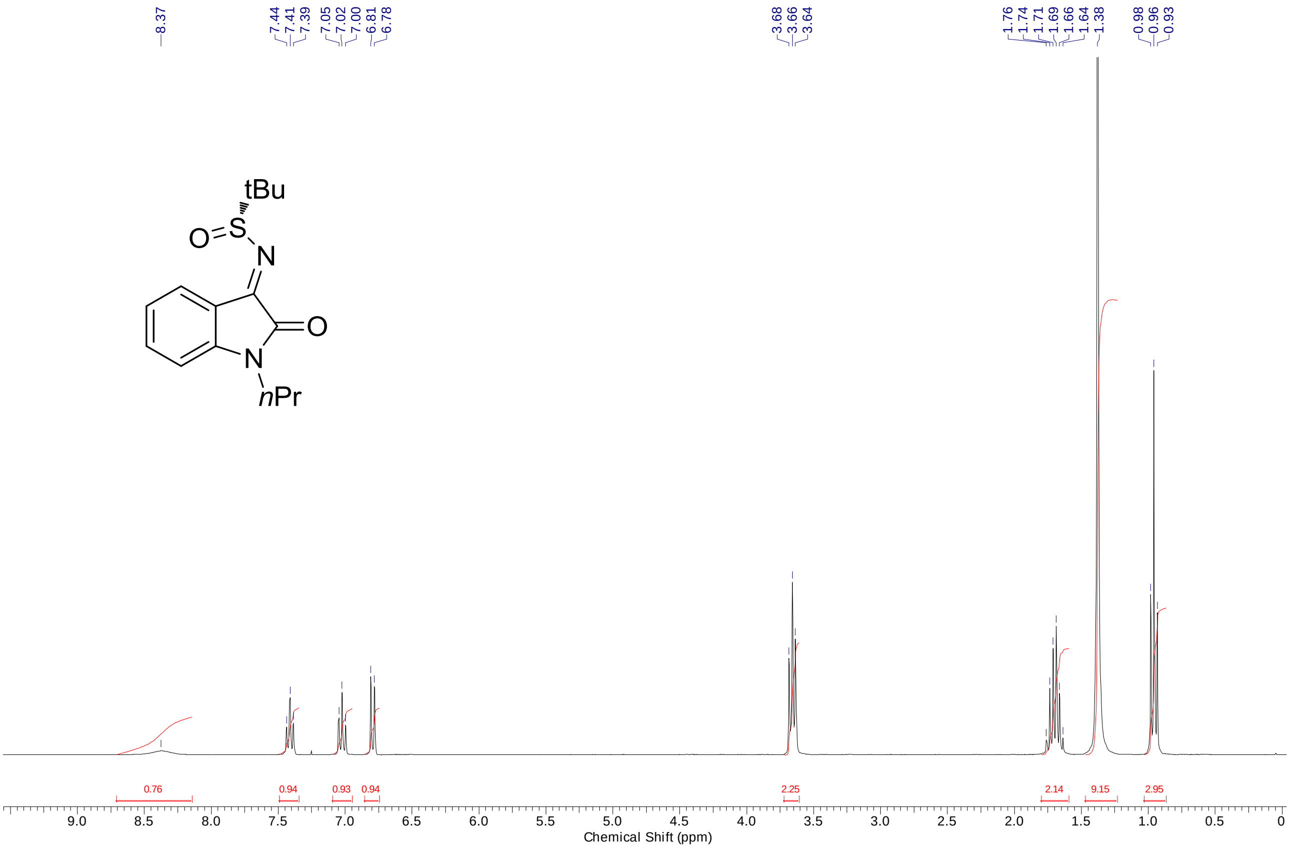

(*E*)-N-(1-propyl-2-oxoindolin-3-ylidene)-2-methylpropane-2-sulfinamide:

^{13}C NMR (101 MHz, APT, $CDCl_3$)

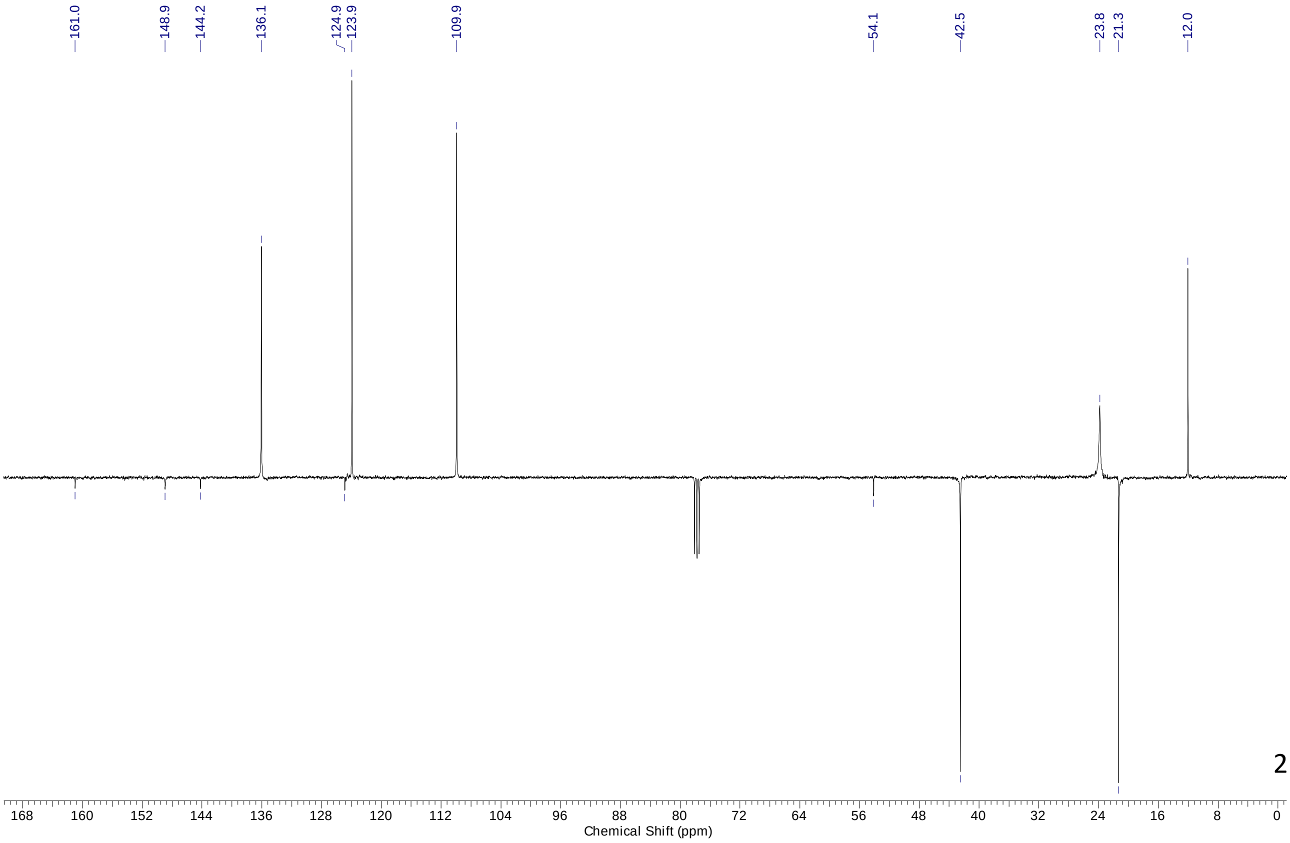

(*E*)-N-(1-isopropyl-2-oxoindolin-3-ylidene)-2-methylpropane-2-sulfinamide:

^{1}H NMR (300 MHz, $CDCl_3$)

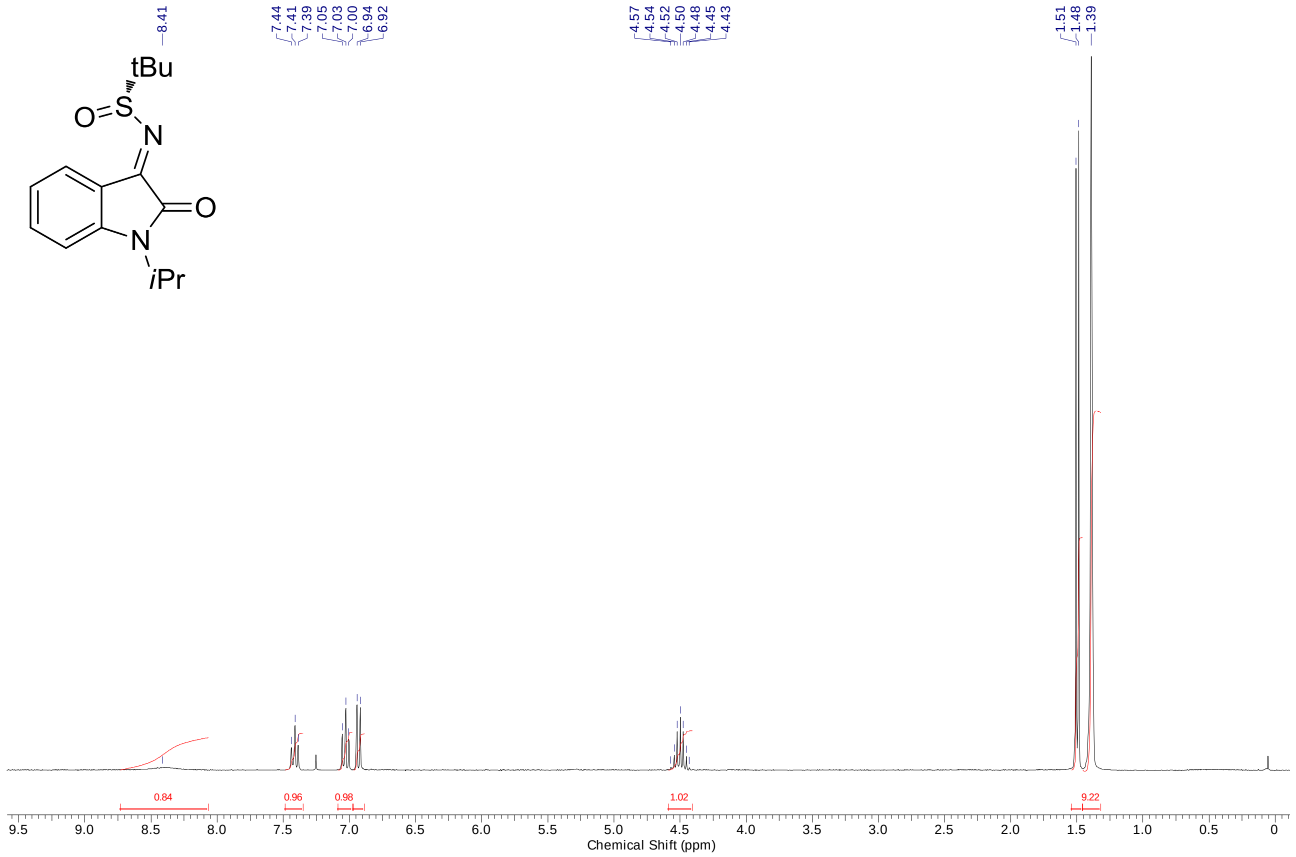

(*E*)-N-(1-isopropyl-2-oxoindolin-3-ylidene)-2-methylpropane-2-sulfinamide:

^{13}C NMR (101 MHz, APT, $CDCl_3$)

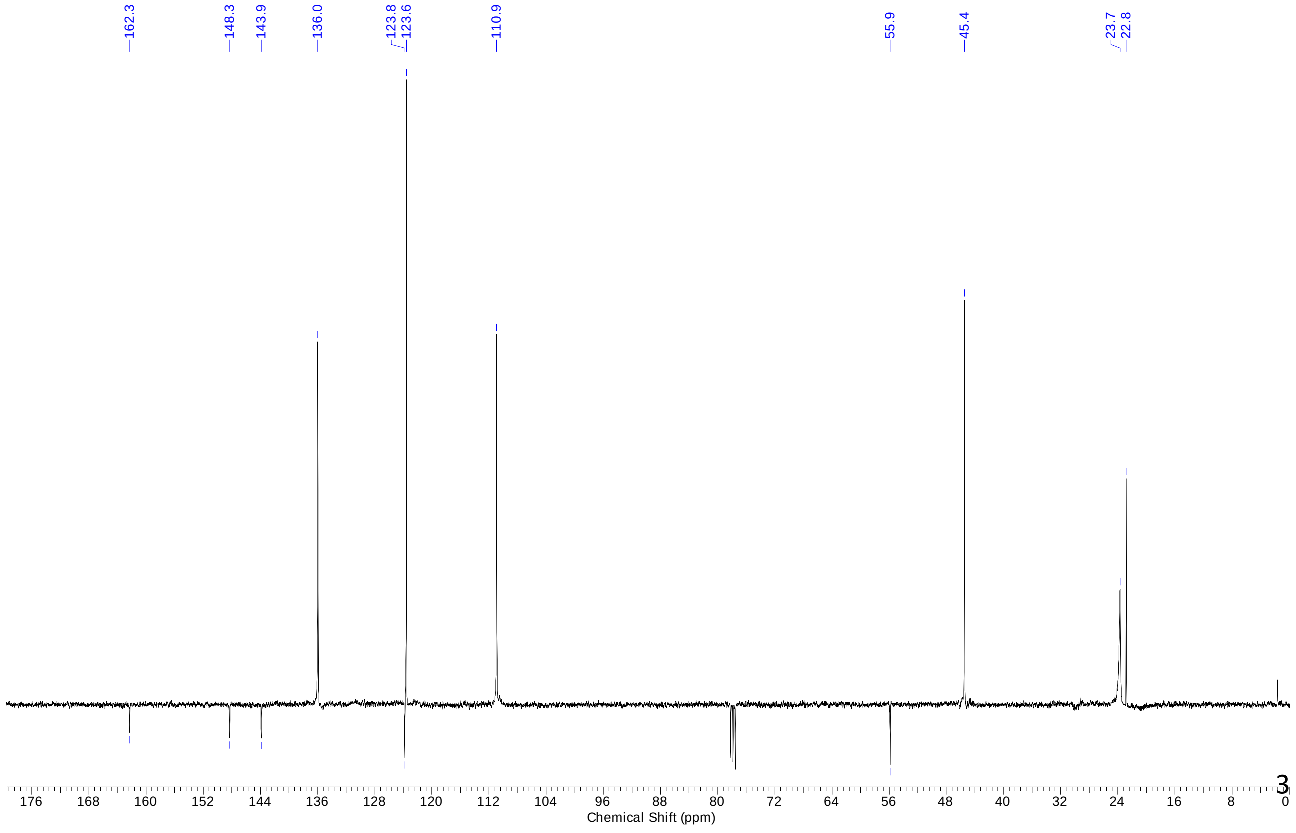

(*E*)-2-methyl-N-(2-oxo-1-phenylindolin-3-ylidene)propane-2-sulfinamide:

^{1}H NMR (300 MHz, $CDCl_3$)

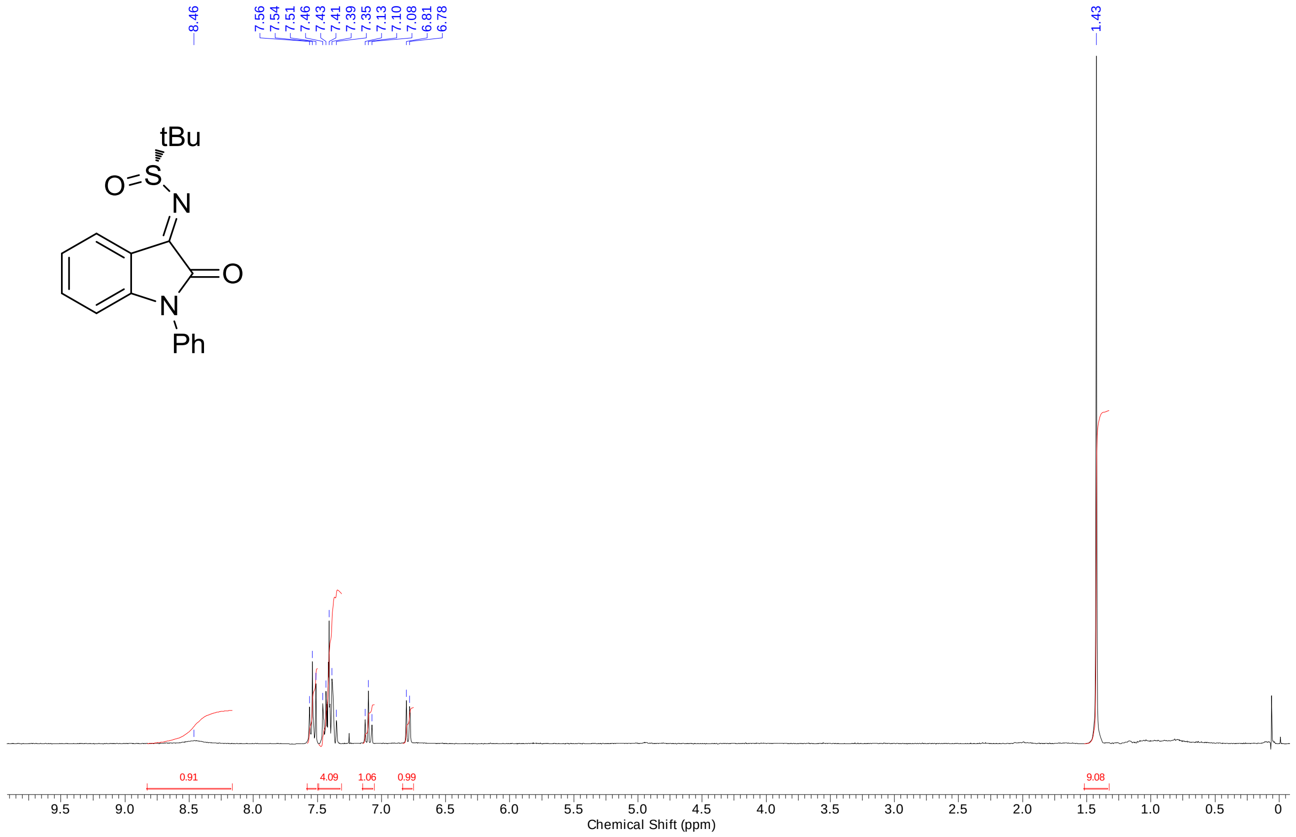

(*E*)-2-methyl-N-(2-oxo-1-phenylindolin-3-ylidene)propane-2-sulfinamide:

^{13}C NMR (101 MHz, APT, $CDCl_3$)

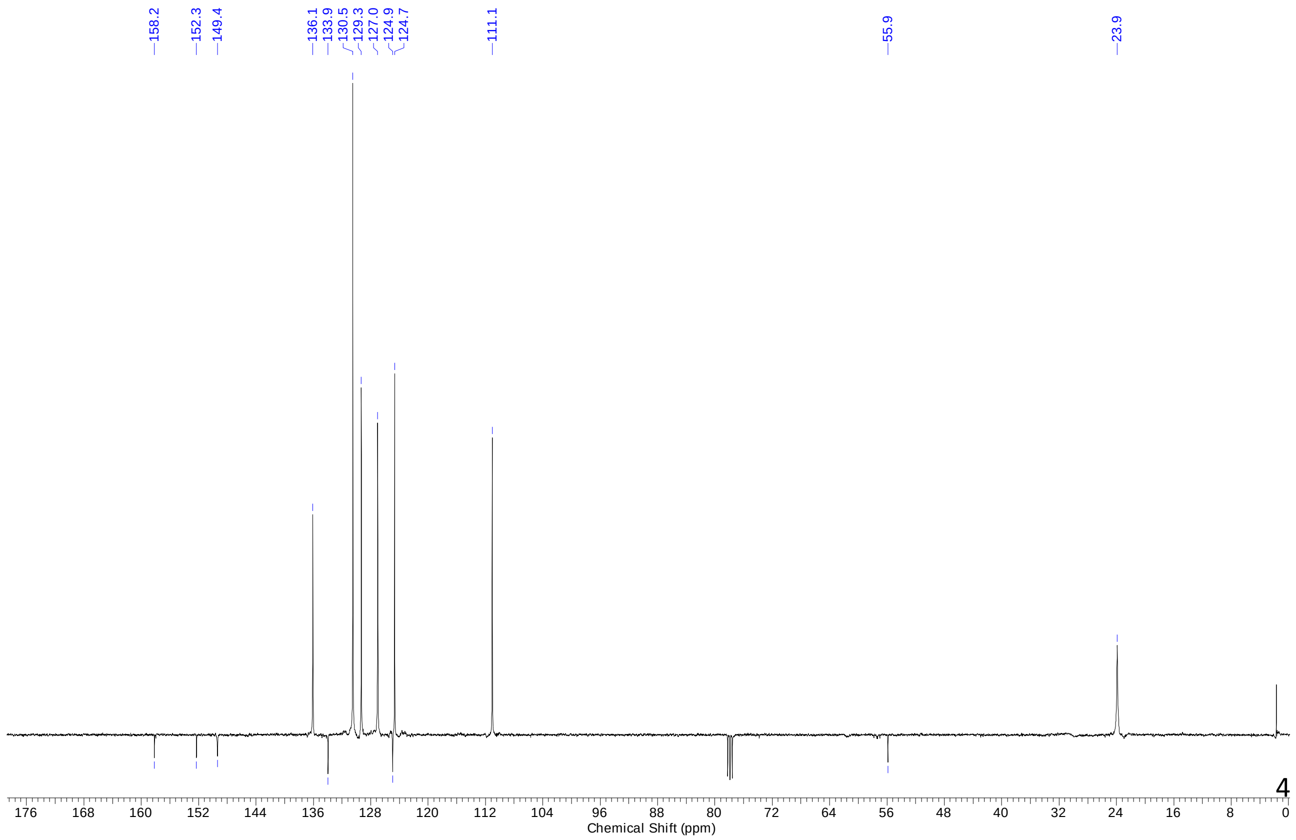

Compound 1c:

^{1}H NMR (300 MHz, $CDCl_3$)

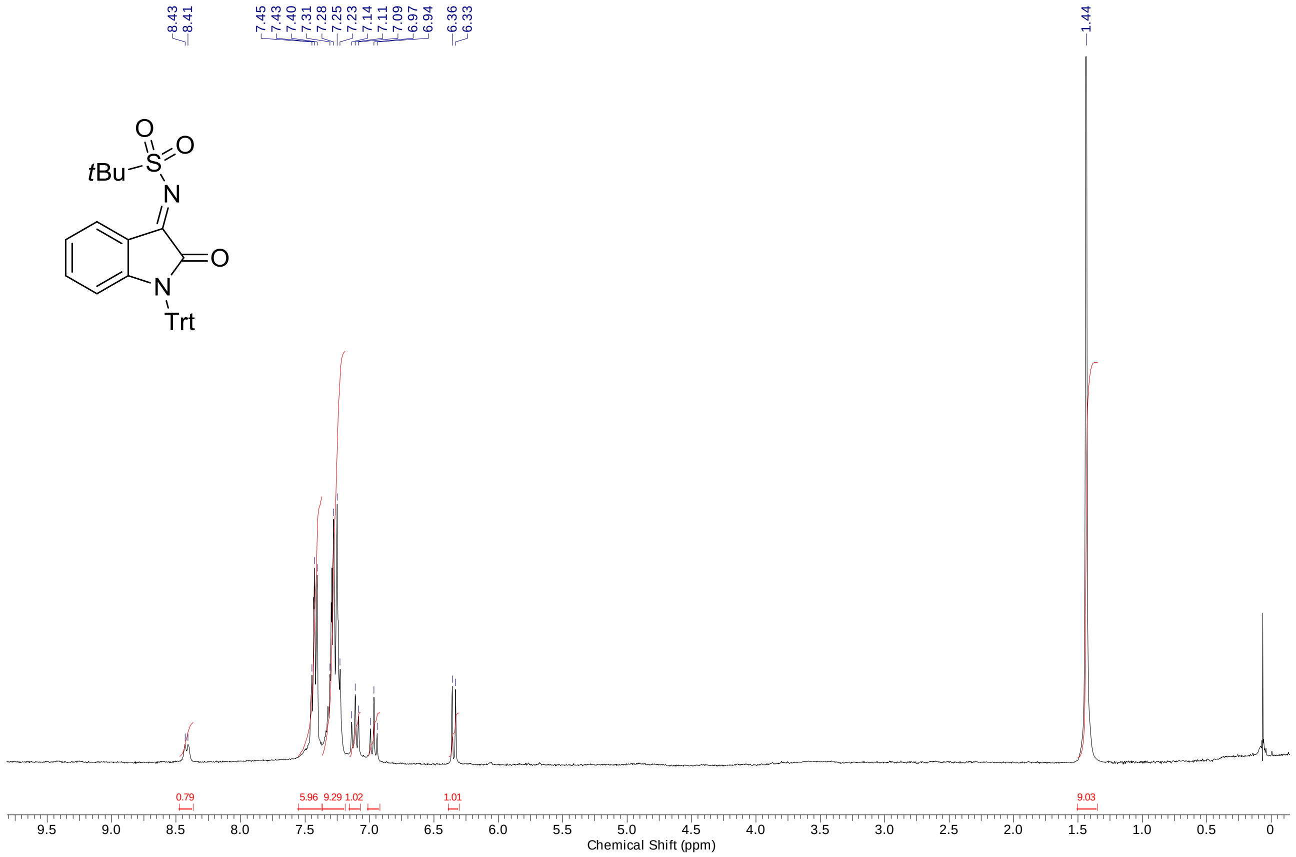

Compound 1c:

^{13}C NMR (101 MHz, APT, $CDCl_3$)

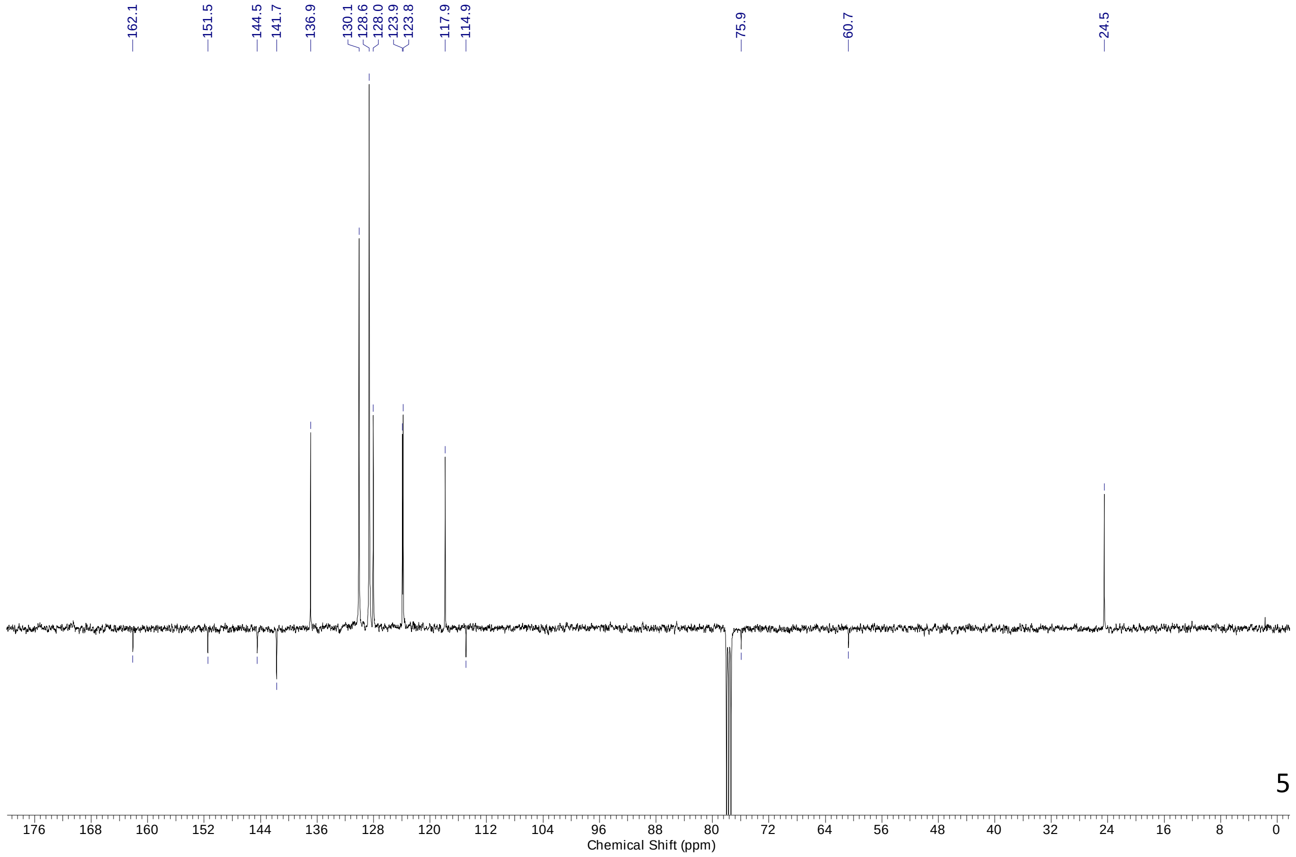

Compound 1d:

^{1}H NMR (300 MHz, $CDCl_3$)

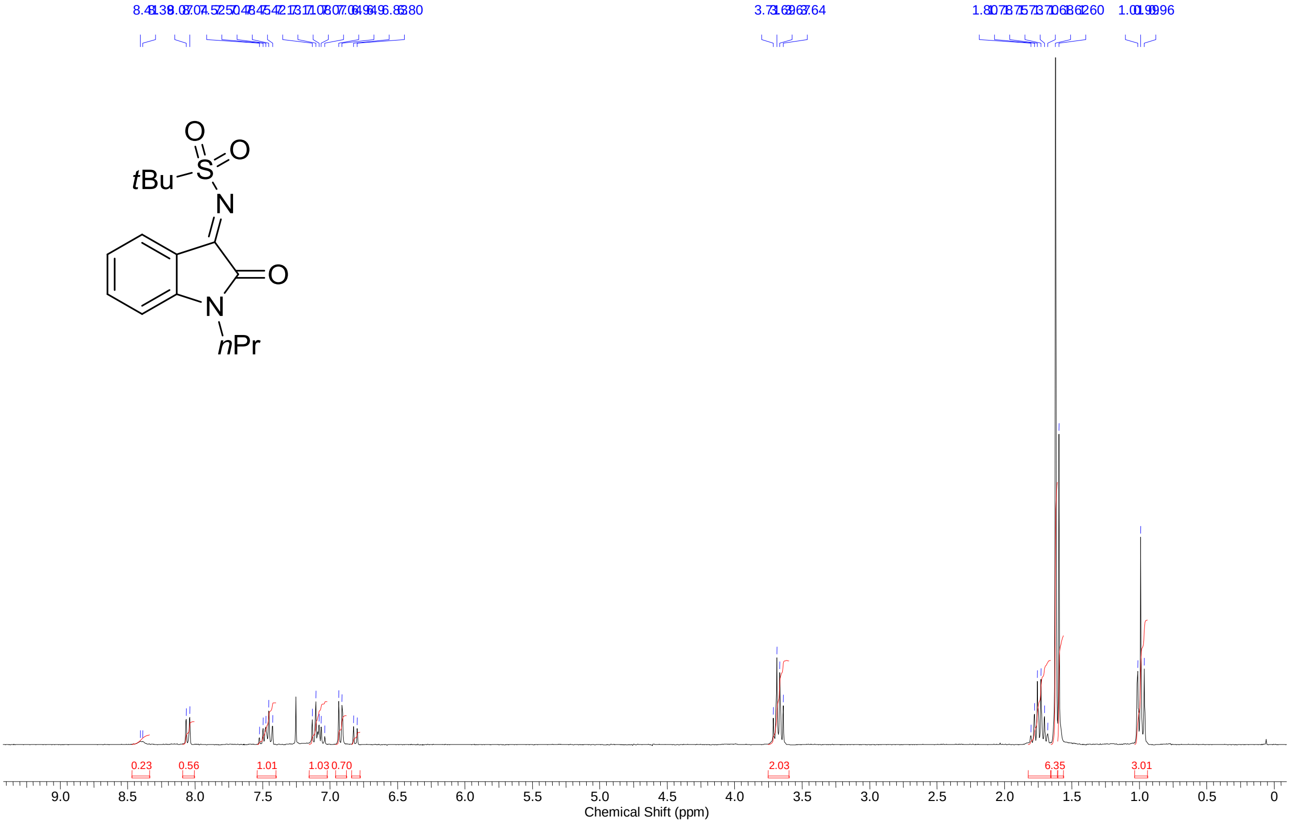

Compound 1d:

^{13}C NMR (75 MHz, APT, $CDCl_3$)

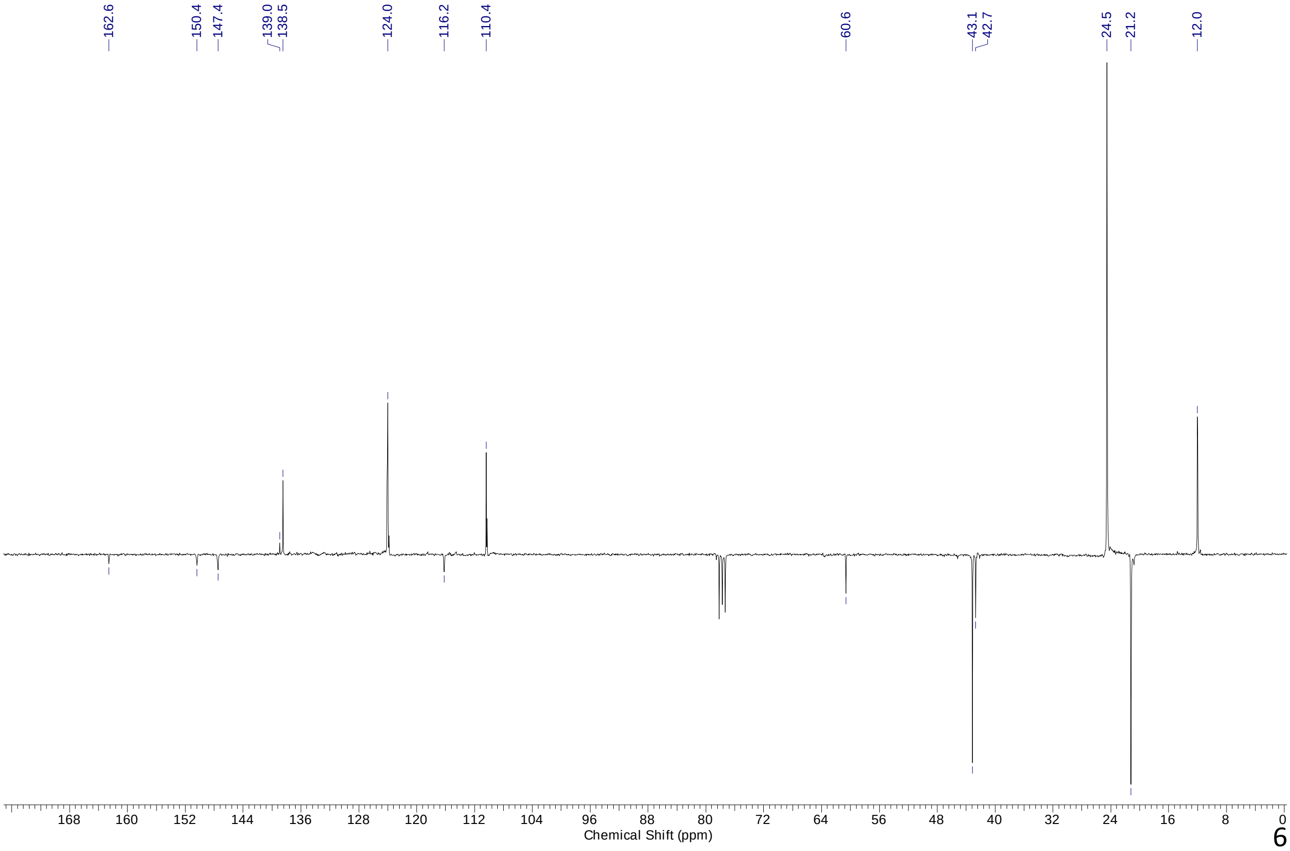

Compound 1e:

^{1}H NMR (400 MHz, $CDCl_3$)

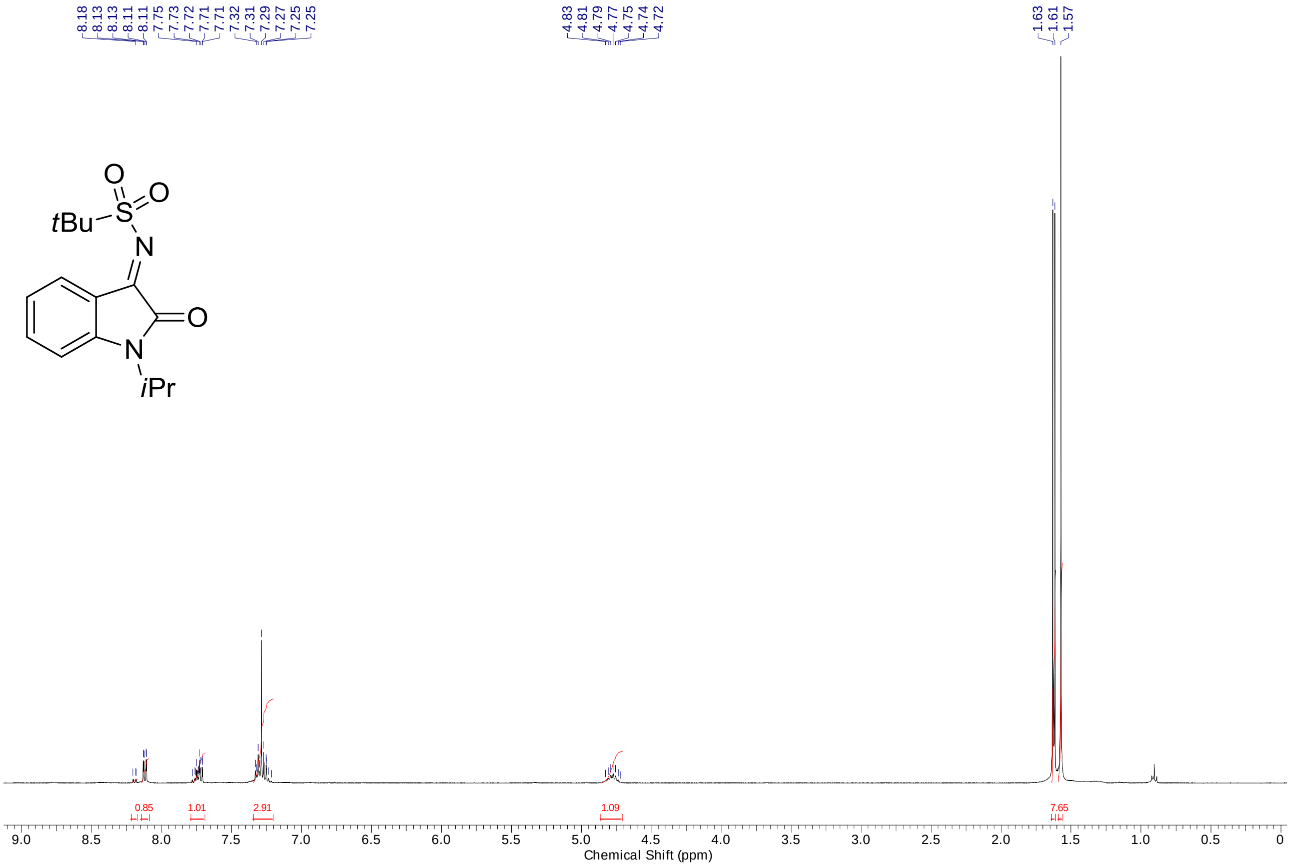

Compound 1e:

^{13}C NMR (101 MHz, APT, $CDCl_3$)

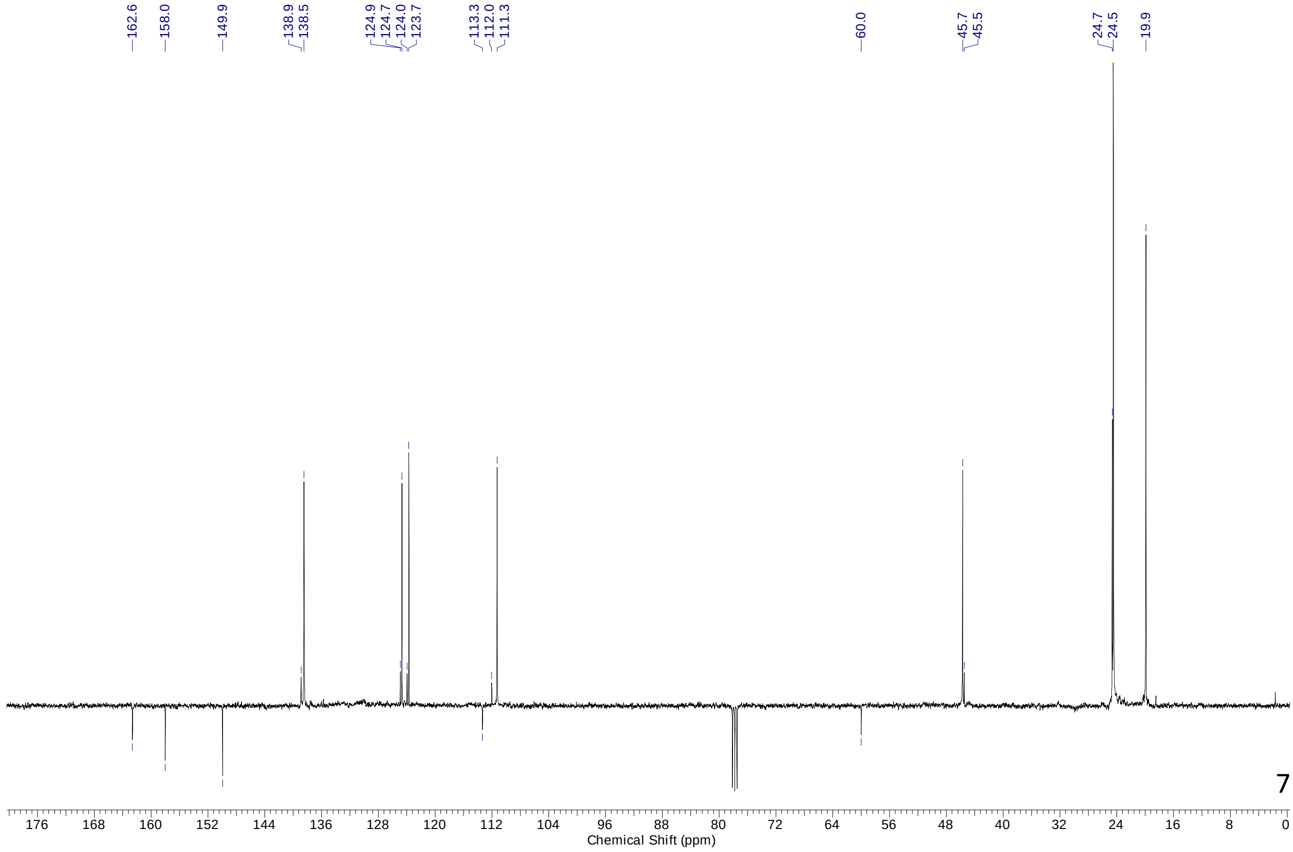

Compound 1f:

^{1}H NMR (400 MHz, $CDCl_3$)

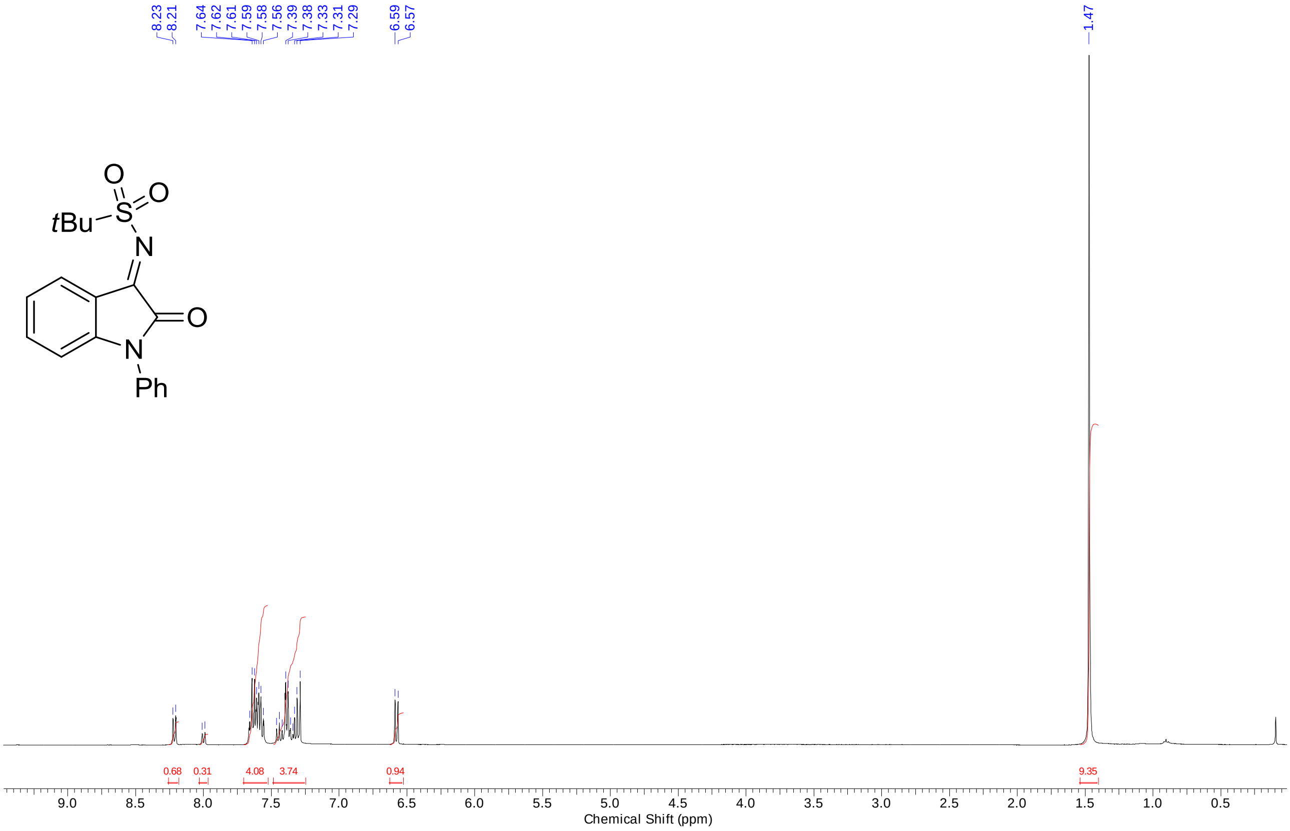

Compound 1f:

^{13}C NMR (101 MHz, APT, $CDCl_3$)

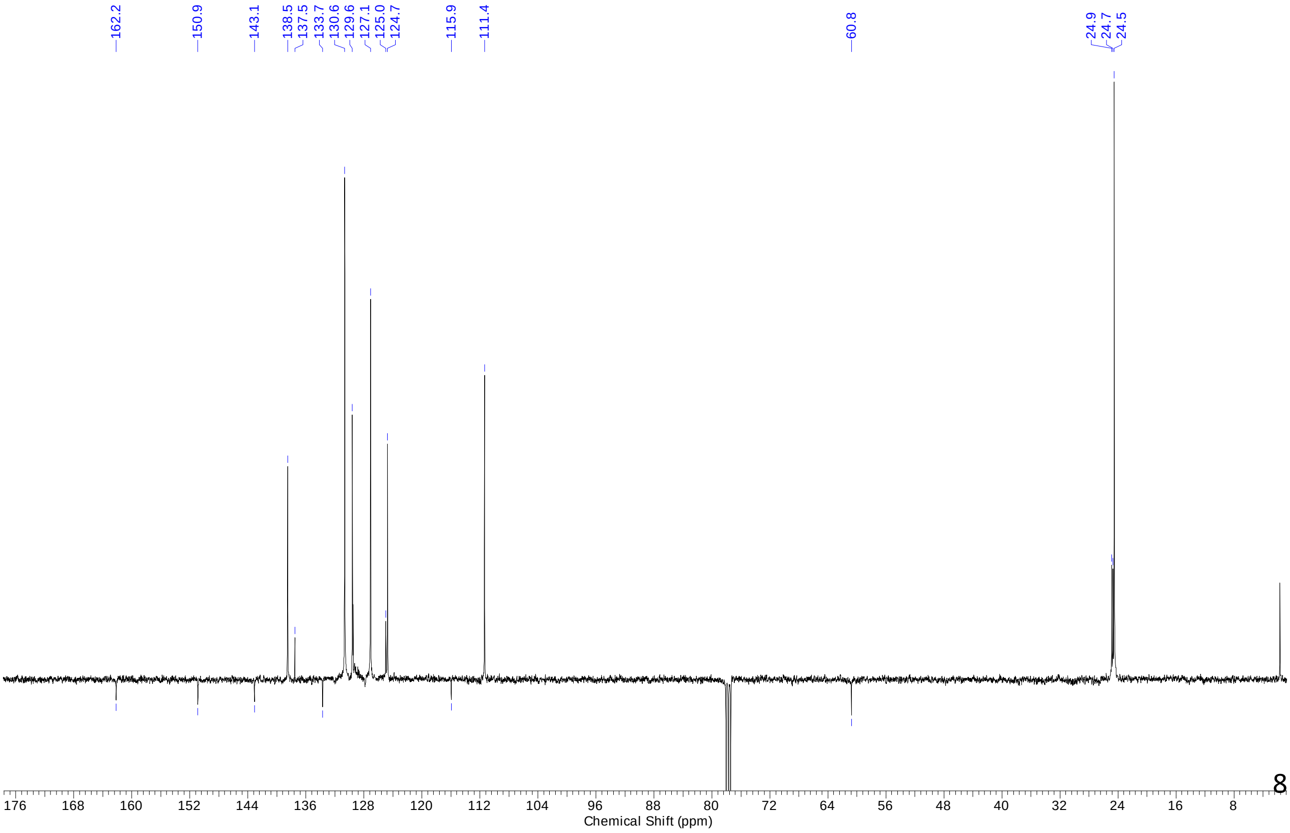

Compound 1g:

^{1}H NMR (400 MHz, $CDCl_3$)

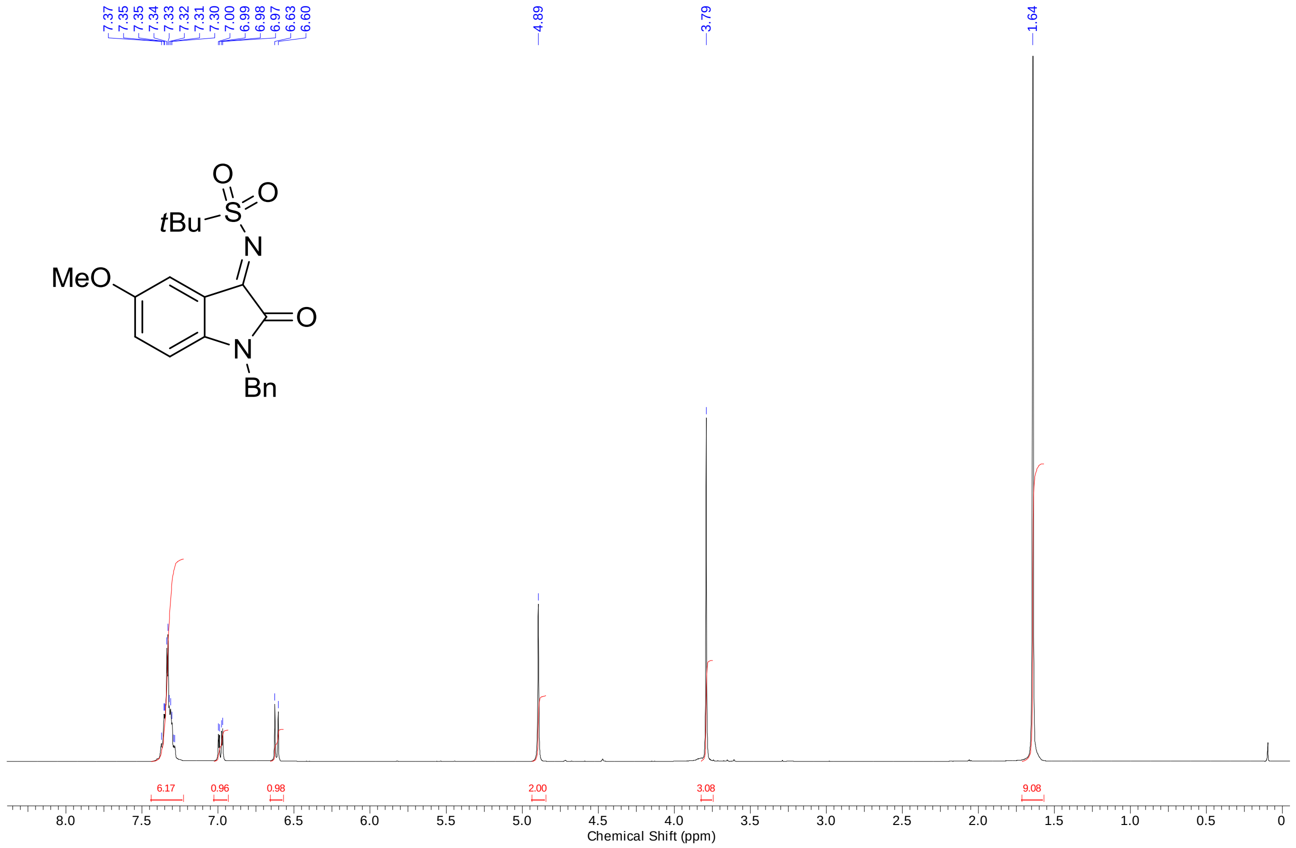

Compound 1g:

^{13}C NMR (101 MHz, APT, $CDCl_3$)

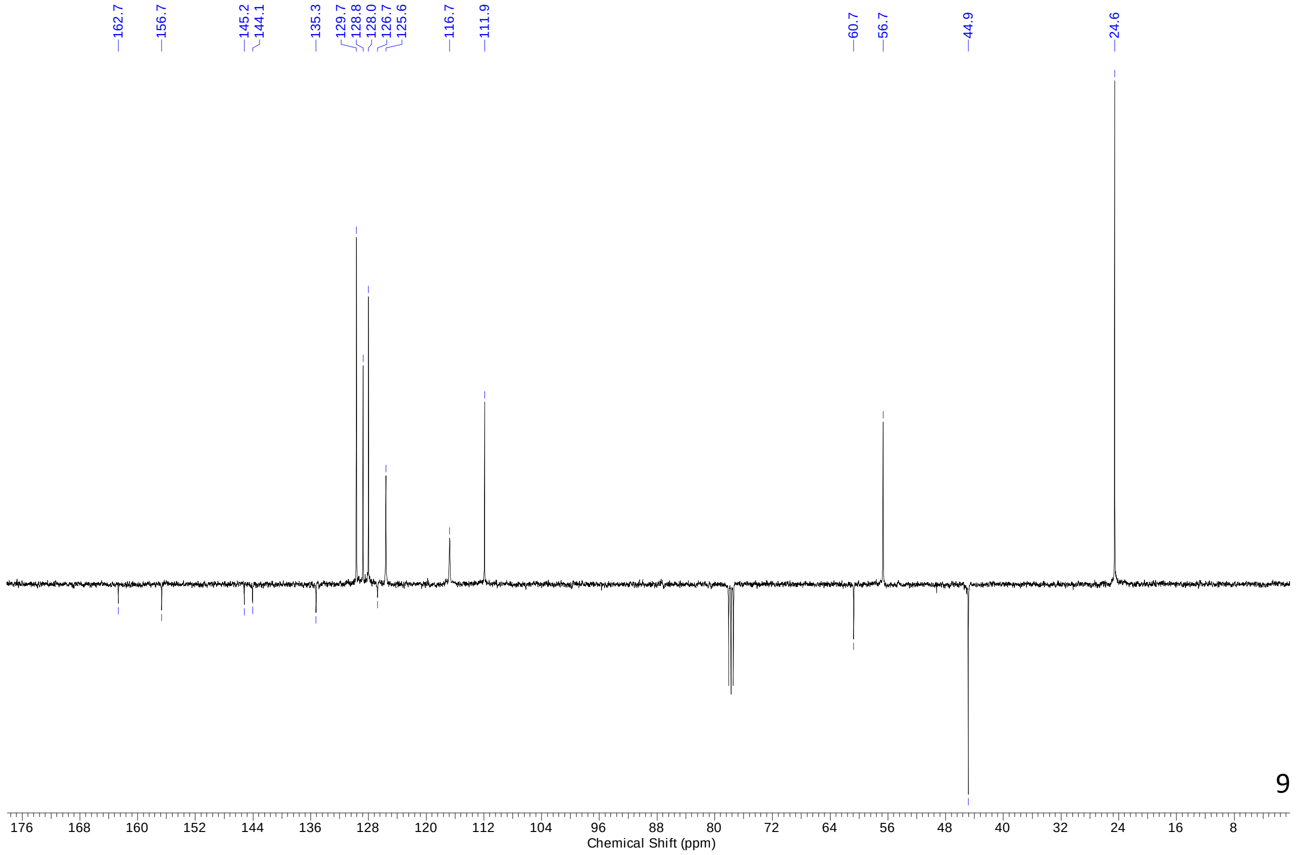

Compound 1h:

^{1}H NMR (400 MHz, $CDCl_3$)

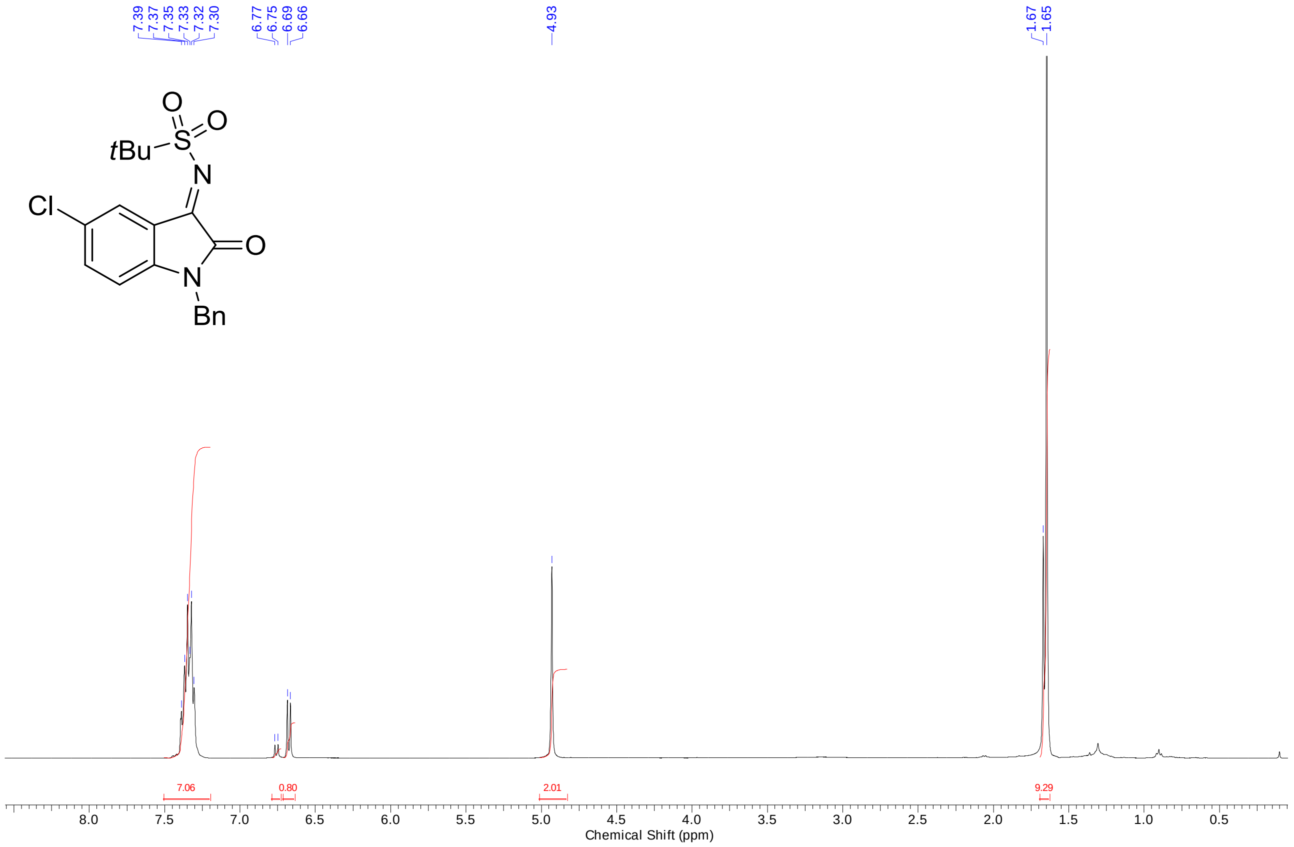

Compound 1h:

^{13}C NMR (101 MHz, APT, $CDCl_3$)

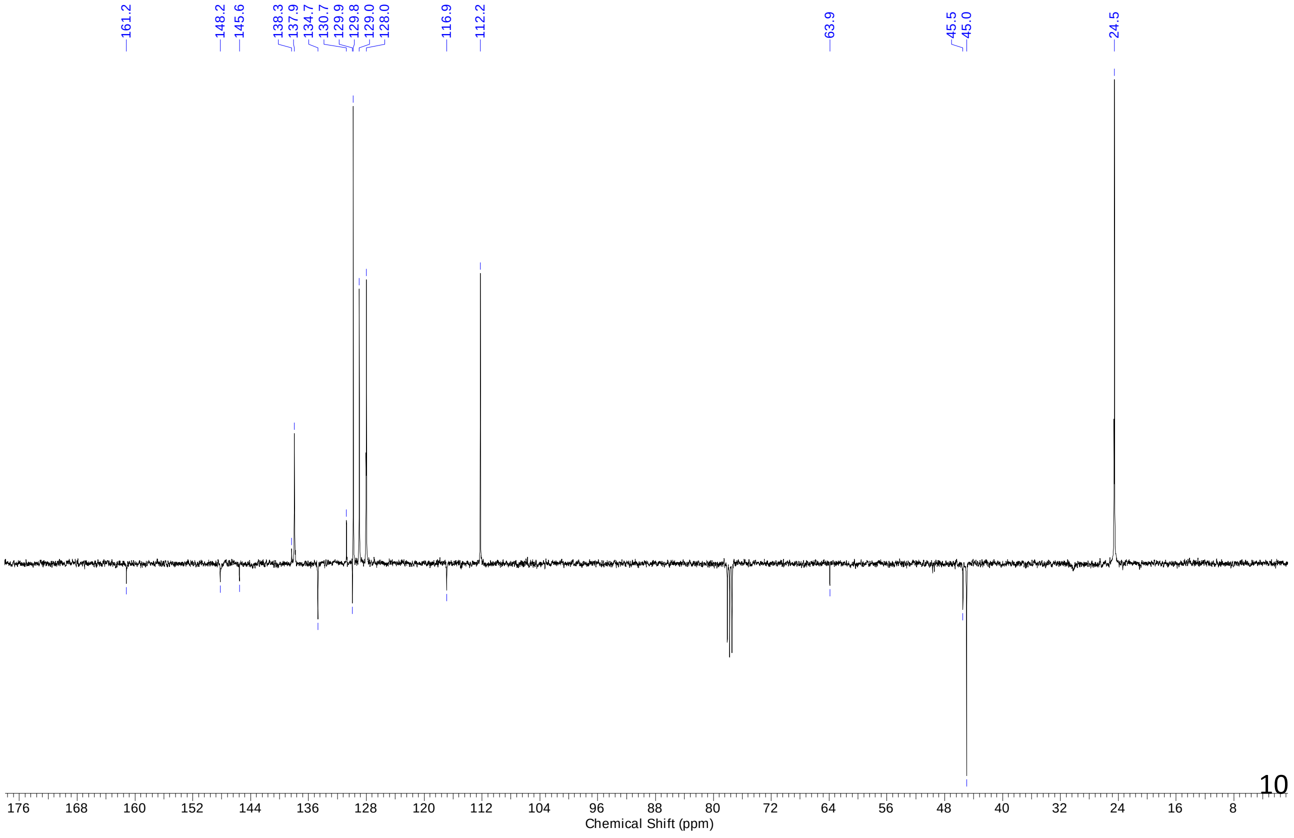

Compound 6a: ^{1}H NMR (300 MHz, $CDCl_3$)

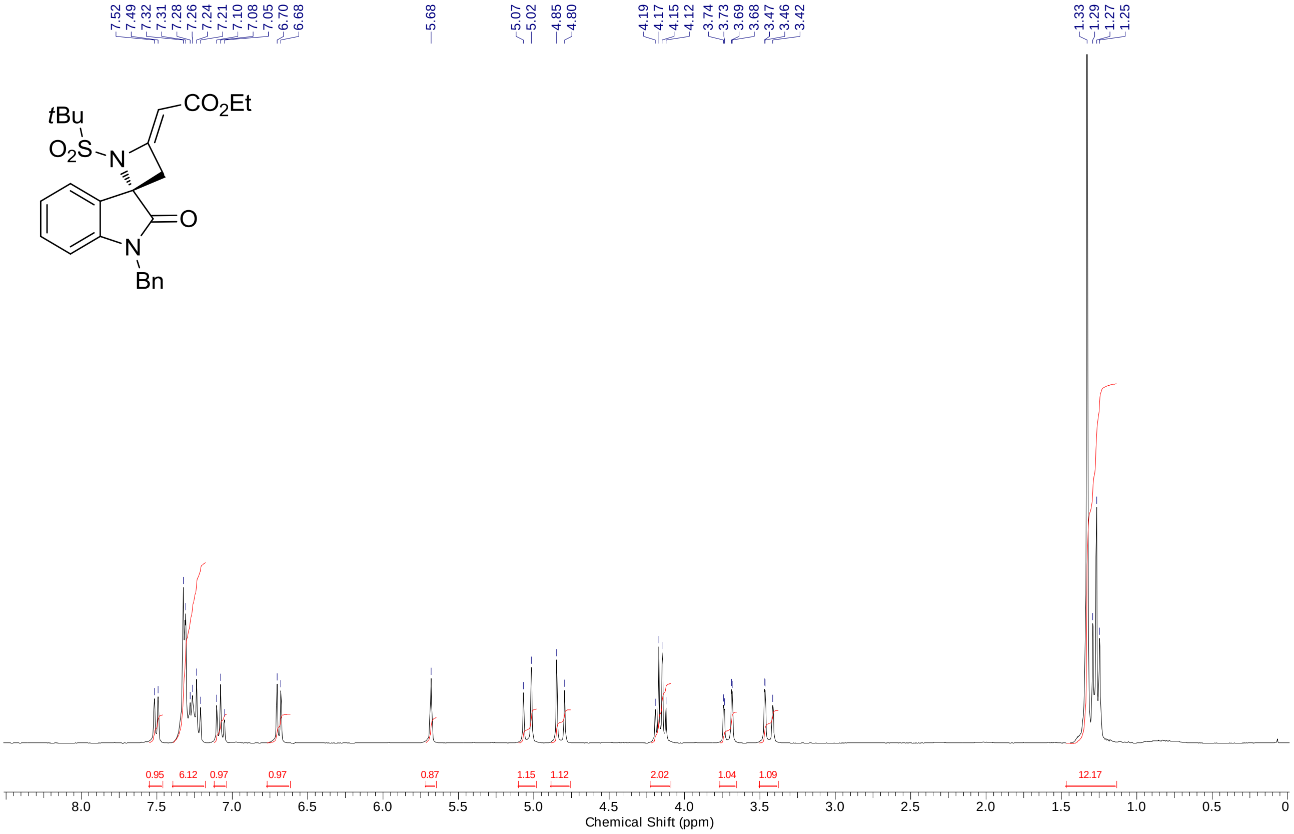

Compound 6a: ^{13}C NMR (101 MHz, APT, $CDCl_3$)

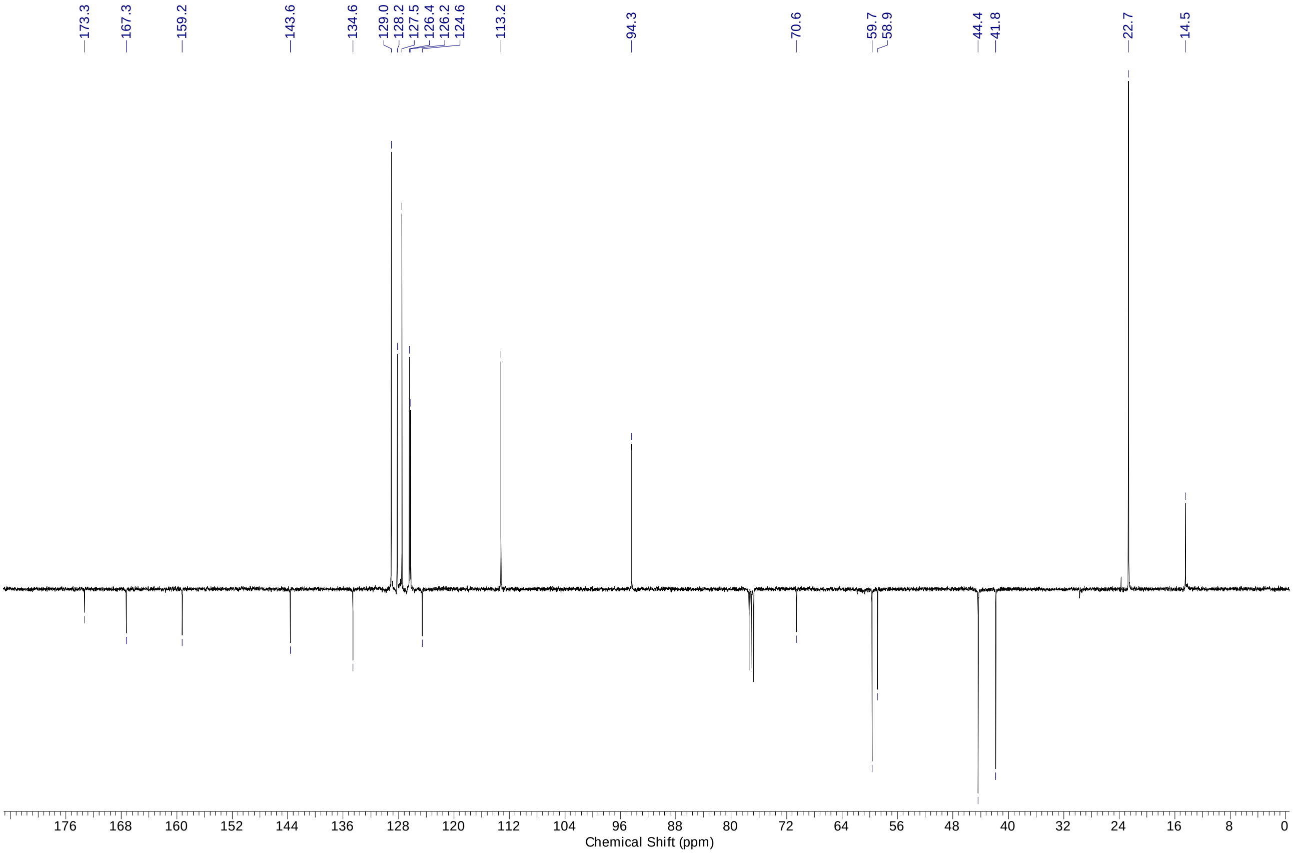

Compound 6b: ^{1}H NMR (400 MHz, $CDCl_3$)

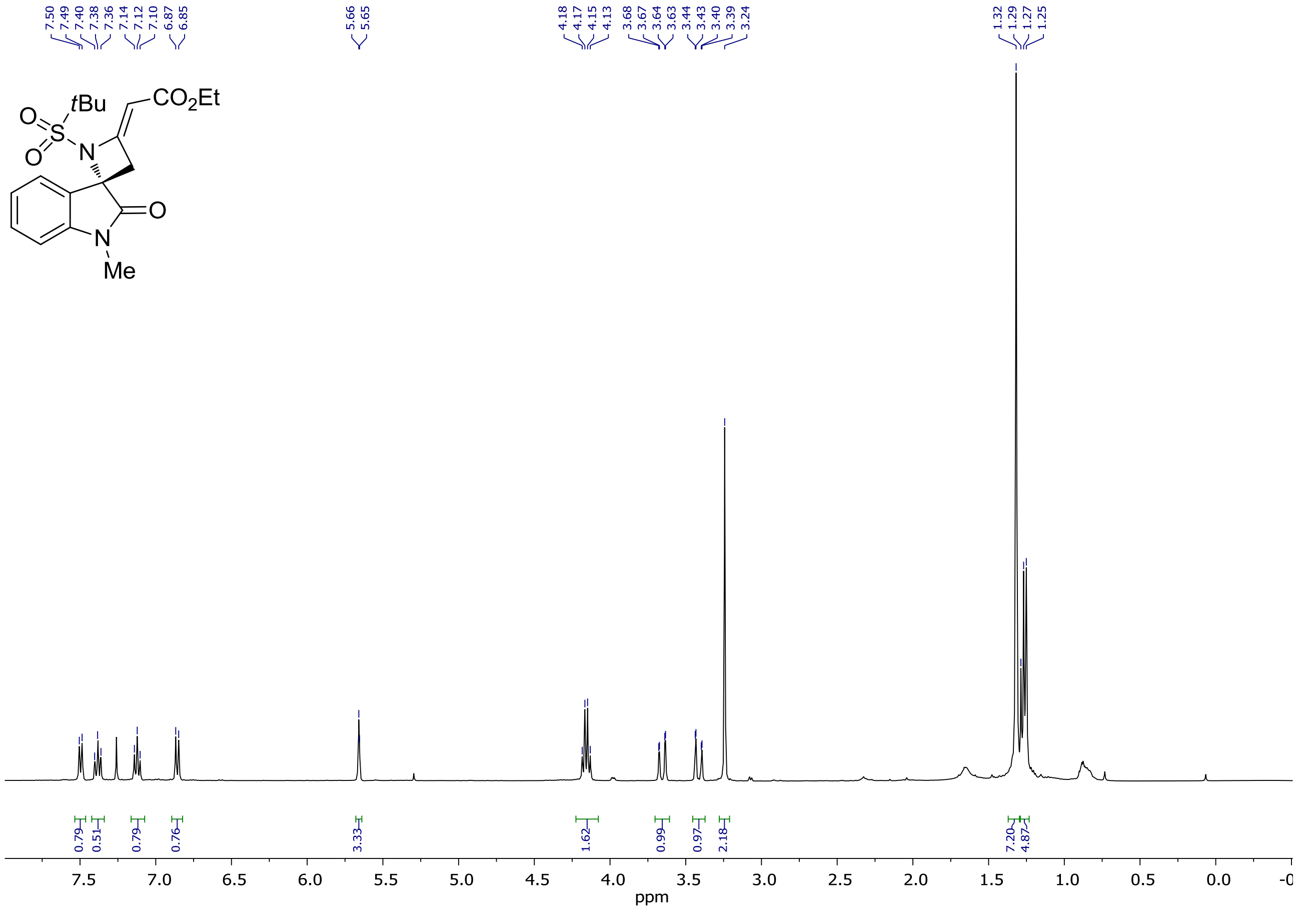

Compound 6b: ^{13}C NMR (101 MHz, $CDCl_3$)

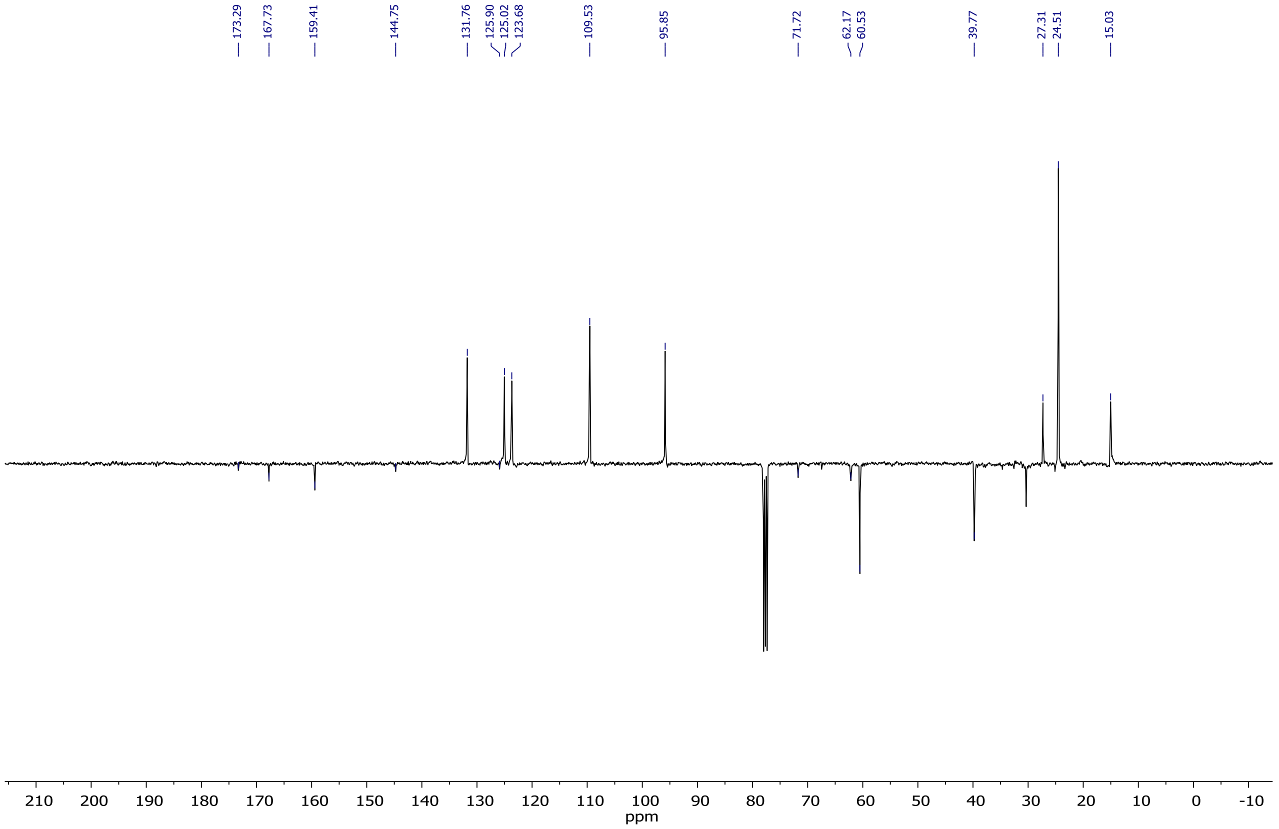

Compound 6c: ^{1}H NMR (300 MHz, $CDCl_3$)

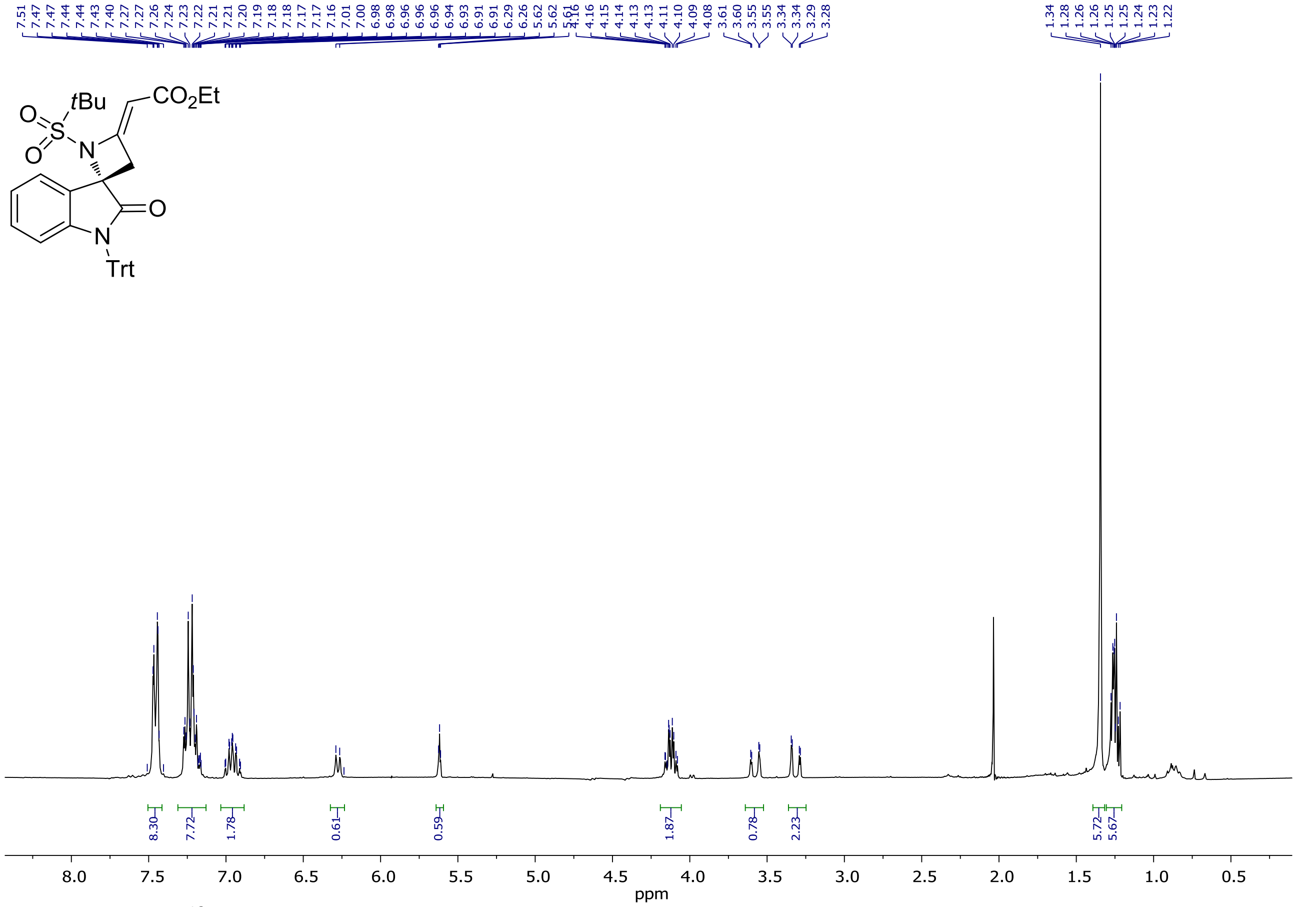

Compound 6c: ^{13}C NMR (75 MHz, $CDCl_3$)

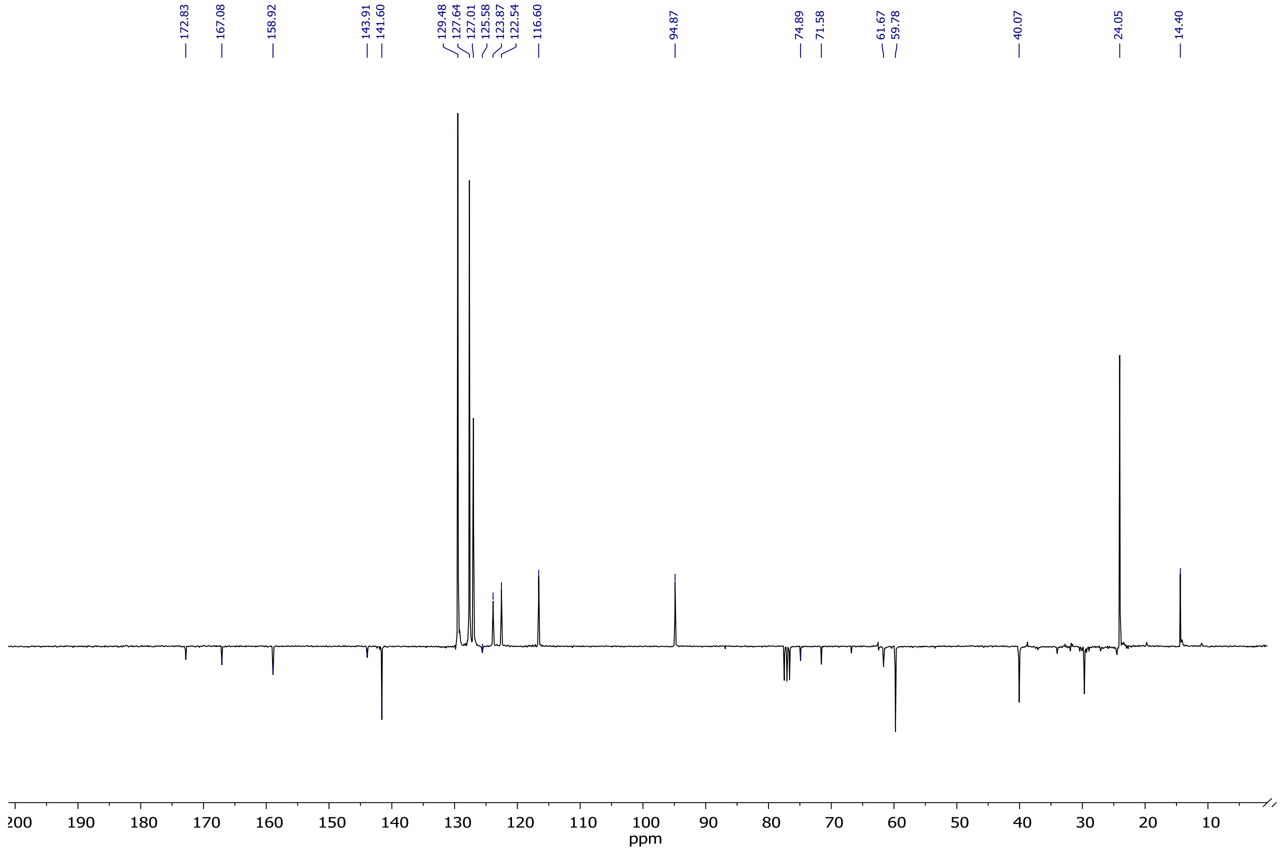

Compound 6d: ^{1}H NMR (300 MHz, $CDCl_3$)

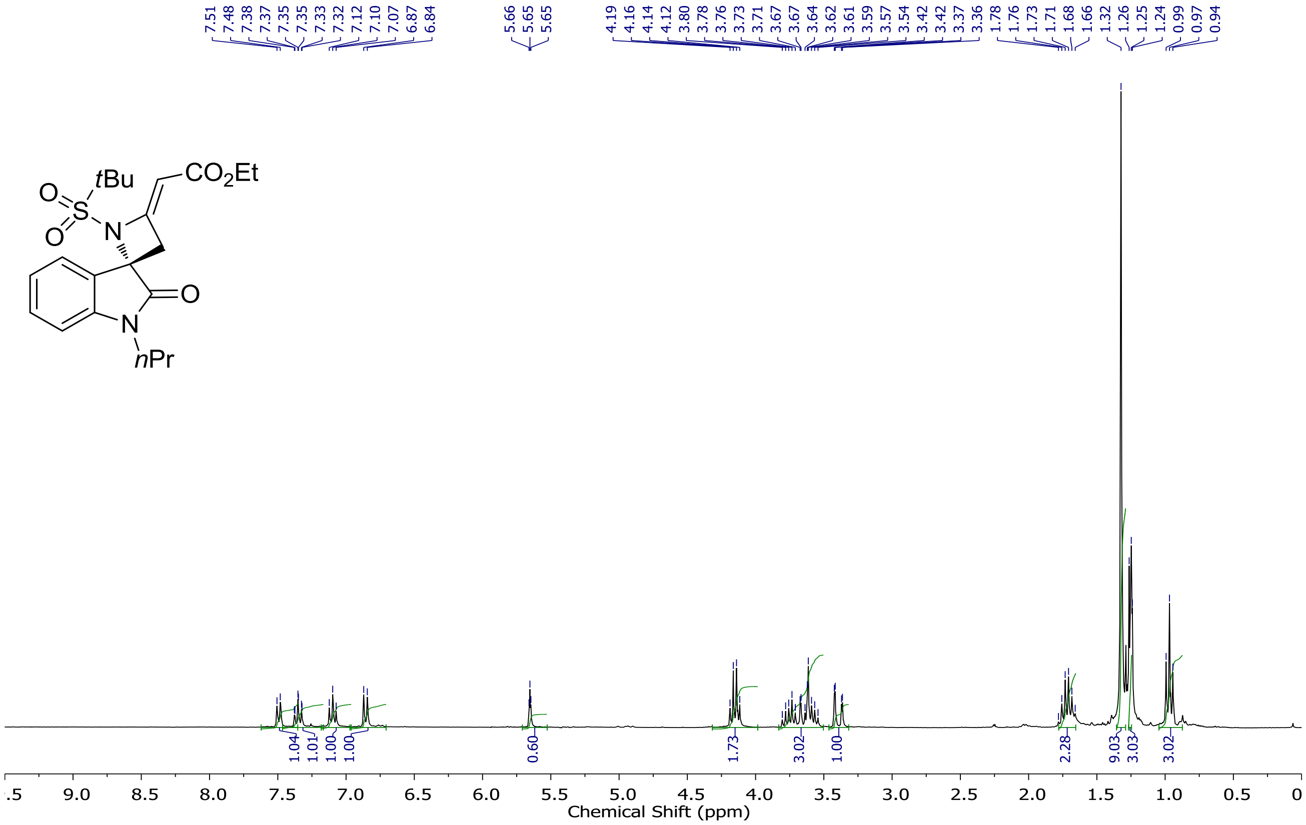

Compound 6d: ^{13}C NMR (101 MHz, APT, $CDCl_3$)

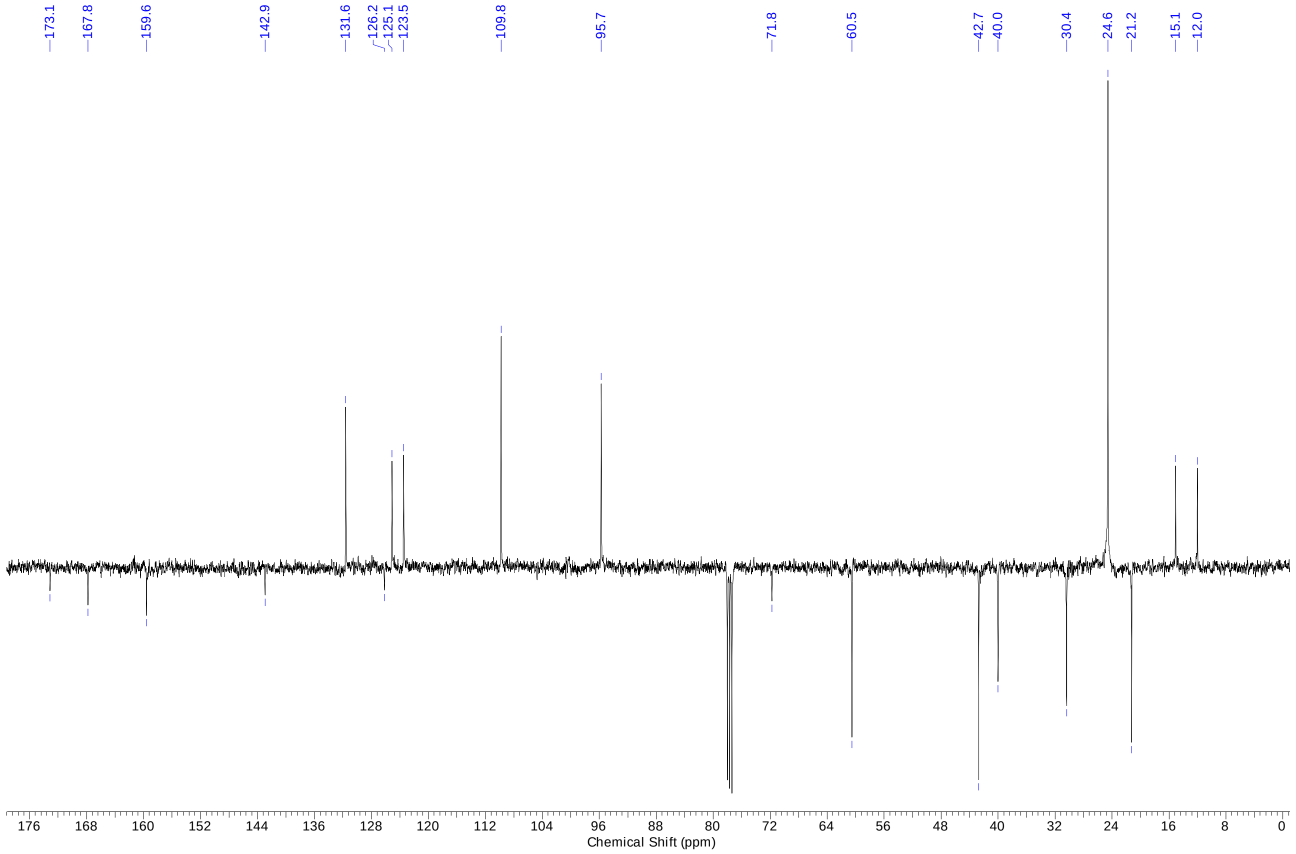

Compound 6e: ^{1}H NMR (300 MHz, $CDCl_3$)

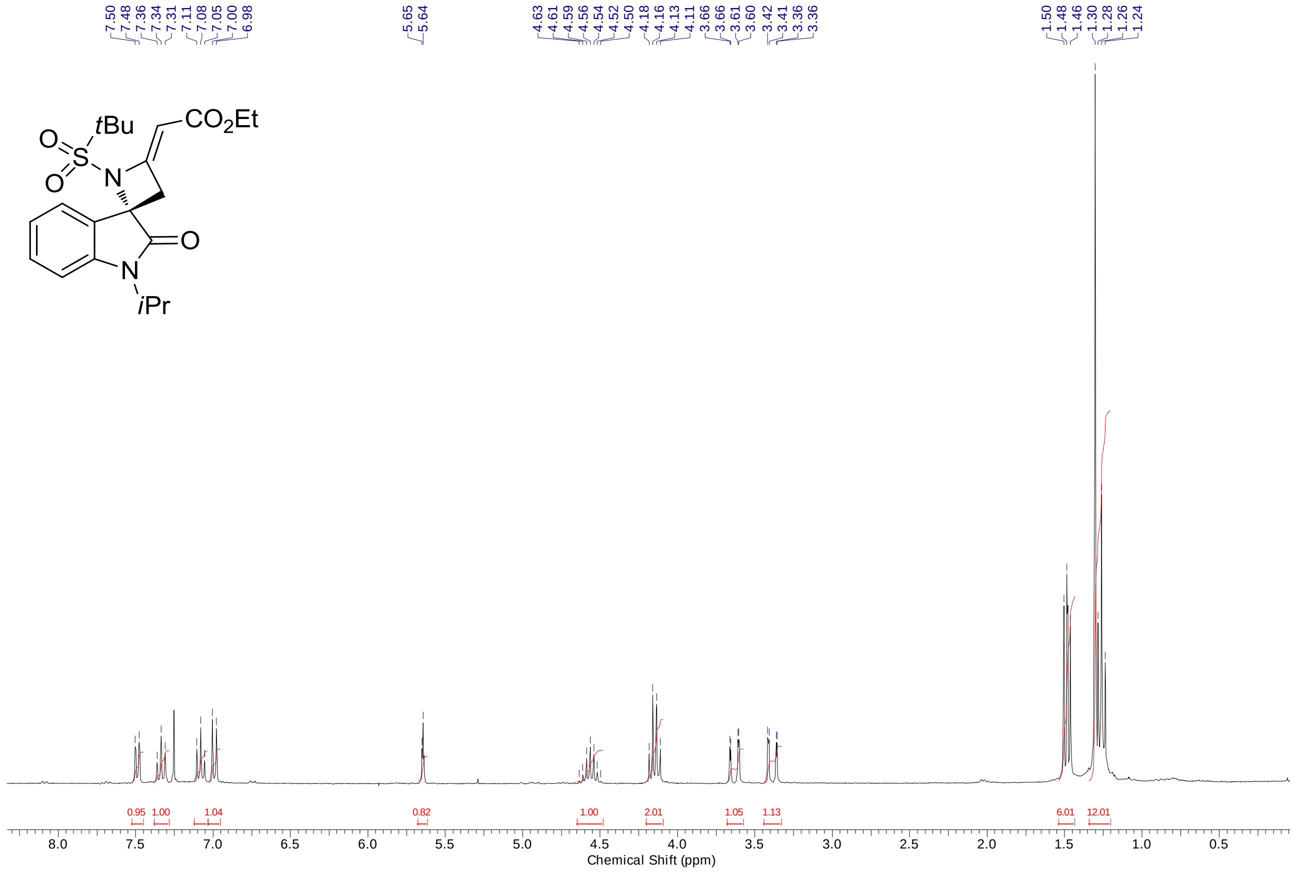

Compound 6e: ^{13}C NMR (101 MHz, APT, $CDCl_3$)

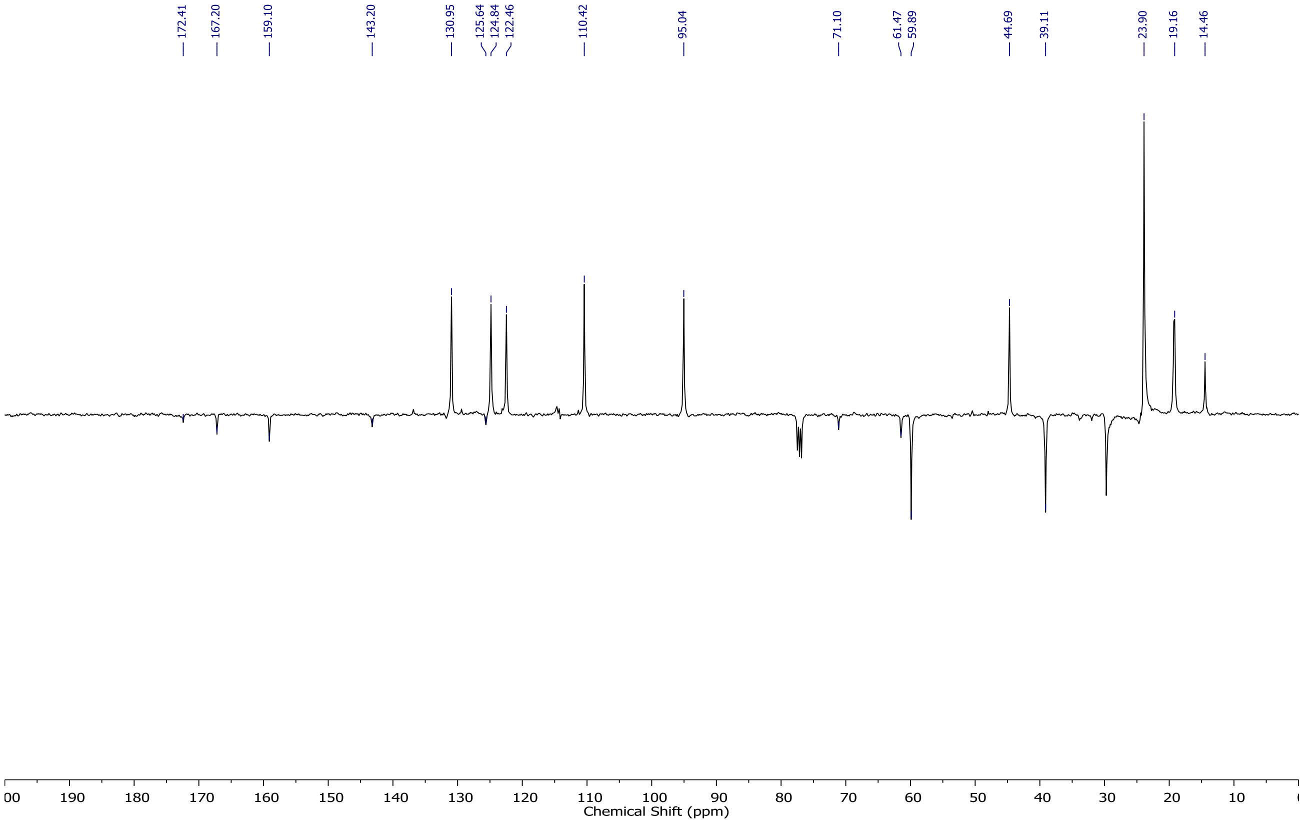

Compound 6f: ^{1}H NMR (300 MHz, $CDCl_3$)

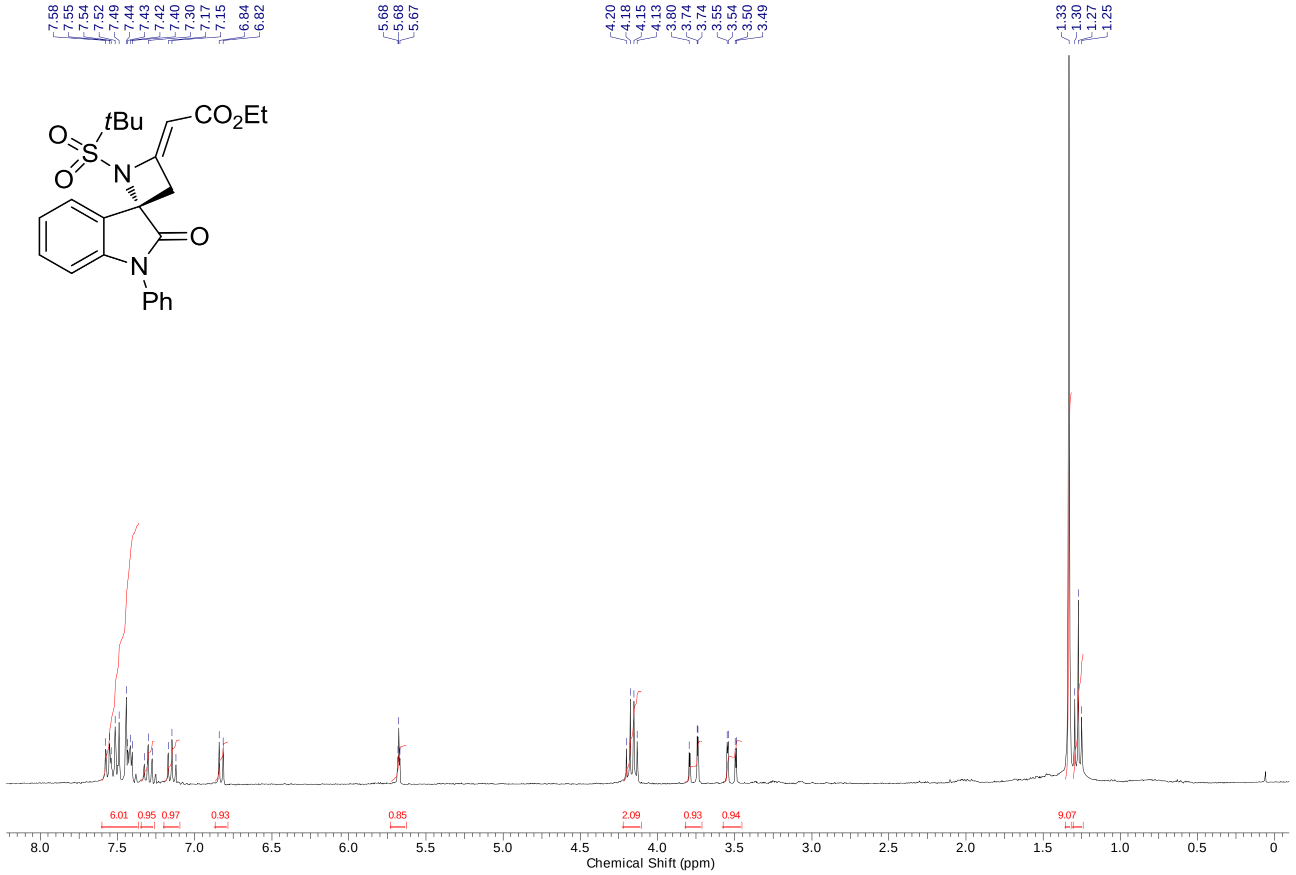

Compound 6f: ^{13}C NMR (101 MHz, APT, $CDCl_3$)

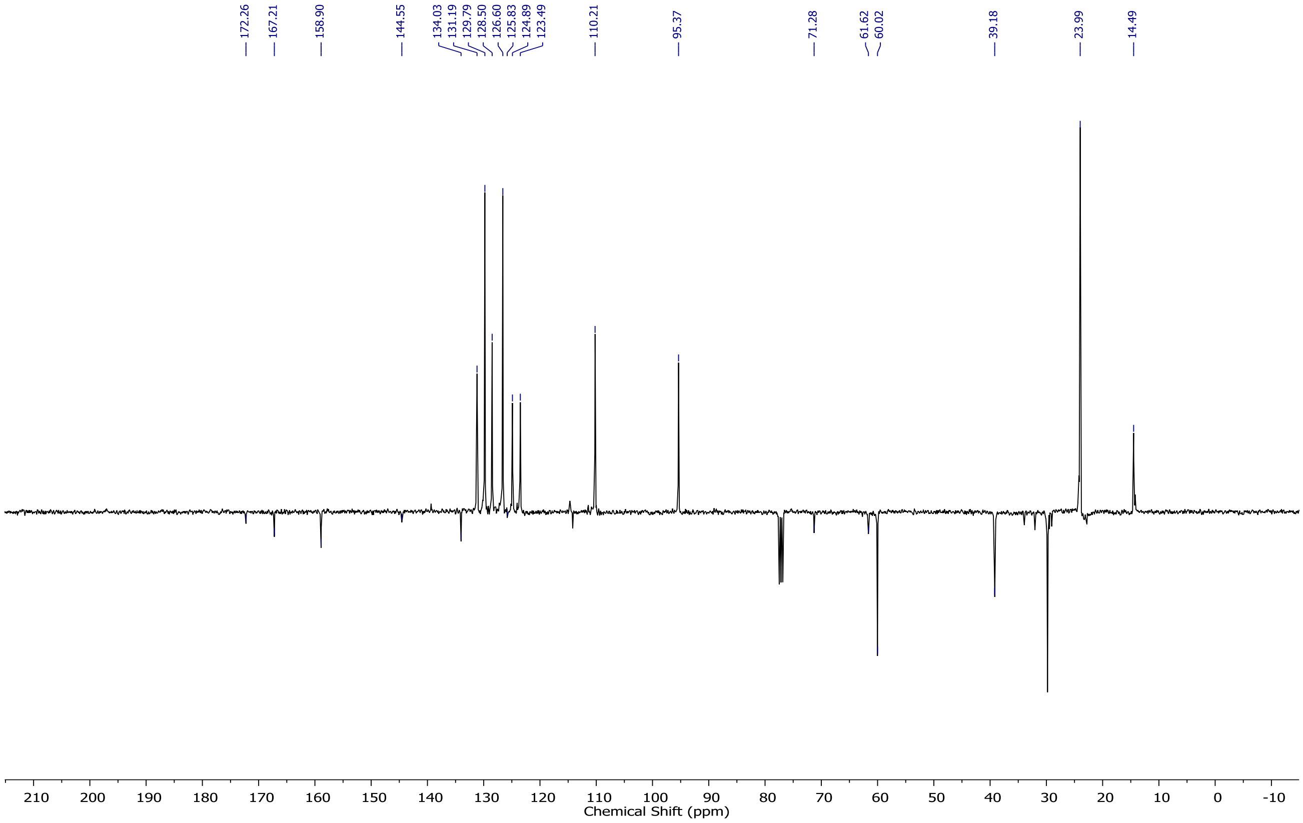

Compound 6g: ^{1}H NMR (400 MHz, $CDCl_3$)

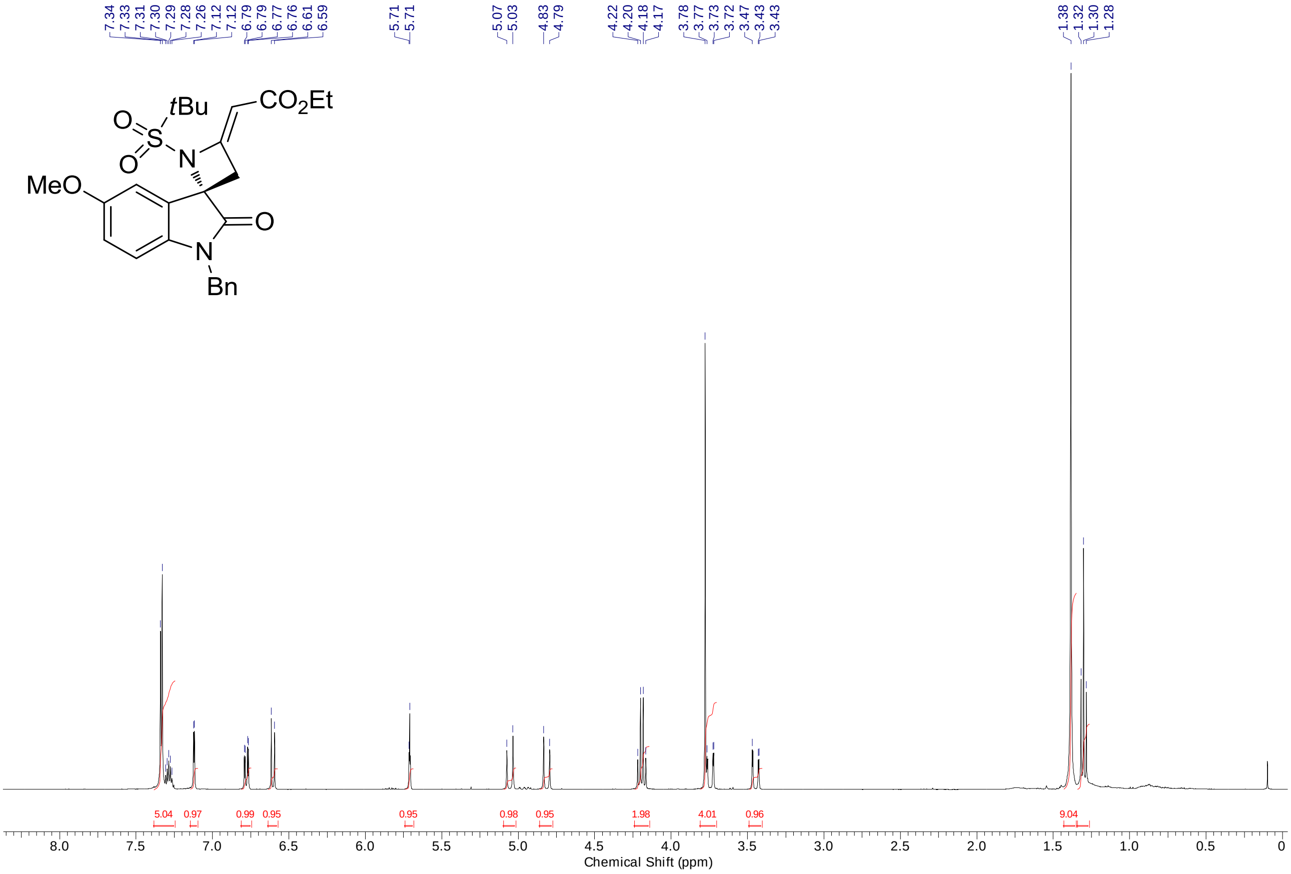

Compound 6g: ^{13}C NMR (101 MHz, APT, $CDCl_3$)

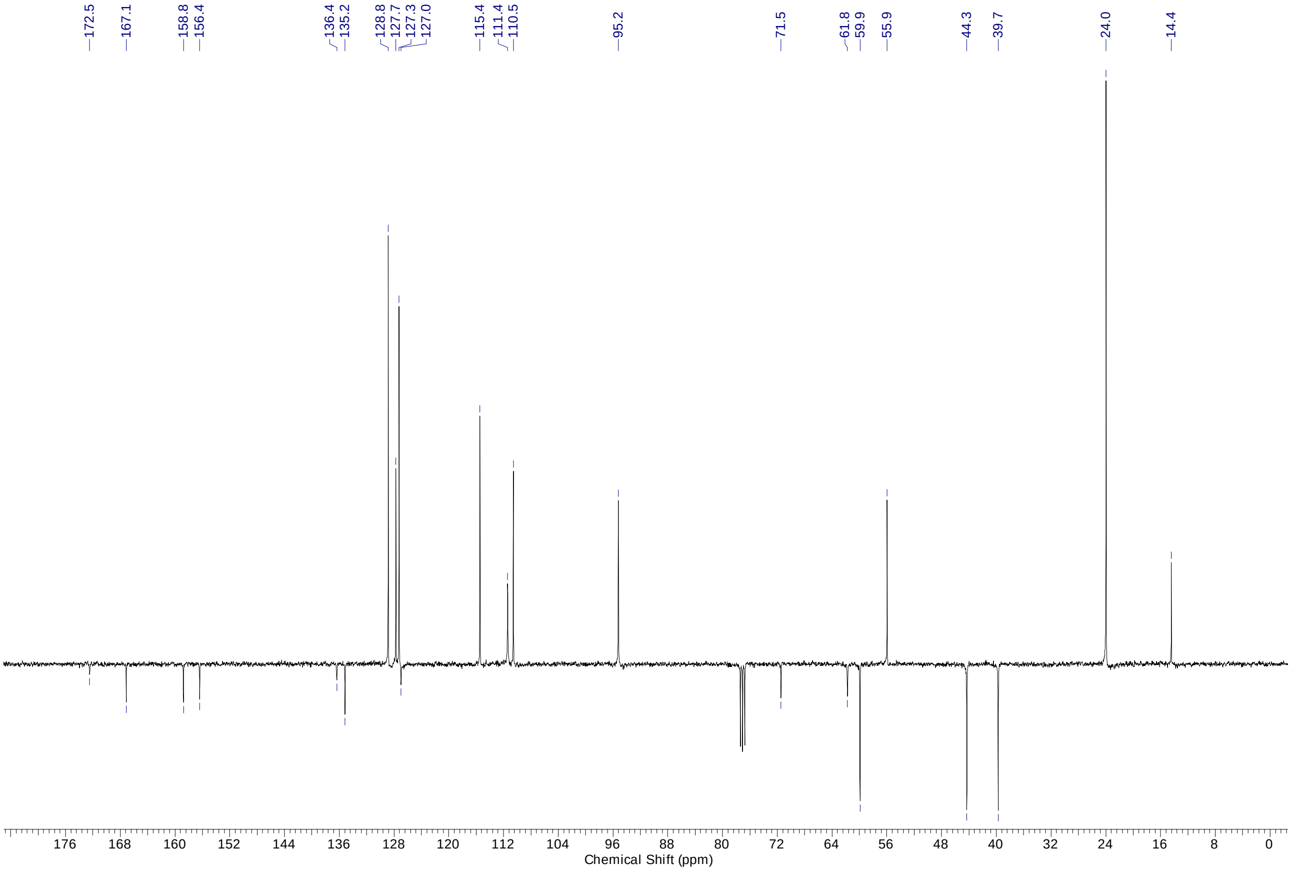

Compound 6h: ^{1}H NMR (400 MHz, $CDCl_3$)

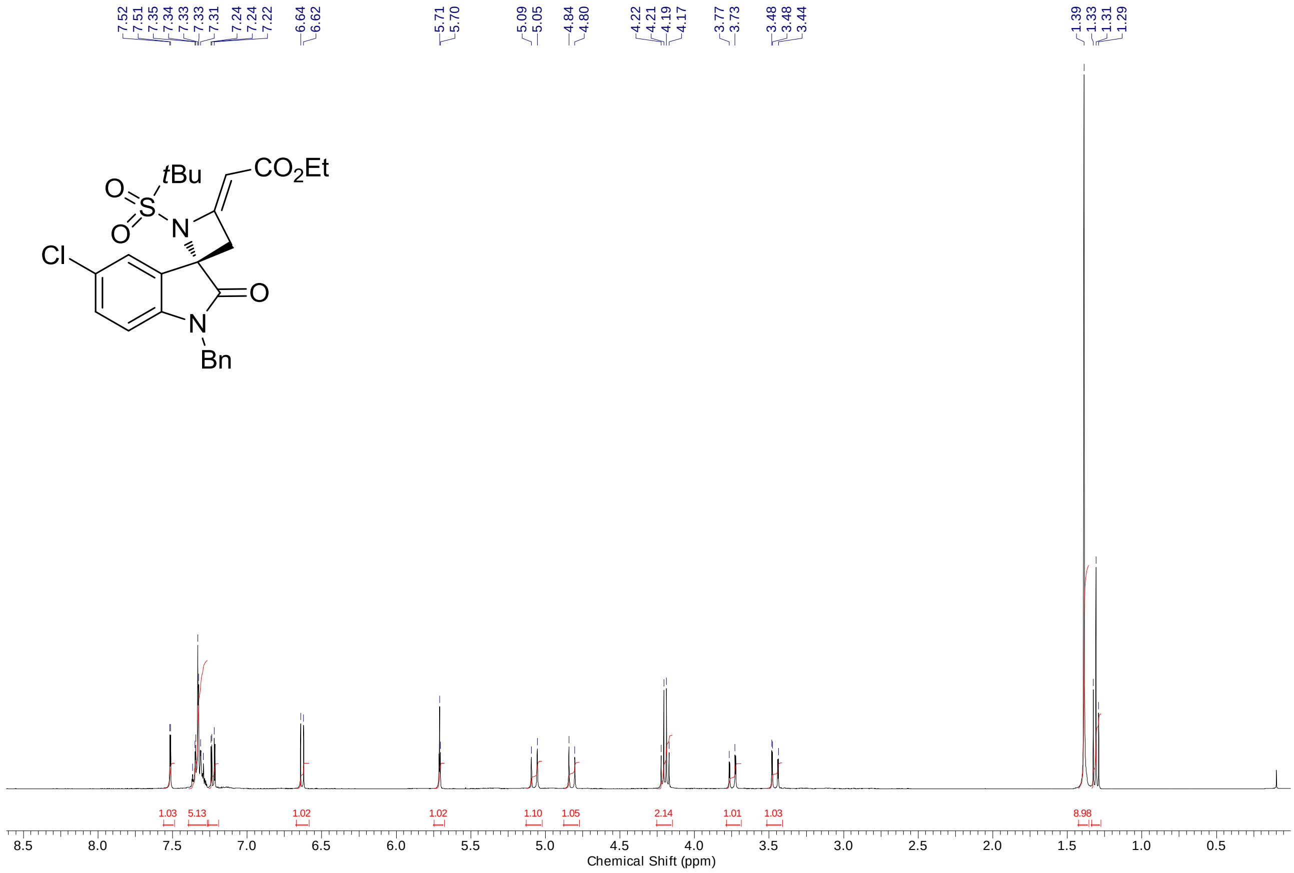

Compound 6h: ^{13}C NMR (101 MHz, APT, $CDCl_3$)

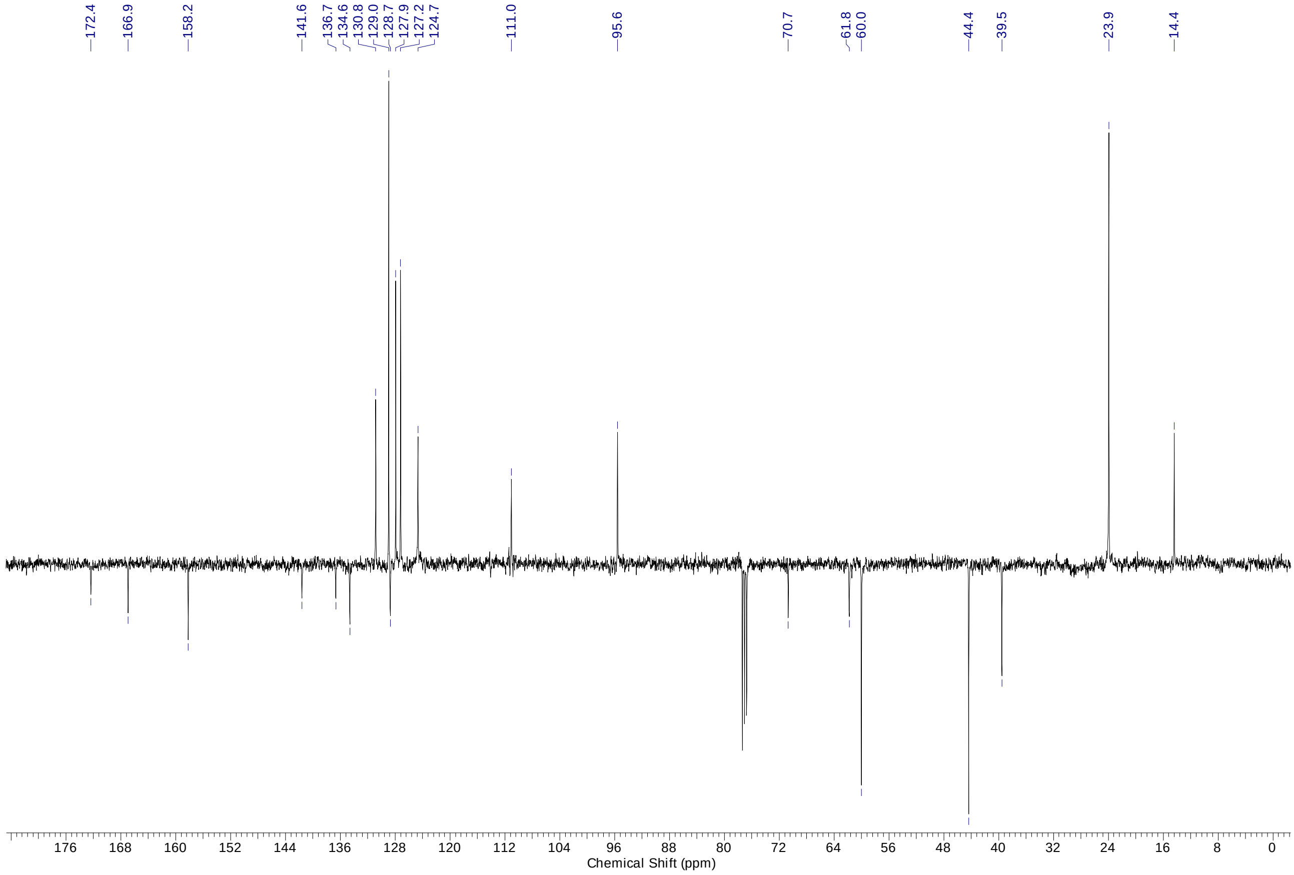

Compound 6k: ^{1}H NMR (400 MHz, $CDCl_3$)

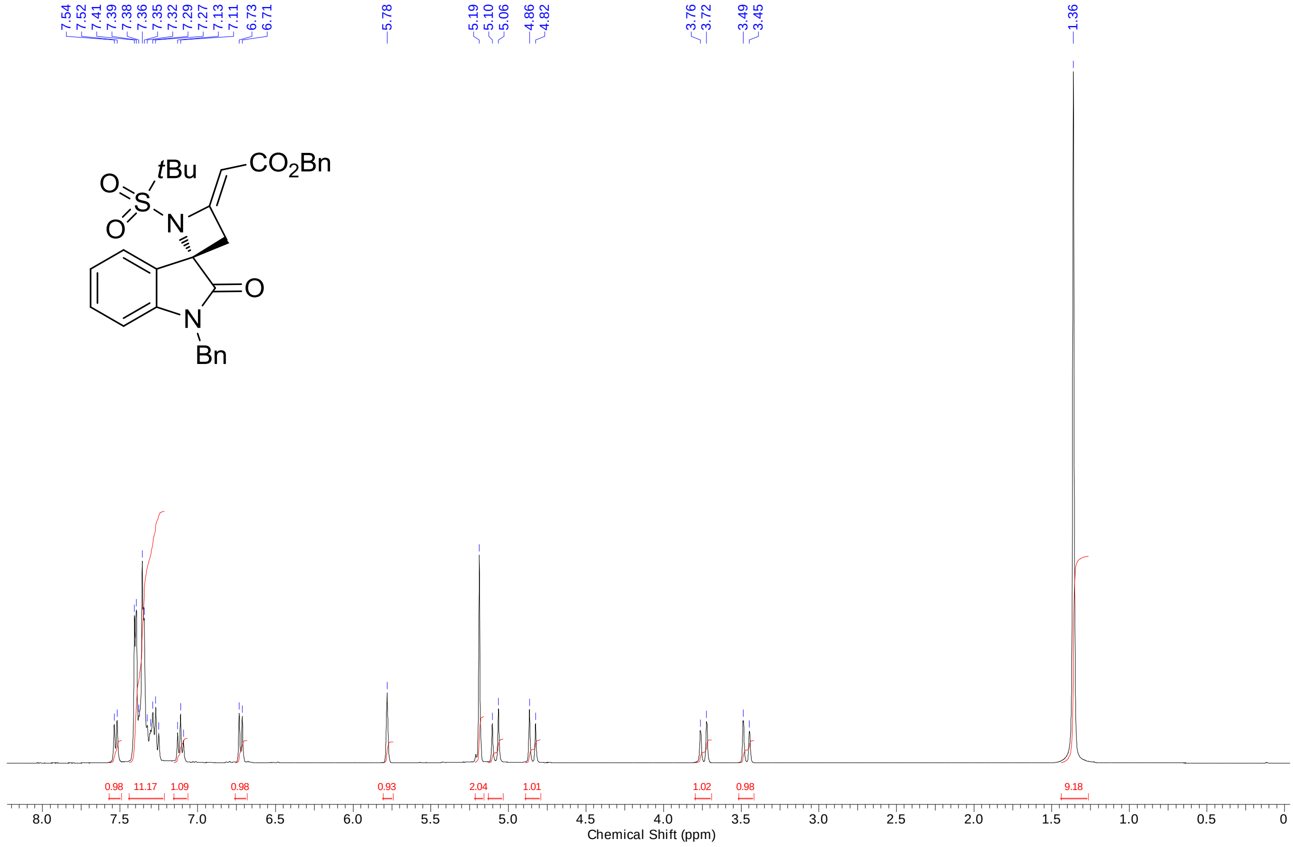

Compound 6k: ^{13}C NMR (101 MHz, APT, $CDCl_3$)

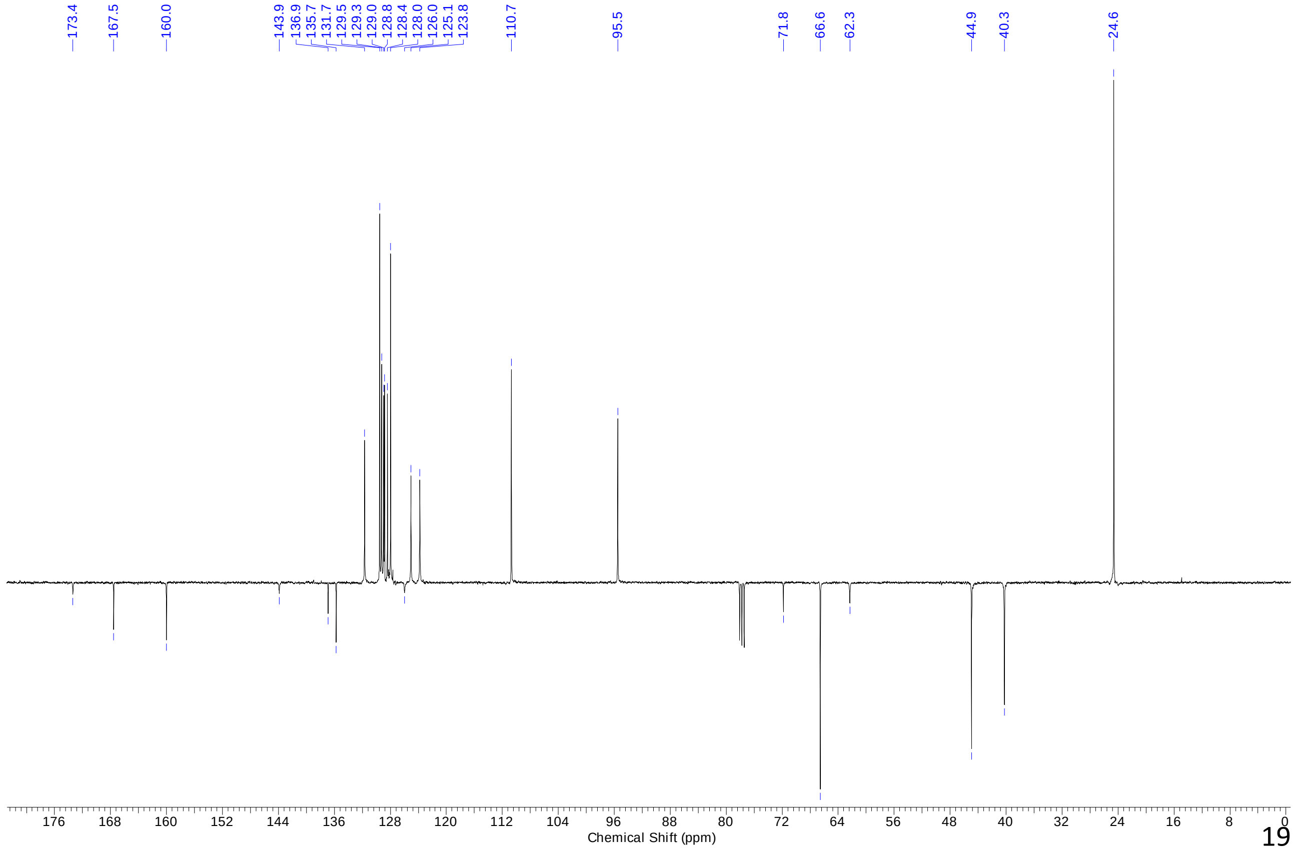

Compound 7: ^{1}H NMR (400 MHz, $CDCl_3$)

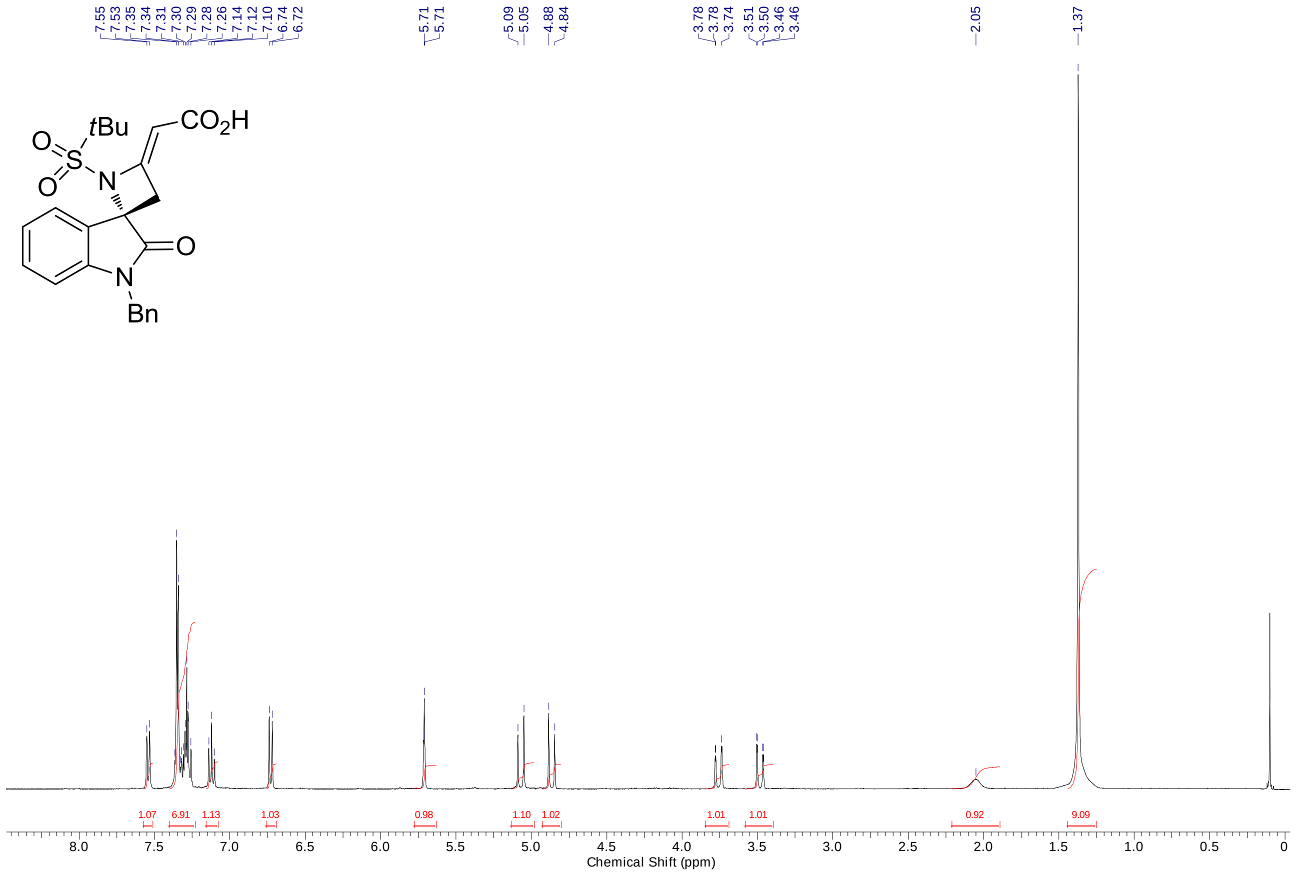

Compound 7: ^{13}C NMR (101 MHz, APT, $CDCl_3$)

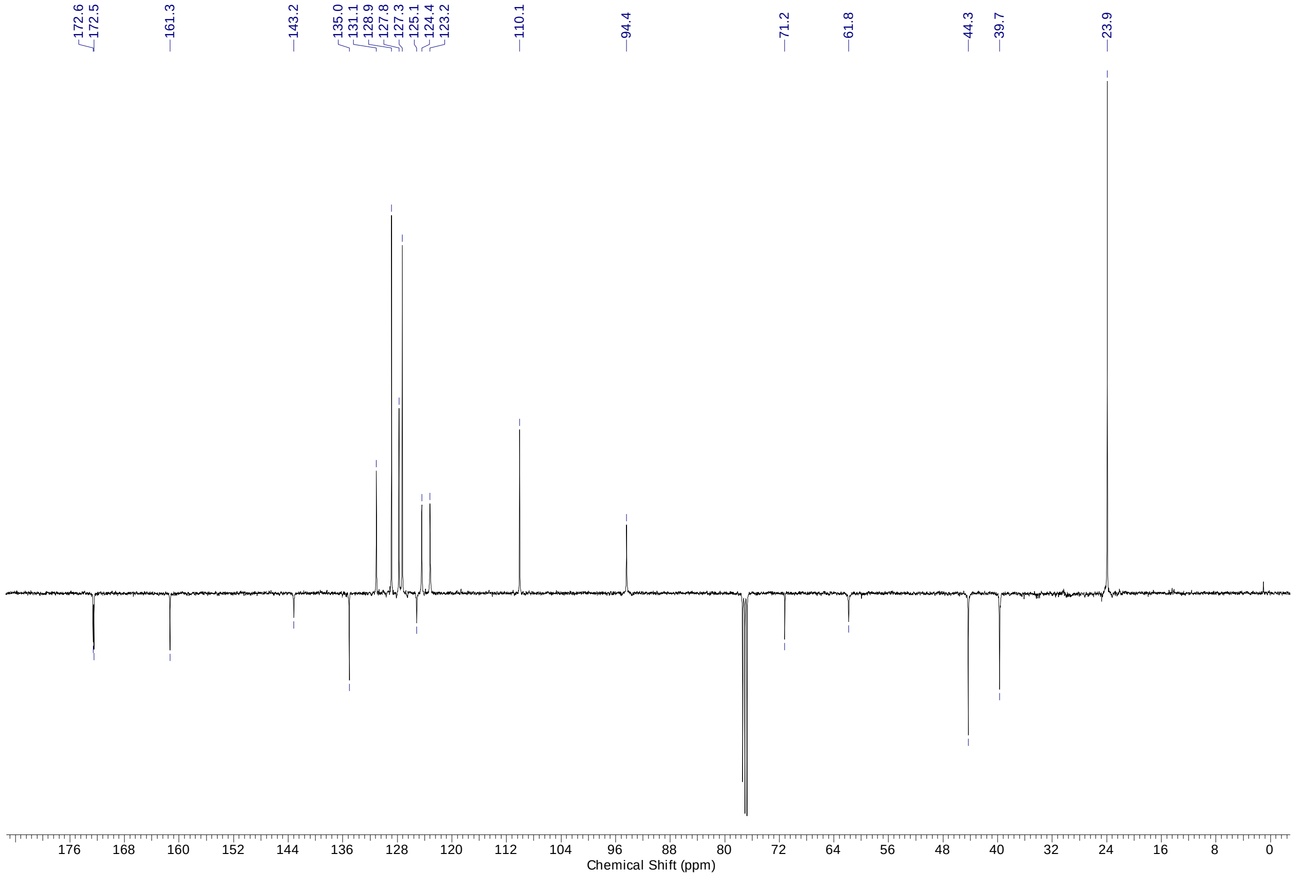

Compound 8: ^{1}H NMR (400 MHz, $CDCl_3$)

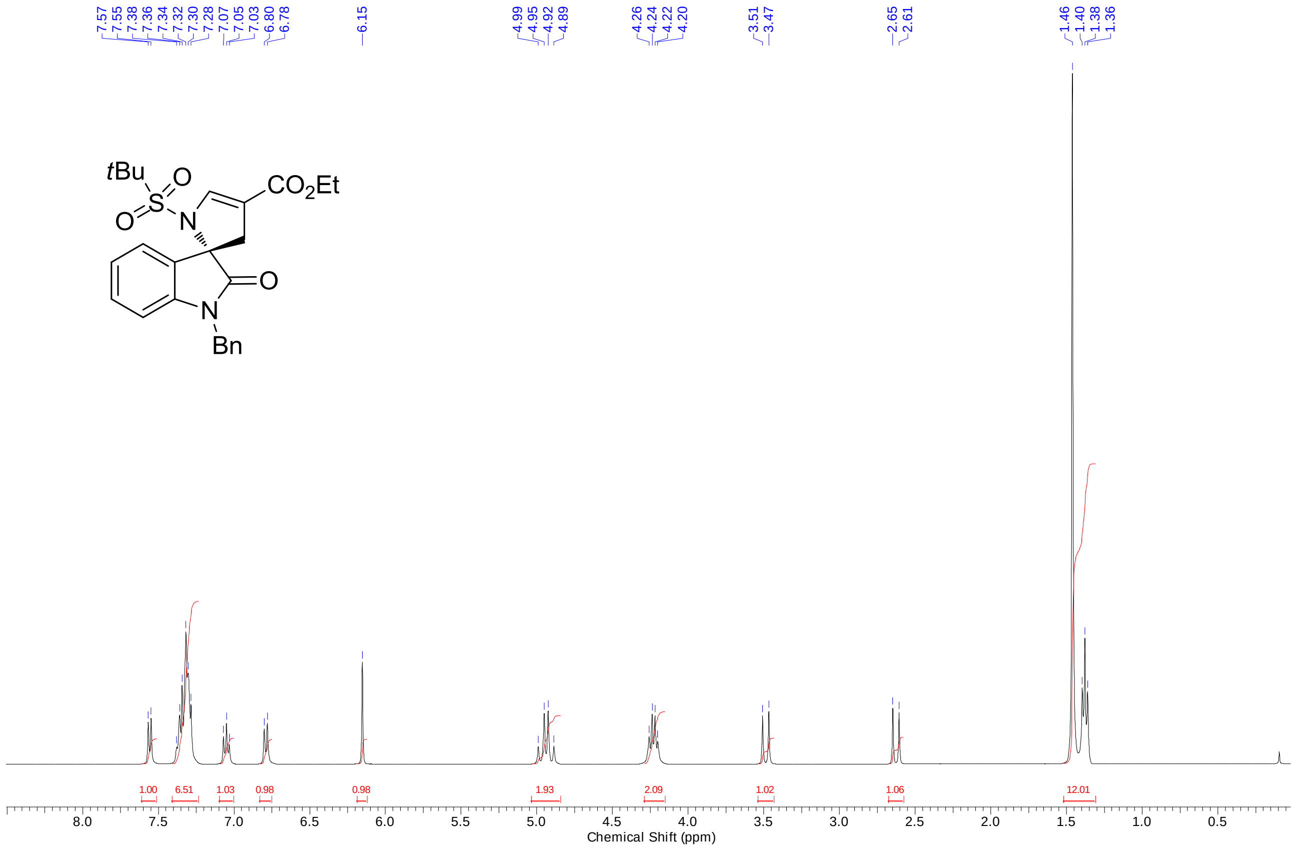

Compound 8: ^{13}C NMR (101 MHz, APT, $CDCl_3$)

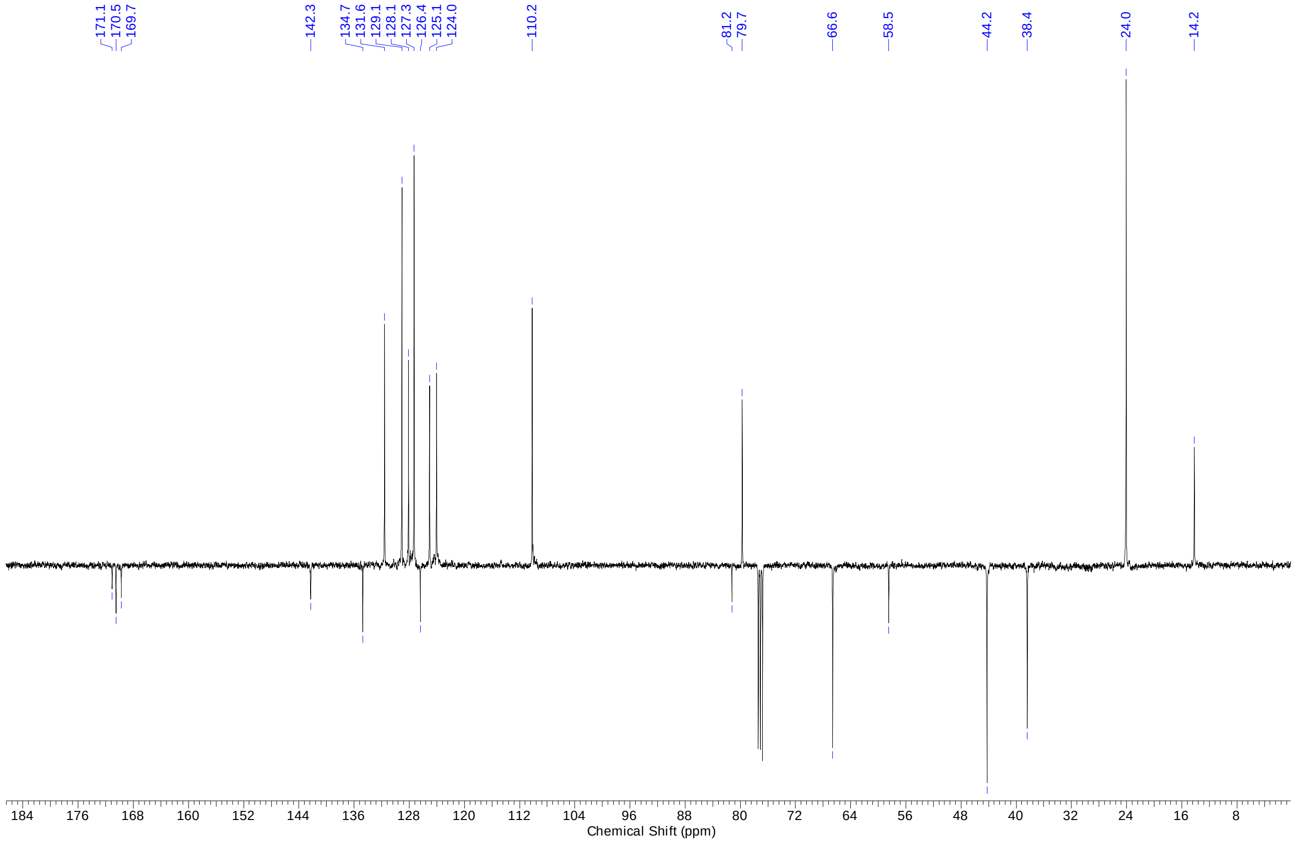

Compound 9: ^{1}H NMR (400 MHz, $CDCl_3$)

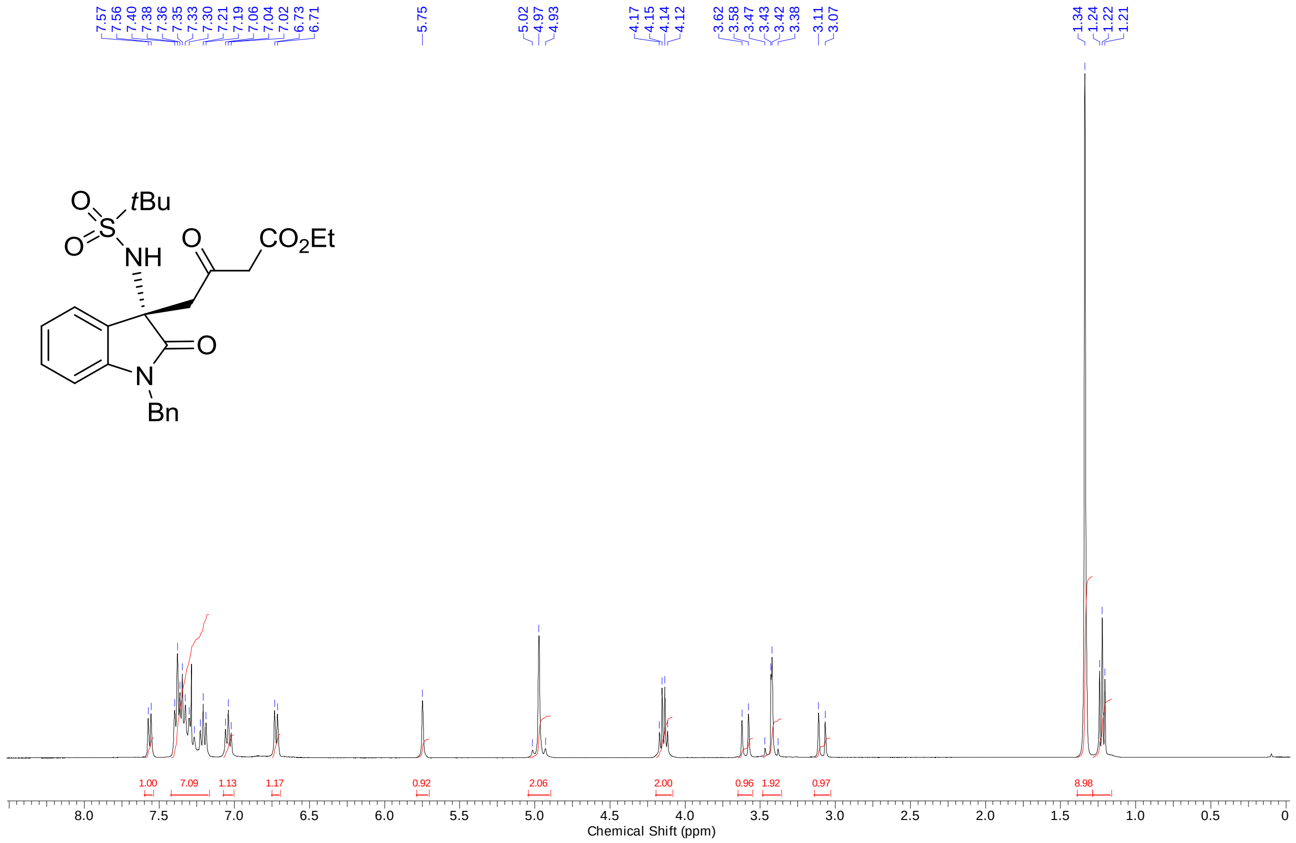

Compound 9: ^{13}C NMR (101 MHz, APT, $CDCl_3$)

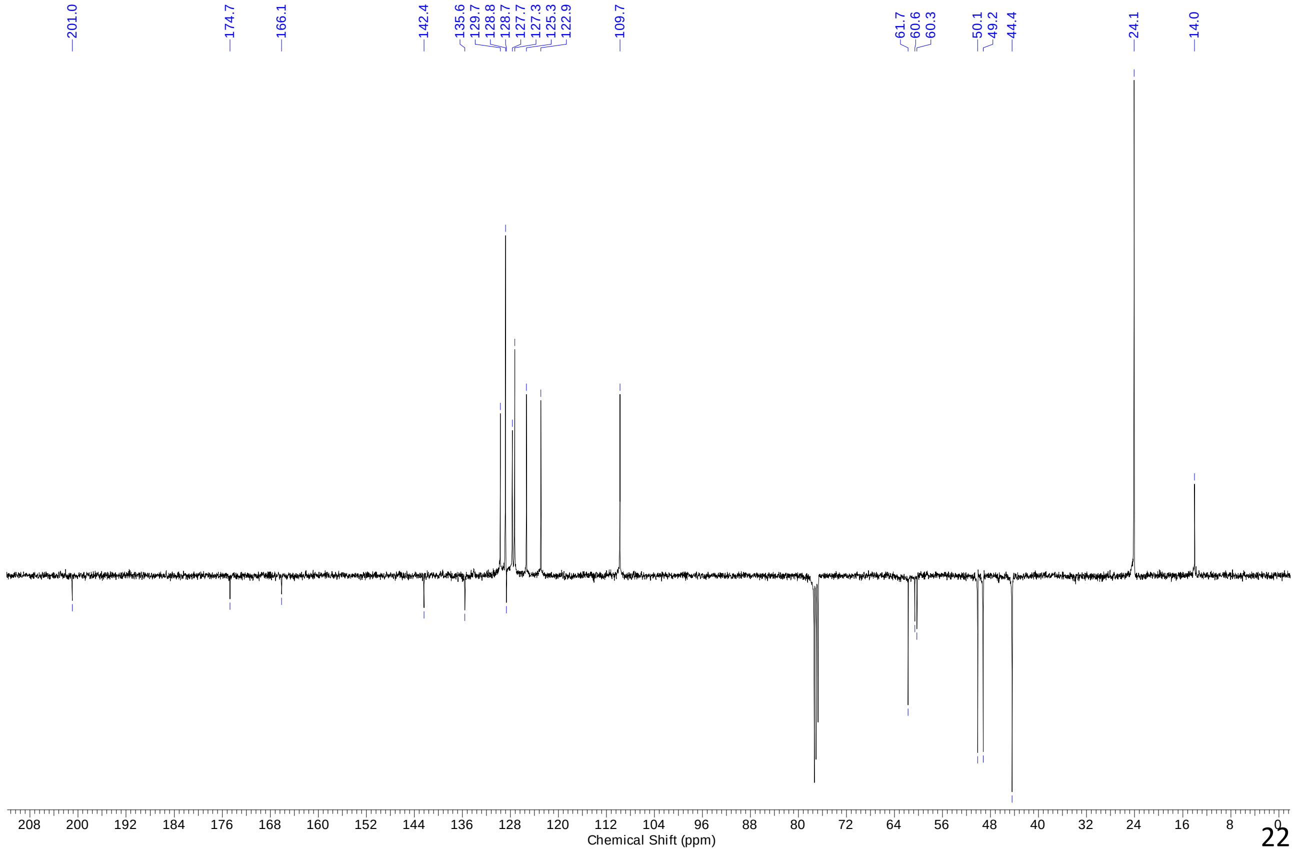

Compound **6a** (racemic mixture) obtained employing DABCO as catalyst

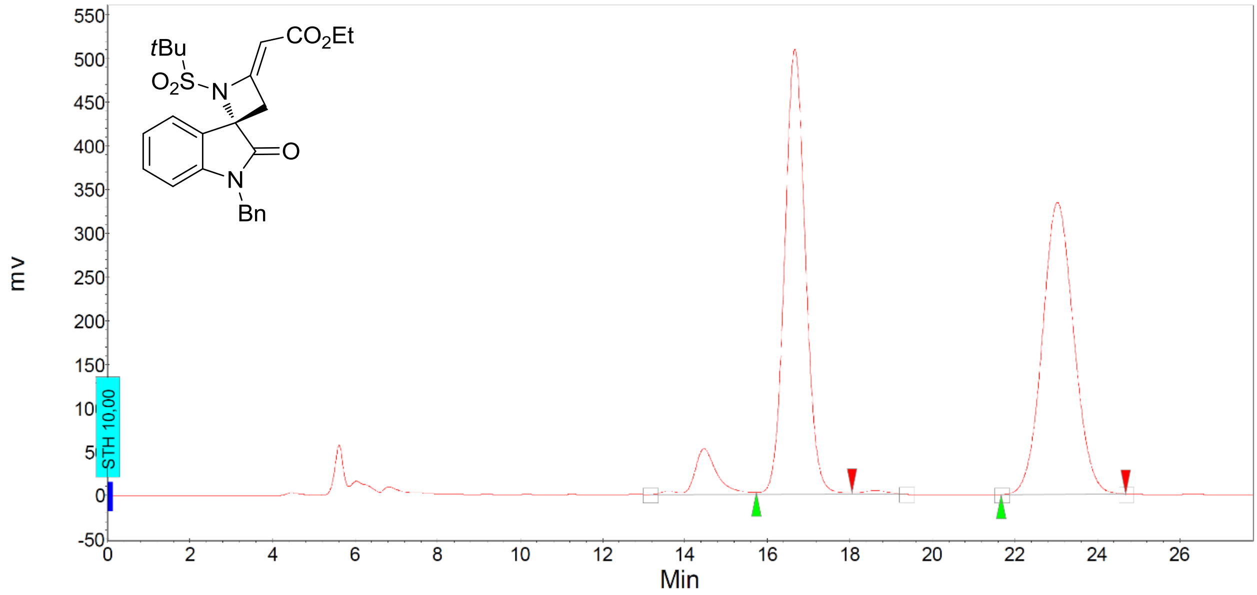

Index	Name	Start	Time	End	Ret. time Offset	Quantity	Height	Area	Area %
		[Min]	[Min]	[Min]	[Min]	[% Area]	[mV]	[mV.Min]	[%]
1	UNKNOWN	15,750	16,675	18,058	0,000	50,29	509,4	298,4	50,286
2	UNKNOWN	21,667	23,033	24,683	0,000	49,71	334,1	295,0	49,714
Total						100,00	843,5	593,5	100,000

Compound **6a** (enantioenriched) obtained employing catalyst **5g**

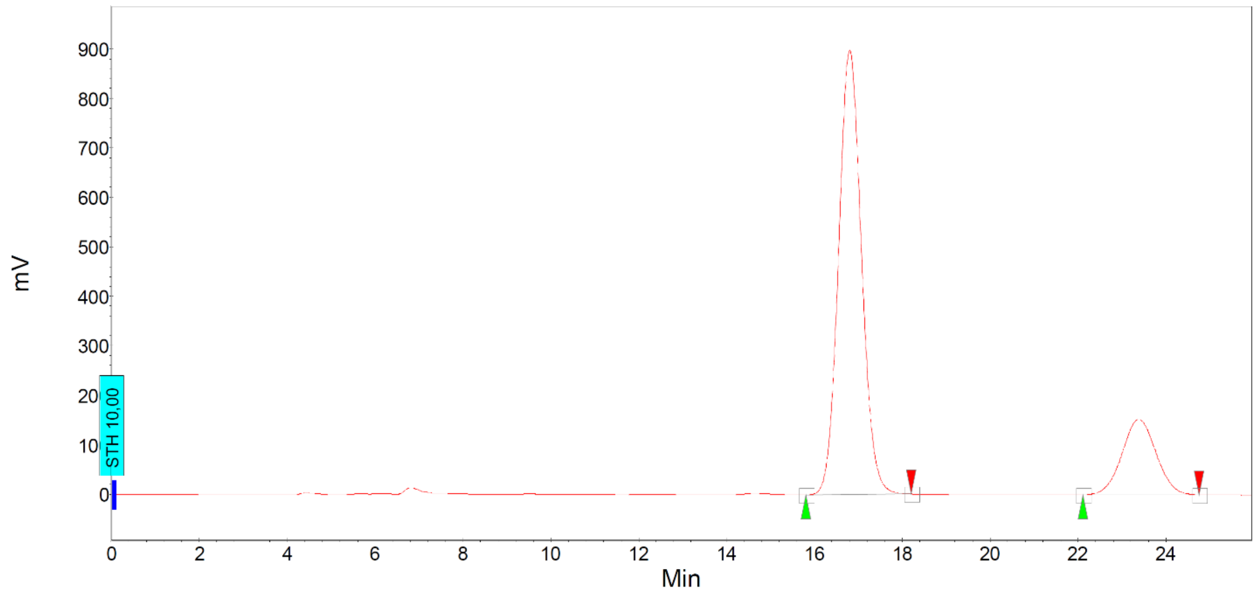

Index	Name	Start	Time	End	Ret. time Offset	Quantity	Height	Area	Area %
		[Min]	[Min]	[Min]	[Min]	[% Area]	[mV]	[mV.Min]	[%]
1	UNKNOWN	15,817	16,808	18,200	0,000	79,83	897,8	542,3	79,826
2	UNKNOWN	22,117	23,383	24,750	0,000	20,17	152,6	137,1	20,174
Total						100,00	1050,3	679,3	100,000

Compound **6a'** obtained through oxidation of compound **I** (see main text, Scheme 1)

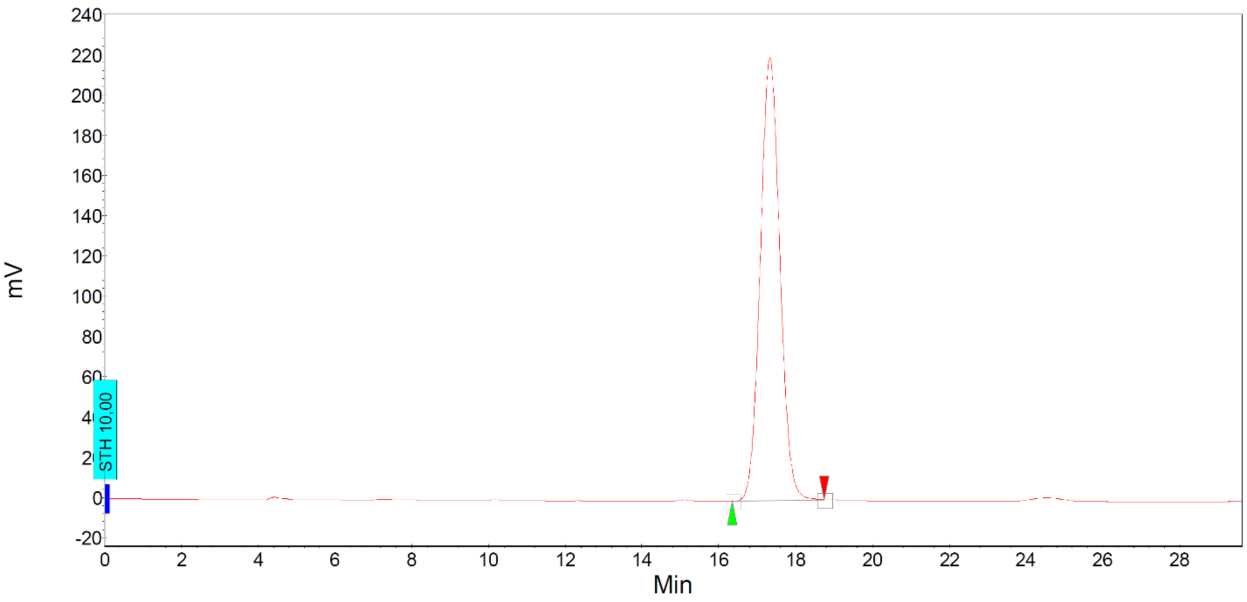

Index	Name	Start	Time	End	Ret. time Offset	Quantity	Height	Area	Area %
		[Min]	[Min]	[Min]	[Min]	[% Area]	[mV]	[mV.Min]	[%]
1	UNKNOWN	16,350	17,333	18,750	0,000	100,00	219,9	130,2	100,000
Total						100,00	219,9	130,2	100,000

Compound **6b** (racemic mixture) obtained employing DABCO as catalyst

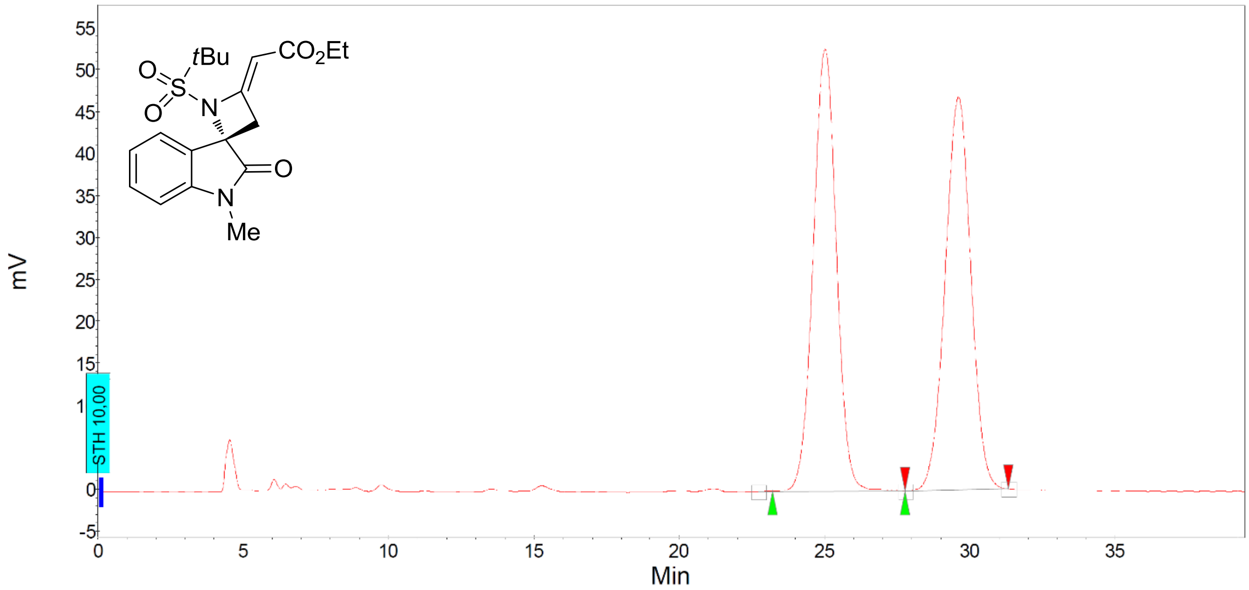

Index	Name	Start	Time	End	Ret. time Offset	Quantity	Height	Area	Area %
		[Min]	[Min]	[Min]	[Min]	[% Area]	[mV]	[mV.Min]	[%]
1	UNKNOWN	23,225	25,025	27,767	0,000	50,35	52,6	48,7	50,348
2	UNKNOWN	27,775	29,608	31,317	0,000	49,65	46,8	48,1	49,652
Total						100,00	99,4	96,8	100,000

Compound **6b** (enantioenriched) obtained employing catalyst **5g**

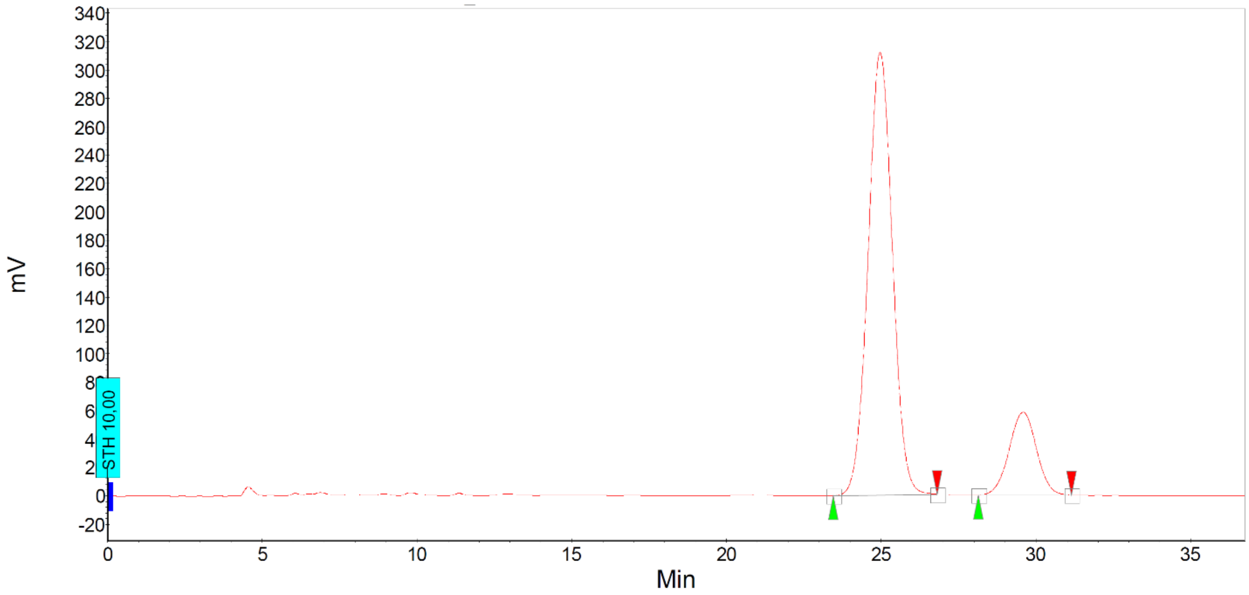

Index	Name	Start	Time	End	Ret. time Offset	Quantity	Height	Area	Area %
		[Min]	[Min]	[Min]	[Min]	[% Area]	[mV]	[mV.Min]	[%]
1	UNKNOWN	23,450	24,958	26,800	0,000	82,63	311,8	284,1	82,634
2	UNKNOWN	28,133	29,575	31,133	0,000	17,37	58,2	59,7	17,366
Total						100,00	370,0	343,8	100,000

Compound **6c** (racemic mixture) obtained employing DABCO as catalyst

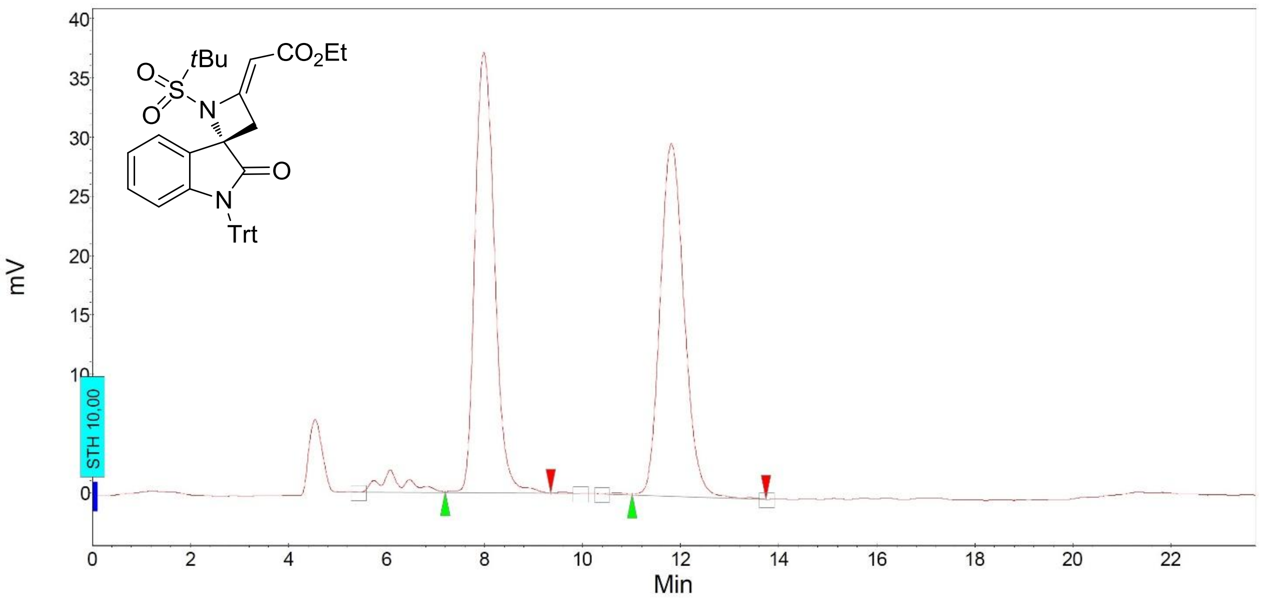

Index	Name	Start	Time	End	Ret. time Offset	Quantity	Height	Area	Area %
		[Min]	[Min]	[Min]	[Min]	[% Area]	[mV]	[mV.Min]	[%]
1	UNKNOWN	7,200	7,983	9,342	0,000	50,55	37,2	17,2	50,551
2	UNKNOWN	11,000	11,817	13,742	0,000	49,45	29,8	16,8	49,449
Total						100,00	66,9	34,1	100,000

Compound **6c** (enantioenriched) obtained employing catalyst **5g**

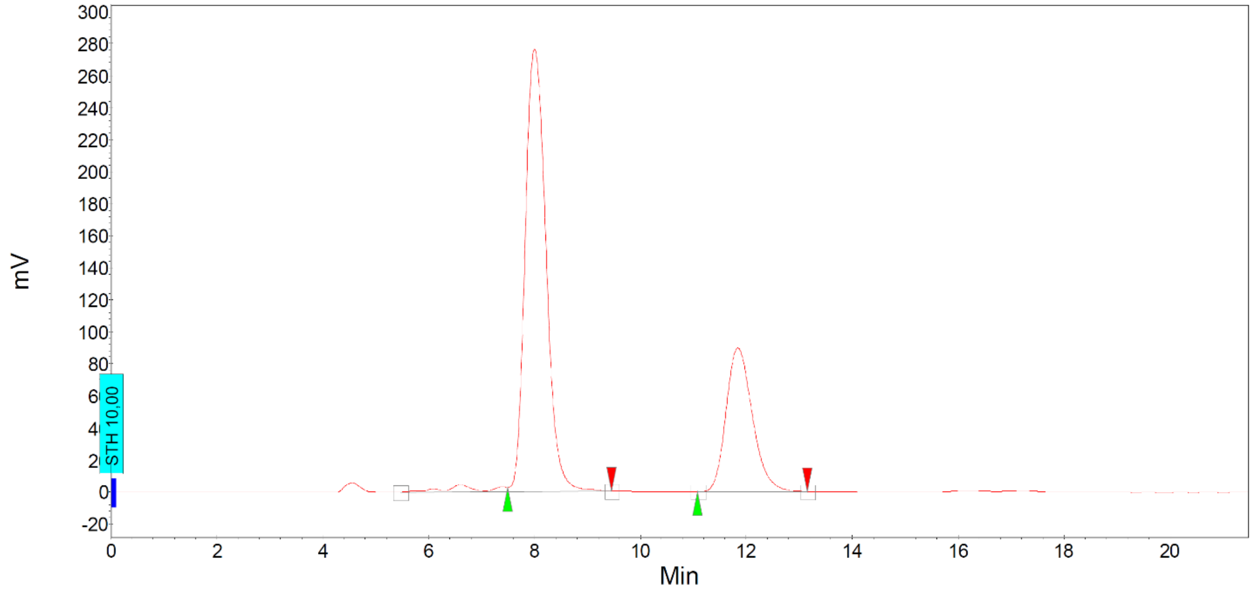

Index	Name	Start	Time	End	Ret. time Offset	Quantity	Height	Area	Area %
		[Min]	[Min]	[Min]	[Min]	[% Area]	[mV]	[mV.Min]	[%]
1	UNKNOWN	7,492	8,000	9,450	0,000	70,84	276,2	125,7	70,843
2	UNKNOWN	11,083	11,842	13,150	0,000	29,16	89,7	51,7	29,157
Total						100,00	365,9	177,4	100,000

Compound **6d** (racemic mixture) obtained employing DABCO as catalyst

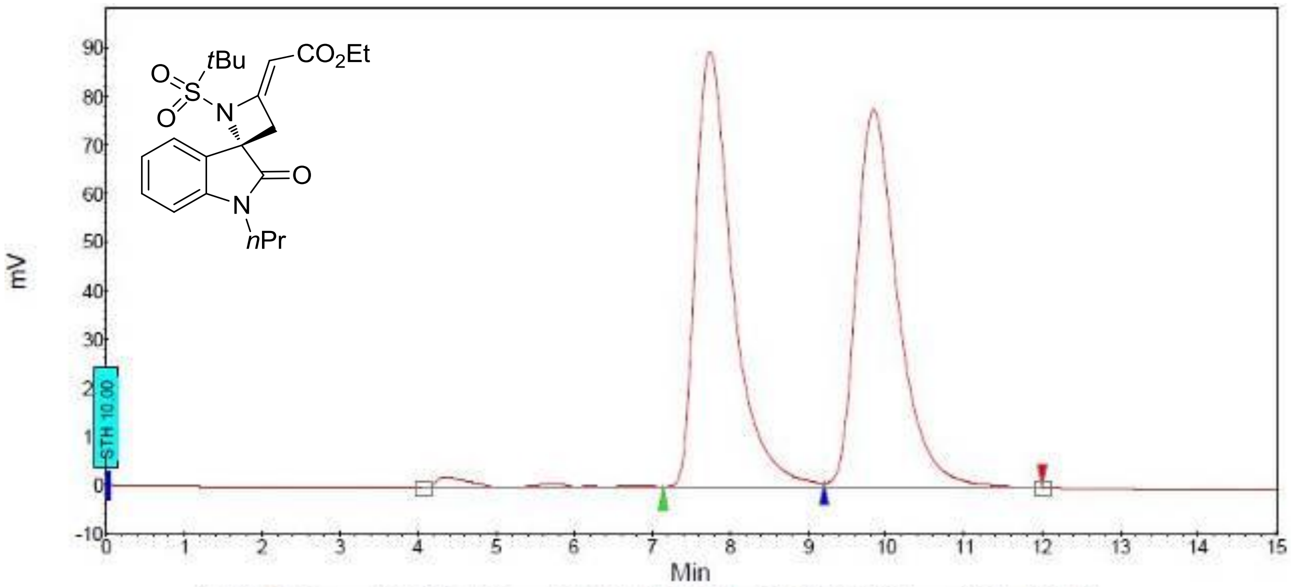

Index	Name	Start	Time	End	Ret. time Offset	Quantity	Height	Area	Area %
		[Min]	[Min]	[Min]	[Min]	[% Area]	[mV]	[mV.Min]	[%]
1	UNKNOWN	7,142	7,742	9,208	0,000	50,63	89,7	49,4	50,629
2	UNKNOWN	9,208	9,842	12,000	0,000	49,37	77,8	48,1	49,371
Total						100,00	167,6	97,5	100,000

Compound **6d** (enantioenriched) obtained employing catalyst **5g**

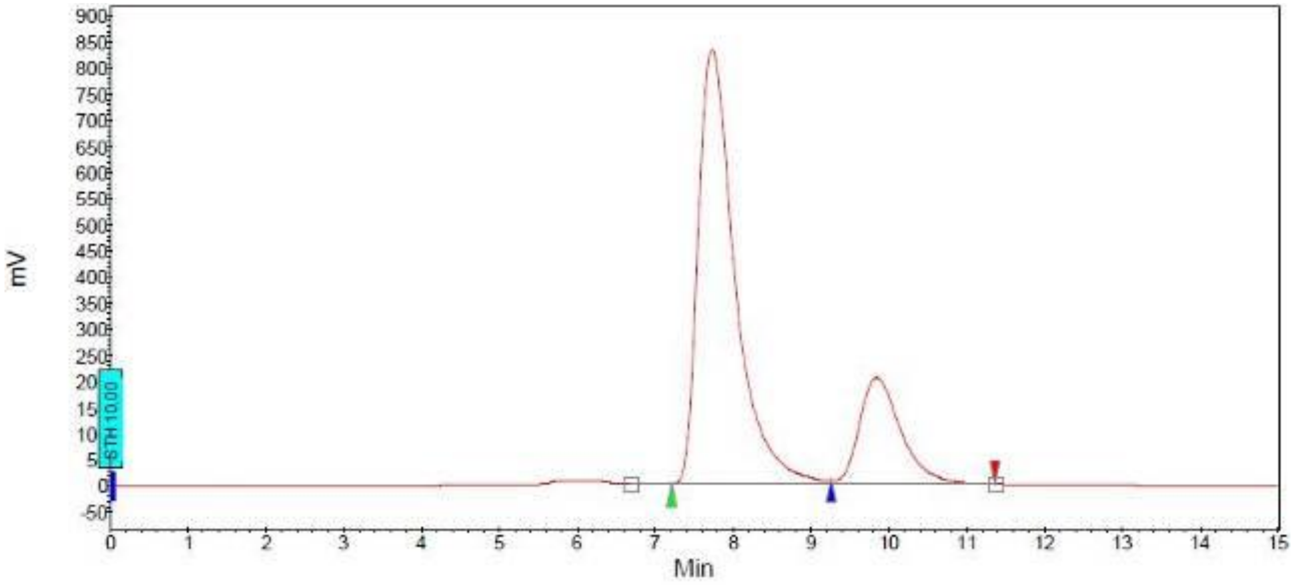

Index	Name	Start	Time	End	Ret. time Offset	Quantity	Height	Area	Area %
		[Min]	[Min]	[Min]	[Min]	[% Area]	[mV]	[mV.Min]	[%]
1	UNKNOWN	7,217	7,733	9,267	0,000	78,42	830,7	457,4	78,415
2	UNKNOWN	9,267	9,850	11,367	0,000	21,58	203,8	125,9	21,585
Total						100,00	1034,5	583,3	100,000

Compound **6e** (racemic mixture) obtained employing DABCO as catalyst

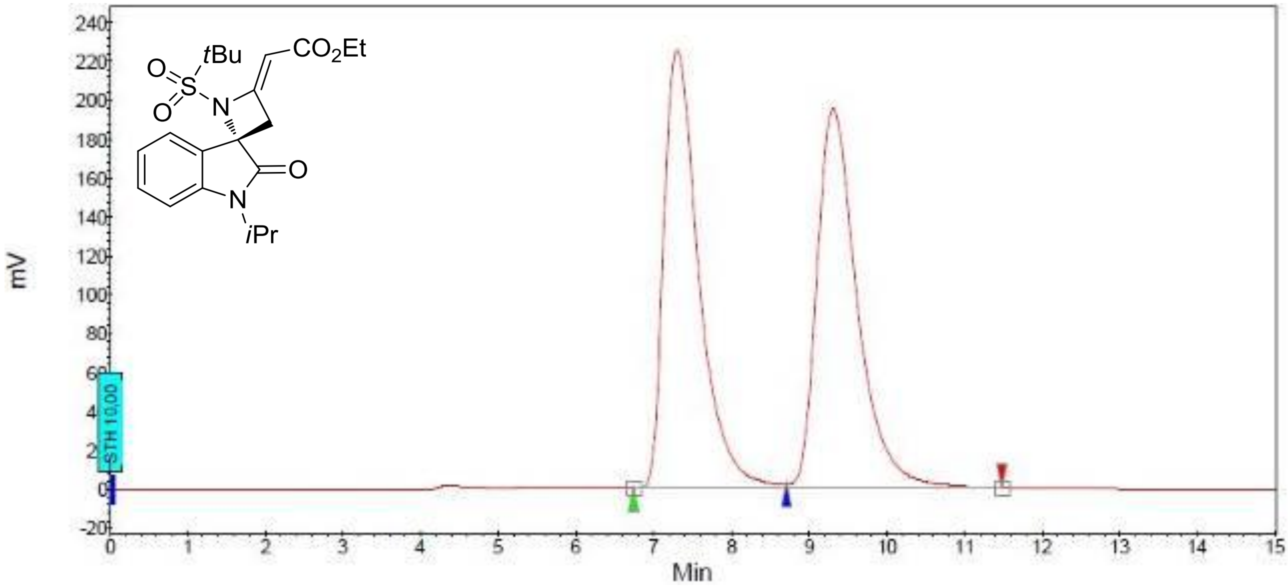

Index	Name	Start	Time	End	Ret. time Offset	Quantity	Height	Area	Area %
		[Min]	[Min]	[Min]	[Min]	[% Area]	[mV]	[mV.Min]	[%]
1	UNKNOWN	6,733	7,308	8,708	0,000	50,18	224,9	118,1	50,177
2	UNKNOWN	8,708	9,317	11,483	0,000	49,82	195,0	117,2	49,823
Total						100,00	419,9	235,3	100,000

Compound **6e** (enantioenriched) obtained employing catalyst **5g**

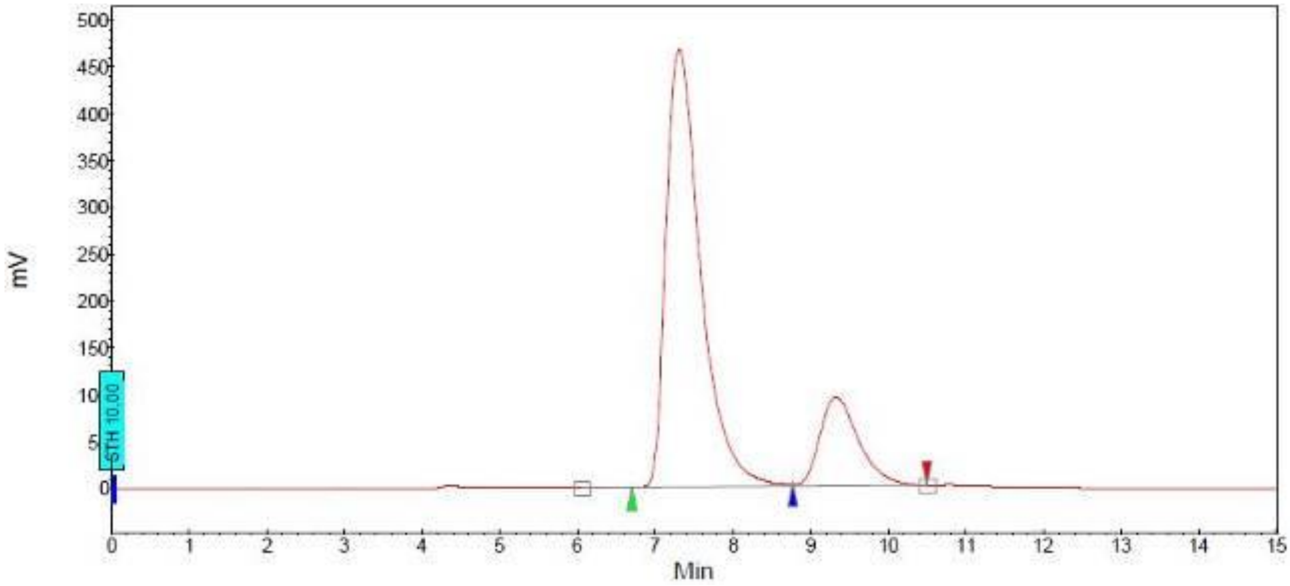

Index	Name	Start	Time	End	Ret. time Offset	Quantity	Height	Area	Area %
		[Min]	[Min]	[Min]	[Min]	[% Area]	[mV]	[mV.Min]	[%]
1	UNKNOWN	6,700	7,317	8,775	0,000	81,54	466,4	242,6	81,541
2	UNKNOWN	8,775	9,333	10,500	0,000	18,46	94,7	54,9	18,459
Total						100,00	561,1	297,6	100,000

Compound **6f** (racemic mixture) obtained employing DABCO as catalyst

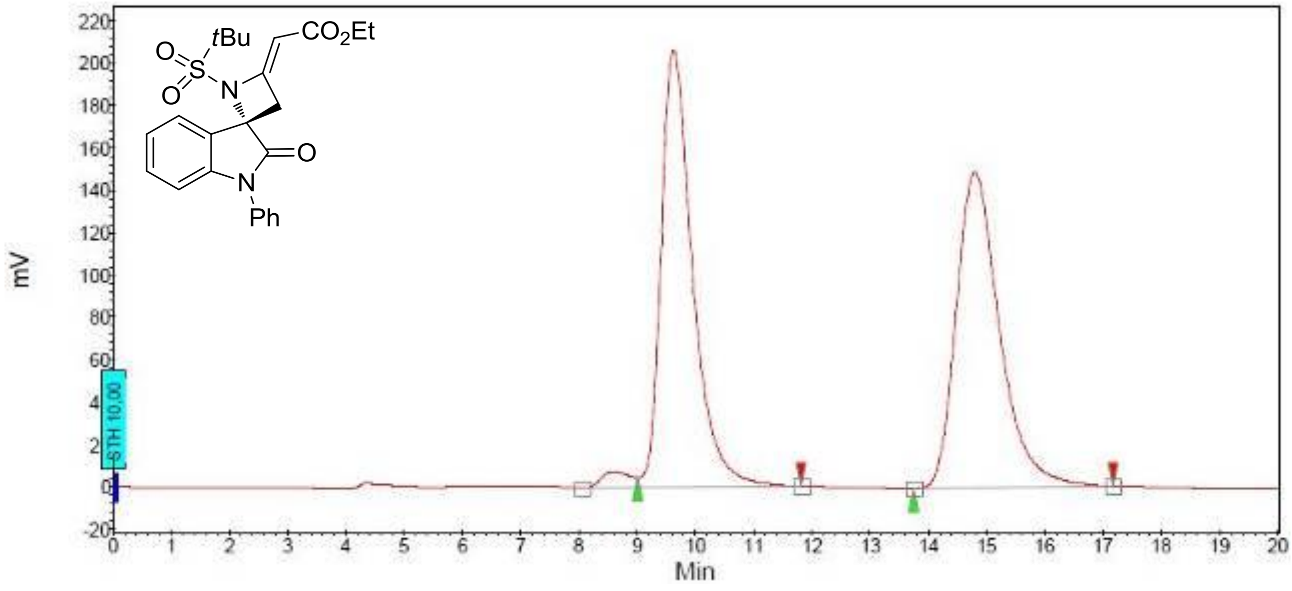

Index	Name	Start	Time	End	Ret. time Offset	Quantity	Height	Area	Area %
		[Min]	[Min]	[Min]	[Min]	[% Area]	[mV]	[mV.Min]	[%]
1	UNKNOWN	9,008	9,633	11,817	0,000	50,49	206,2	130,7	50,487
2	UNKNOWN	13,750	14,792	17,167	0,000	49,51	149,1	128,1	49,513
Total						100,00	355,3	258,8	100,000

Compound **6f** (enantioenriched) obtained employing catalyst **5g**

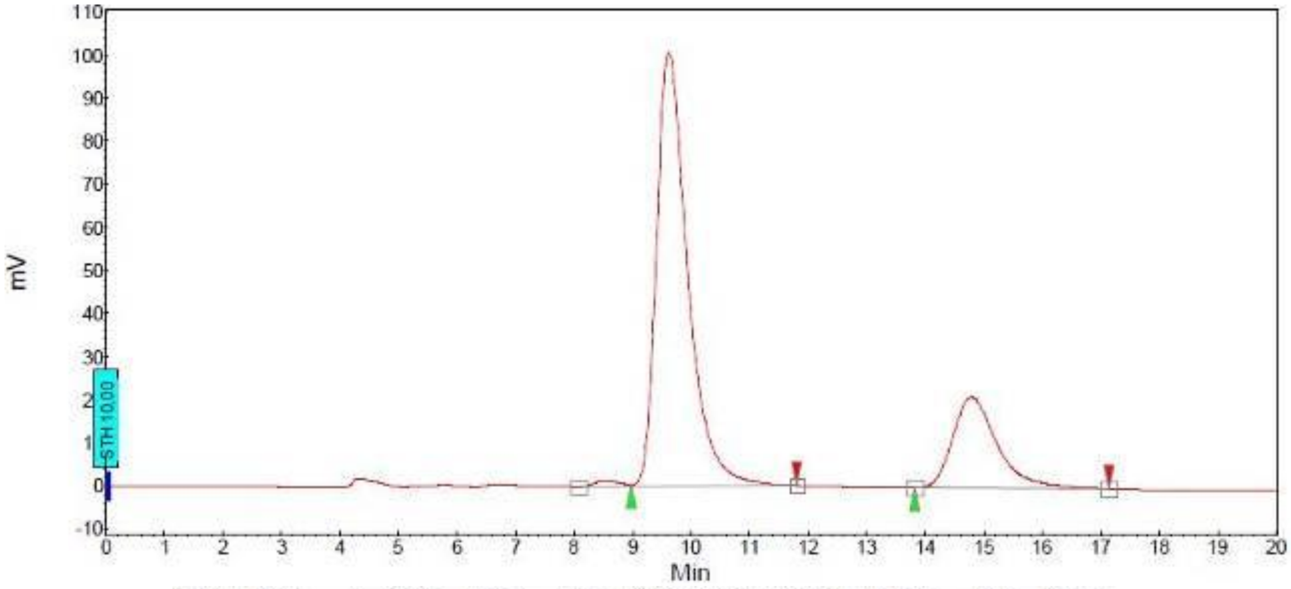

Index	Name	Start	Time	End	Ret. time Offset	Quantity	Height	Area	Area %
		[Min]	[Min]	[Min]	[Min]	[% Area]	[mV]	[mV.Min]	[%]
1	UNKNOWN	8,975	9,625	11,800	0,000	77,29	100,5	62,9	77,292
2	UNKNOWN	13,817	14,792	17,133	0,000	22,71	21,0	18,5	22,708
Total						100,00	121,5	81,4	100,000

Compound **6g** (racemic mixture) obtained employing DABCO as catalyst

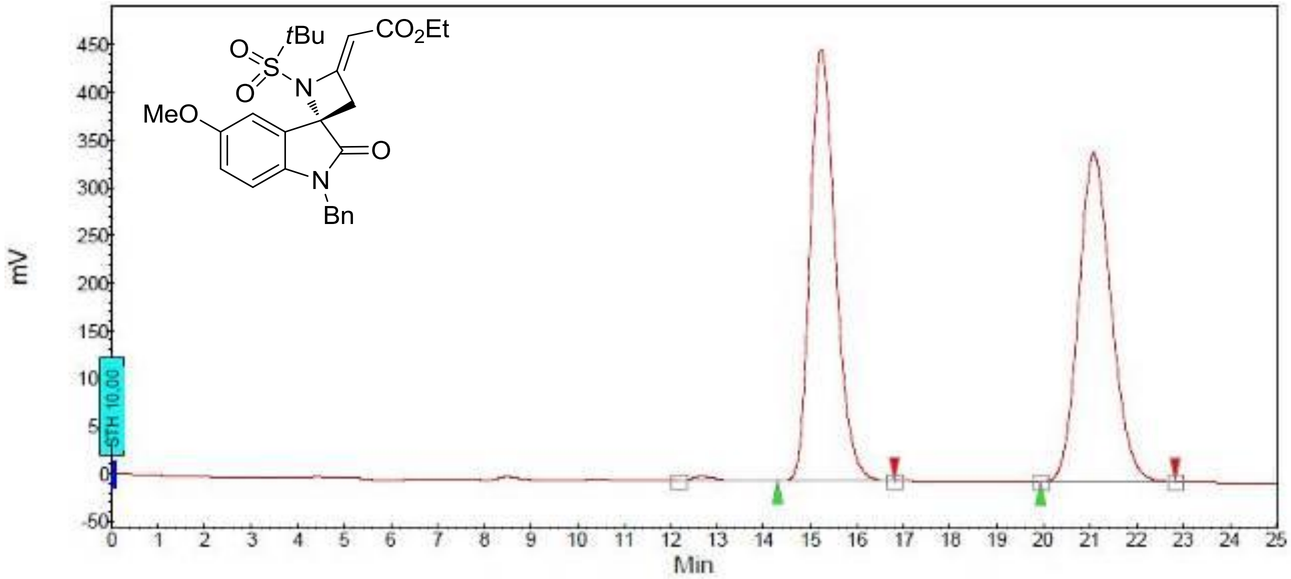

Index	Name	Start	Time	End	Ret. time Offset	Quantity	Height	Area	Area %
		[Min]	[Min]	[Min]	[Min]	[% Area]	[mV]	[mV.Min]	[%]
1	UNKNOWN	14,300	15,242	16,800	0,000	50,26	453,0	283,4	50,257
2	UNKNOWN	19,933	21,075	22,817	0,000	49,74	344,7	280,5	49,743
Total						100,00	797,7	563,9	100,000

Compound **6g** (enantioenriched) obtained employing catalyst **5g**

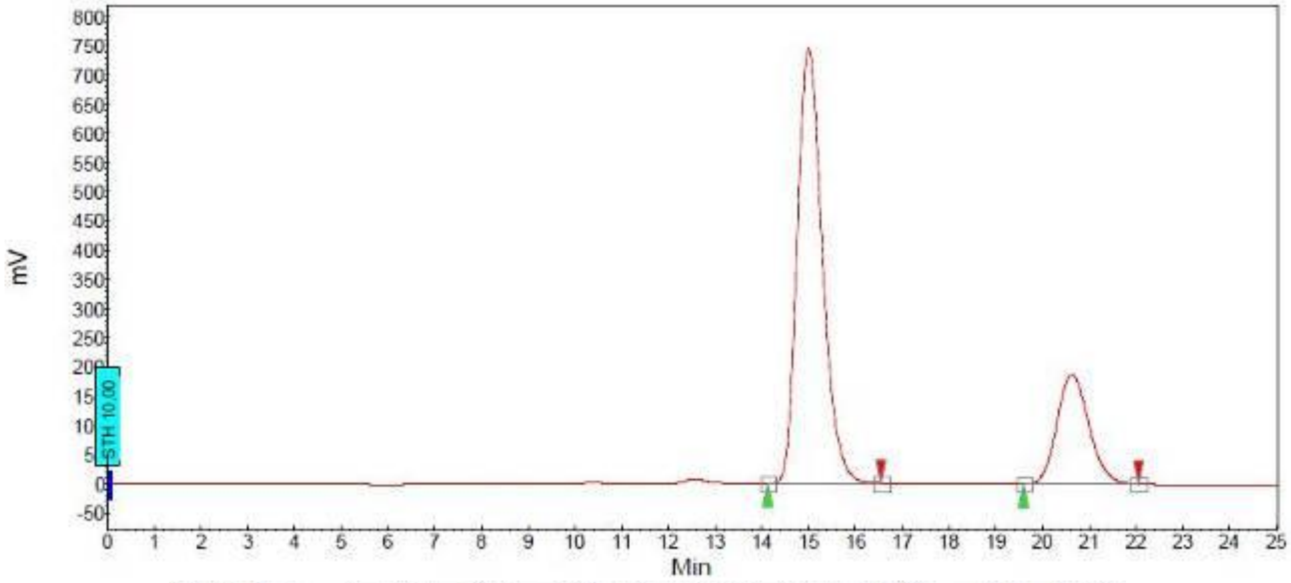

Index	Name	Start	Time	End	Ret. time Offset	Quantity	Height	Area	Area %
		[Min]	[Min]	[Min]	[Min]	[% Area]	[mV]	[mV.Min]	[%]
1	UNKNOWN	14,133	15,000	16,550	0,000	75,74	744,3	462,1	75,745
2	UNKNOWN	19,583	20,625	22,050	0,000	24,26	187,8	148,0	24,255
Total						100,00	932,1	610,0	100,000

Compound **6h** (racemic mixture) obtained employing DABCO as catalyst

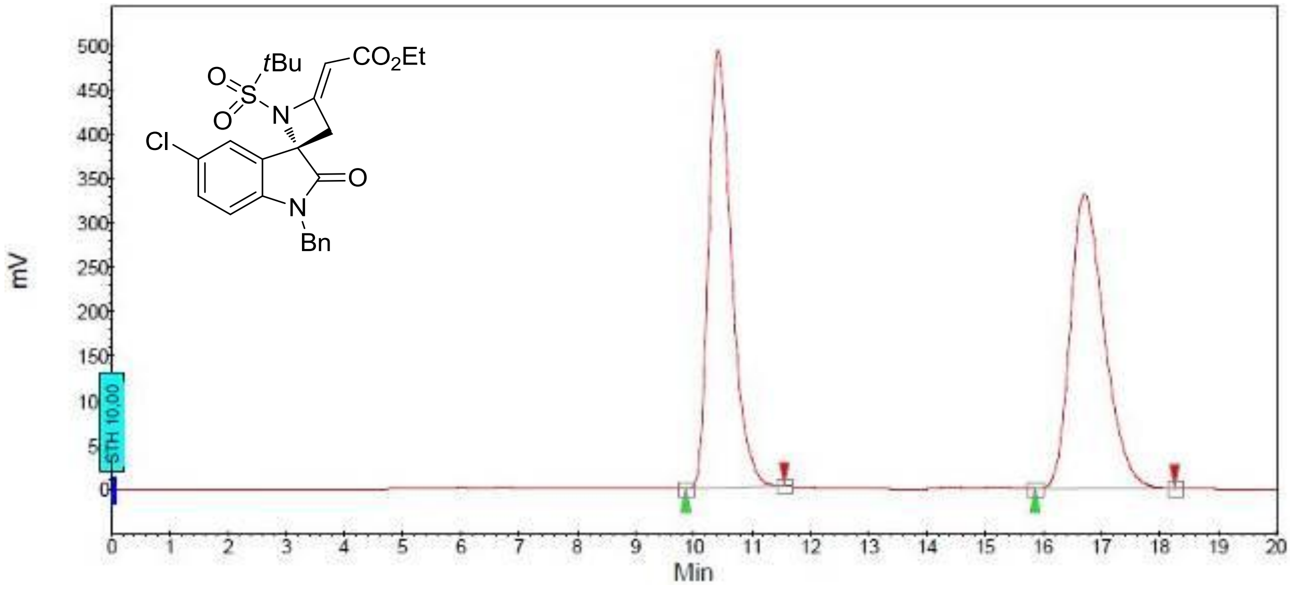

Index	Name	Start	Time	End	Ret. time Offset	Quantity	Height	Area	Area %
		[Min]	[Min]	[Min]	[Min]	[% Area]	[mV]	[mV.Min]	[%]
1	UNKNOWN	9,867	10,417	11,550	0,000	49,97	491,5	224,3	49,965
2	UNKNOWN	15,850	16,700	18,250	0,000	50,03	331,3	224,7	50,035
Total						100,00	822,9	449,0	100,000

Compound **6h** (enantioenriched) obtained employing catalyst **5g**

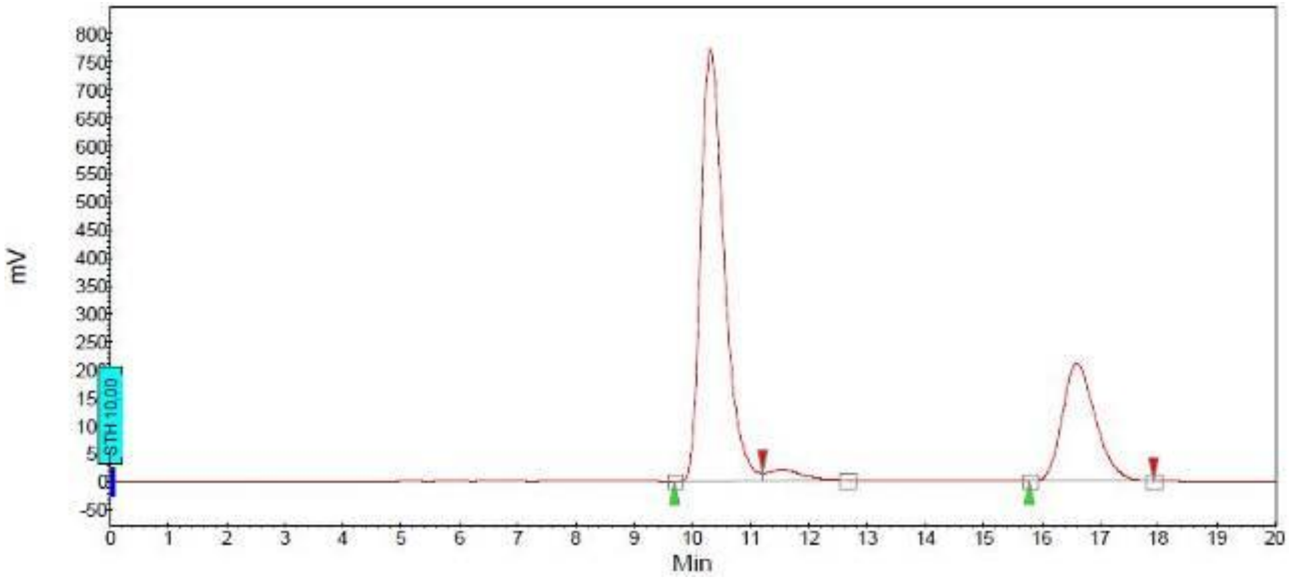

Index	Name	Start	Time	End	Ret. time Offset	Quantity	Height	Area	Area %
		[Min]	[Min]	[Min]	[Min]	[% Area]	[mV]	[mV.Min]	[%]
1	UNKNOWN	9,700	10,308	11,208	0,000	72,19	770,5	359,5	72,193
2	UNKNOWN	15,783	16,592	17,917	0,000	27,81	209,8	138,5	27,807
Total						100,00	980,3	497,9	100,000

Compound **6k** (racemic mixture) obtained employing DABCO as catalyst

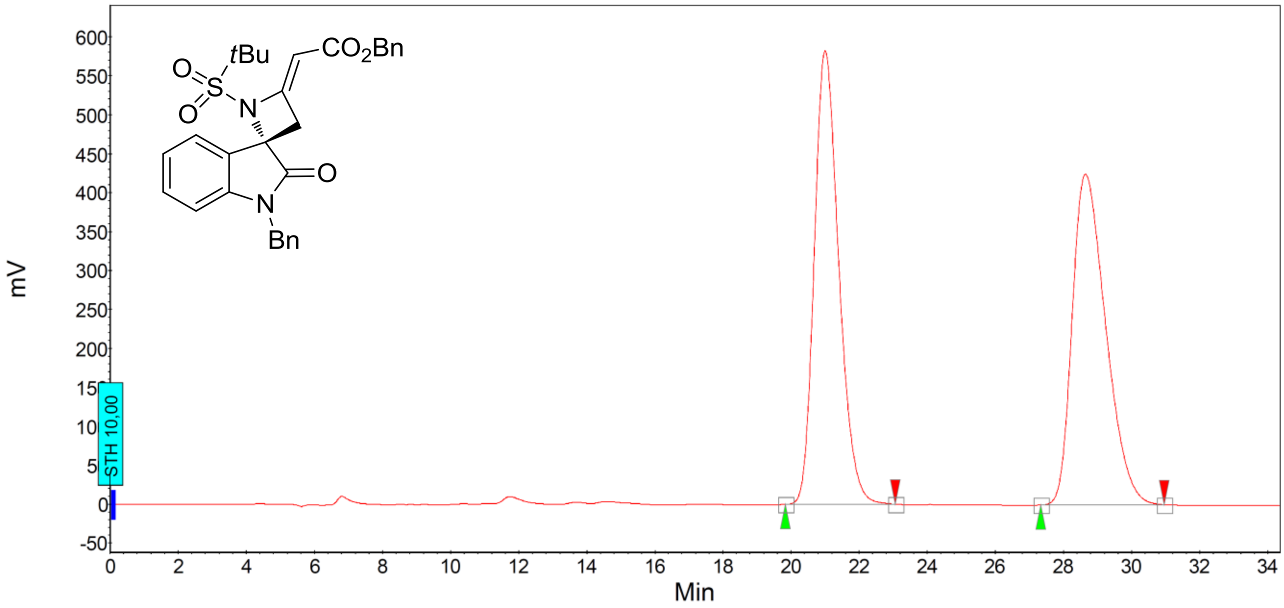

Index	Name	Start	Time	End	Ret. time Offset	Quantity	Height	Area	Area %
		[Min]	[Min]	[Min]	[Min]	[% Area]	[mV]	[mV.Min]	[%]
1	UNKNOWN	19,833	21,008	23,067	0,000	50,06	582,5	484,7	50,063
2	UNKNOWN	27,333	28,642	30,950	0,000	49,94	424,9	483,5	49,937
Total						100,00	1007,5	968,3	100,000

Compound **6k** (enantioenriched) obtained employing catalyst **5g**

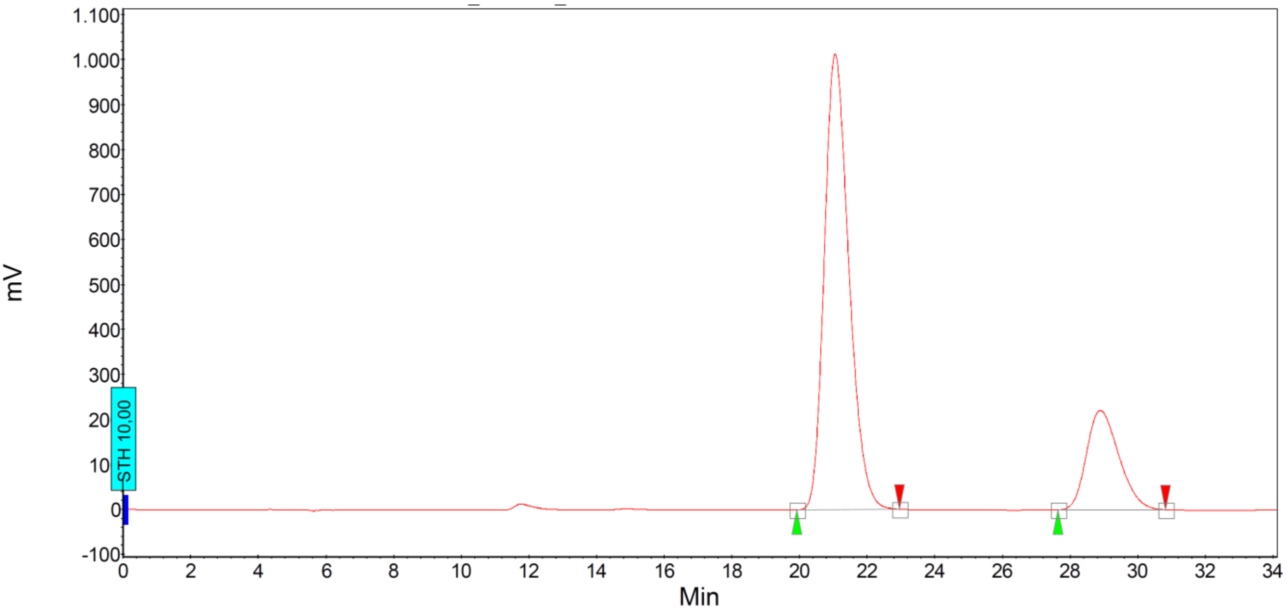

Index	Name	Start	Time	End	Ret. time Offset	Quantity	Height	Area	Area %
		[Min]	[Min]	[Min]	[Min]	[% Area]	[mV]	[mV.Min]	[%]
1	UNKNOWN	19,933	21,058	22,950	0,000	77,68	1012,9	851,7	77,677
2	UNKNOWN	27,633	28,892	30,817	0,000	22,32	220,6	244,8	22,323
Total						100,00	1233,5	1096,5	100,000
